# Health consequences of exposure to aircraft contaminated air and fume events: a narrative review and medical protocol for the investigation of exposed aircrew and passengers

**DOI:** 10.1186/s12940-023-00987-8

**Published:** 2023-05-16

**Authors:** Jonathan Burdon, Lygia Therese Budnik, Xaver Baur, Gerard Hageman, C. Vyvyan Howard, Jordi Roig, Leonie Coxon, Clement E. Furlong, David Gee, Tristan Loraine, Alvin V. Terry, John Midavaine, Hannes Petersen, Denis Bron, Colin L. Soskolne, Susan Michaelis

**Affiliations:** 1 Respiratory Physician, St Vincent’s Private Hospital, East Melbourne, Australia; 2 Institute for Occupational and Maritime Medicine, University Medical Center Hamburg-Eppendorf, Hamburg, Germany; 3 European Society for Environmental and Occupational Medicine, Berlin, Germany; 4 University of Hamburg, Hamburg, Germany; 5 Department of Neurology, Medisch Spectrum Twente, Hospital Enschede, Enschede, The Netherlands; 6 Centre for Molecular Biosciences, University of Ulster, Coleraine, Northern Ireland UK; 7 Department of Pulmonary Medicine, Clínica Creu Blanca, Barcelona, Spain; 8 Clinical and Forensic Psychologist, Mount Pleasant Psychology, Perth, Australia; 9 Departments of Medicine (Div. Medical Genetics) and Genome Sciences, University of Washington, Seattle, USA; 10 Centre for Pollution Research and Policy, Visiting Fellow, Brunel University, London, UK; 11 Technical Consultant, Spokesperson for the Global Cabin Air Quality Executive, London, UK; 12 Department of Pharmacology and Toxicology, Medical College of Georgia, Augusta University, Augusta, USA; 13 Lifemedic Bilthoven, Bilthoven, The Netherlands; 14 Faculty of Medicine, University of Iceland, Akureyri Hospital, Akureyri, Iceland; 15 Federal Department of Defence, Civil Protection and Sport (DDPS), Aeromedical Institute (FAI)/AeMC, Air Force, Dübendorf, Switzerland; 16 School of Public Health, University of Alberta, Edmonton, AB Canada; 17 Occupational and Environmental Health Research Group, Honorary Senior Research Fellow, University of Stirling, Scotland / Michaelis Aviation Consulting, West Sussex, England

**Keywords:** Aerotoxic syndrome, Fume events, Oil fumes, Cabin air, Bleed air, Aircrew, Organophosphates

## Abstract

Thermally degraded engine oil and hydraulic fluid fumes contaminating aircraft cabin air conditioning systems have been well documented since the 1950s. Whilst organophosphates have been the main subject of interest, oil and hydraulic fumes in the air supply also contain ultrafine particles, numerous volatile organic hydrocarbons and thermally degraded products. We review the literature on the effects of fume events on aircrew health. Inhalation of these potentially toxic fumes is increasingly recognised to cause acute and long-term neurological, respiratory, cardiological and other symptoms. Cumulative exposure to regular small doses of toxic fumes is potentially damaging to health and may be exacerbated by a single higher-level exposure. Assessment is complex because of the limitations of considering the toxicity of individual substances in complex heated mixtures.

There is a need for a systematic and consistent approach to diagnosis and treatment of persons who have been exposed to toxic fumes in aircraft cabins. The medical protocol presented in this paper has been written by internationally recognised experts and presents a consensus approach to the recognition, investigation and management of persons suffering from the toxic effects of inhaling thermally degraded engine oil and other fluids contaminating the air conditioning systems in aircraft, and includes actions and investigations for in-flight, immediately post-flight and late subsequent follow up.

**Supplementary Information:**

The online version contains supplementary material available at 10.1186/s12940-023-00987-8.

## Background

All modern commercial jet transport aircraft, except for the Boeing 787 Dreamliner, use air compressed within the engine or auxiliary power unit (a smaller engine primarily used during ground operations, APU) as the source of the air used for aircraft ventilation and pressurisation. This air is known as ‘bleed air’ because it is bled off the compression section of the engine or APU. The engine and APU supply air is not filtered. The use of oil bearing seals reliant on pressurised air enables low levels of synthetic engine oils to enter the compressor air during normal engine operation either as background leakage or during transient power or air supply changes [1-8]. In addition, engine oil smoke or fumes can be generated in the engine or APU and contaminate the compressor bleed air supply, due to failure conditions such as failed bearings/seals, improper drainage of oil into the compressor or oil reservoir over-servicing. Hydraulic fluid leaks due to an over-serviced reservoir or ruptured line may also be ingested into the APU or the engine compressor [7]. The hydraulic system fluid reservoir vent is connected to the bleed air system, therefore enabling hydraulic aerosols to enter the cabin air [9]. Bleed air contaminants may include a range of toxic substances, various organophosphates (OP), amines, a complex mixture of thermally degraded components, volatile organic compounds (VOC) including aldehydes and solvents, carbon monoxide, ultrafine particles and de-icing fluids.

**Fig. 1:**
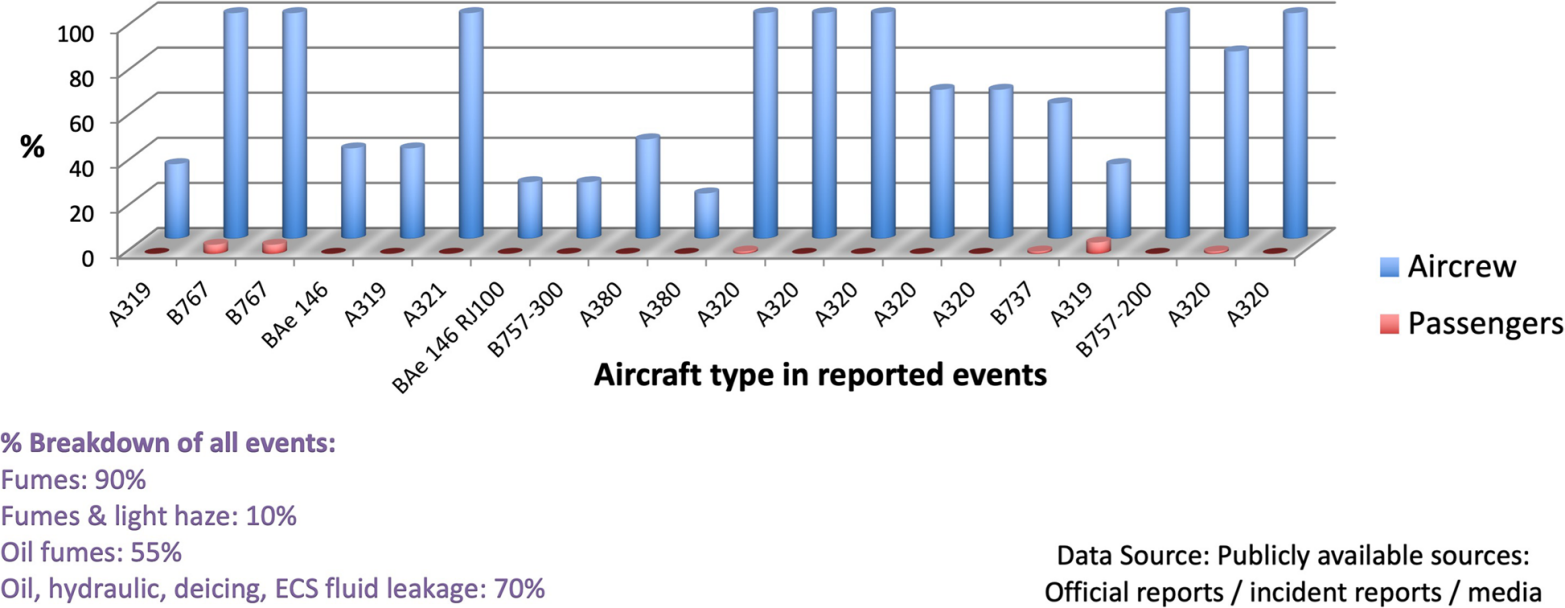
Medical attention sought or hospitalisation by aircrew/passengers after fume events (2000–2018)

Contaminated aircraft cabin air incidents, commonly identified as ‘fume events’, were first described in military aircraft in the 1950s [10-12]. The onset of fume events coincided with the introduction of synthetic jet engine oils, used in high performance turbine engines [13]. Since then, reports describing aircrew with acute symptoms, followed by chronic neurological, cardiological, respiratory symptoms and other health impacts, correlating with fume events have been published [14-34]. The term Aerotoxic Syndrome associated with exposure to air supply contaminants was first published in 2000 [35], although it is not widely understood by health care professionals. The aviation industry recognises that some people experience acute symptoms following a fume event, although debate continues as to whether exposure to contaminated cabin air can cause long-term symptoms [36-42]. A striking feature of the outcome of fume events is the difference in response observed between passengers and aircrew. While few passengers appear to suffer more than symptoms of irritation following a ‘fume event’, aircrew frequently become systemically unwell and need medical attention. Figure 1 shows this graphically from data collected from publicly available sources. This differential response suggests that the pre-exposure to thousands of hours of low-dose inhalation of engine bleed air increases the vulnerability of aircrew to acute higher dosage during a fume event.

There are no sensors on board transport aircraft and therefore no contaminants are collected at time of exposure. Cabin air monitoring studies have identified the presence of low concentrations of individual bleed air contaminants (e.g. tricresyl phosphates, carbon monoxide, formaldehyde, toluene, benzene) that are well below published chemical exposure limits during “normal” (non-incident) flights [7, 9, 38]. However, few measurements have been undertaken during documented fume events, ground-based exposure limits were not developed for application at altitude or for complex heated mixtures [43-45], and the focus has been on individual substances rather than the complex thermally degraded mixtures [2, 46]. For the assessment of symptomatic aircrew some previous efforts were made to develop guidance material for collation of medical data [47-50], however this was not recent, and it has rarely been undertaken in a timely, systematic or comprehensive manner. More recently the European Committee for Standardization has issued a technical report which recommends medical monitoring at the commencement of aircrew employment and for aircrew and passengers after fume events using a best practice medical protocol [51]. There is an urgent need for a consistent, internationally accepted medical protocol to facilitate the recognition of health effects associated with fume exposure in aircraft cabins and cockpits [26, 52]. The main objective of this paper is to compile present knowledge on causes, frequencies and clinical findings of fume events and to develop, based on this data, a recommended approach to the best reasonable and available observation, measurement and recording of symptoms and signs, and subsequent management of afflicted persons and their health outcomes. We describe a variety of diagnostic tests covering functional or organ related impacts including neurological, neurobehavioral, respiratory, and cardiological symptoms, and data on biomonitoring.

## Methods

This protocol is a consensus statement of a group of international experts on cabin air contamination and related health effects, the International Fume Events Task Force. Members (from nine countries) were selected based on clinical, professional and/or academic understanding of specific topics in the field of fume events. A literature search was conducted limited to human subjects and articles in the English language; publications were searched on causes, frequencies, and clinical findings related to aircraft fume events. MEDLINE-Database was searched with PubMed (all fields) from its inception up to 15^th^ June 2022 with the terms *aerotoxic syndrome* and *fume events.* Further resources included reference lists of other reviews or research results based on relevant objectives and collections of the authors.

Based on these data, we developed what we consider to be the most reasonable protocol for observation, measurement and recording of symptoms, signs and treatment (if any), and subsequent management of afflicted persons and their health outcomes. This protocol was conducted by various working groups, and in online consensus meetings with the expert panel, and is limited to literature specific to oil and hydraulic fluids because of the more serious health impacts rather than other types of fumes, including electrical, fan failures, exhaust, deicing fluid, and ozone [39]. This medical protocol is an expert-based work-in-progress that will be updated as the field progresses. It is not the intention of this protocol to address the issue of routine monitoring of aircraft cabin air for the presence of toxic fumes.

## Results

### Chemical constituents in engine oil and hydraulic fluid

The oil and hydraulic fumes consist of a range of hazardous substances; VOCs identified in cabin air quality studies, where quantified, ranged from 100 - 300 + compounds [9, 38, 39, 53]. More than 100 VOC compounds were identified in a bleed air investigation after oil fumes partially incapacitated two pilots [25, 54-56].

Organophosphates are utilised as an anti-wear additive at about 3% in synthetic engine oils. Tricresyl phosphate (TCP) is the most studied OP with proven neurotoxicity [46, 57, 58] and is used in most engine oils [59]. While the focus has been on the ortho isomer tri-ortho-cresyl phosphate (T*o*CP), the other ortho isomers, (mono- and di-ortho-) are at far higher levels (M*o*CP—3070 ppm; D*o*CP – 6 ppm; T*o*CP—0.005 ppm) in the oils and are 10 and 5 times more toxic [57, 60-62]. TCP used in aviation oils comprises a complex mix of cresols, xylenols and phenols, rather than the ten regularly cited TCP isomers only [57, 58, 61, 63]. Oil exposed to high temperatures was shown to alter the composition of the fresh oil, with higher rates of alkylated cresol phosphates and xylenyl phosphates formed [63], with similar toxicity to the ortho TCP isomers [46, 57, 58, 61, 63, 64]. The toxicity of the ortho isomers of TCP is significantly underestimated [62]. Some studies have reported that the non ortho (meta and para) isomers of TCP, which make up around 99.7% of the TCP blend used, also have neurotoxic properties [26, 57, 65-68]. The ortho isomers of TCP make up 0.3% of the commercial formulation of TCP used in engine oils [61], or up to 0.01% in the oil.

Hydraulic fluids typically contain a mixture of as much as 95% organophosphates, typically dominated by tributyl phosphate (TBP) (20–80%), and phenol isopropyl phosphate (PIP, 3:1), ranging from a low of 2.5% (oil) to a high of 15%, but no TCPs.

Amines such as N-phenyl-1 napthylamine (PAN) are utilised in the synthetic oils as an antioxidant at around 1%. PAN is a skin and respiratory sensitiser. Beta-naphthylamine (BNA) and N-2-naphthylaniline (PBN), are reported as low-level contaminants of PAN.

#### Thermally degraded complex mixture

When oils are exposed to high temperatures in the engines or APU, a complex mixture of thermally degraded (thermolysis) components is produced. This includes a wide range of volatile and semi-volatile organic compounds (SVOC) [7, 9, 38-40, 53, 59, 69-72]. Oil thermal degradation studies identify an excess of 127 VOCs and SVOCs and many hundreds of additional VOC peaks [73].

Ultrafine Particles are generated when the oils are exposed to high temperatures in the engine or APU [70, 74-76]. Oil contamination of the bleed air results in large increases in UFPs with peak concentrations in the 40–80 nm range [74]. The UFPs in the bleed air may be a "hint" of oil leaks [38]. Hydraulic fluid contamination is associated with increases in fine particle concentrations in the 200–1000 nm range [74].

#### Carboxylic acids

The base stock of the oils is synthesised from esters and carboxylic acids. These acids have a smell characteristic of dirty socks [55, 72].

#### CO

Low levels of carbon monoxide (CO) have been reported in cabin air sampling and oil thermal degradation studies [38, 71, 74, 77], showing a strong temperature effect of CO formation. Minimal CO is formed below 300^0^C but levels are considerably higher with increasing temperature [73, 78].

### Air monitoring findings

A number of bleed and cabin air monitoring studies have been undertaken over the last few decades, with overviews available for review [7, 9, 25, 71, 79-81]. Measurements include various organophosphates (TCP, TBP, triphenyl phosphate (TPP), trixylyl phosphate (TXP) and dibutylphenylphosphate (DBPP) and volatile organic compounds (toluene, benzene, formaldehyde, acetaldehyde, valeric acid, hexanoic acid, n-hexane). Virtually all levels identified were below available exposure limits [9, 38, 39, 51, 77, 82], however most were not undertaken during recognised fume events.

Mixed isomers of TCP were commonly identified at low levels in air sampling studies ranging from 23–100% of flights [37, 77, 82]. Traces of meta- and para- TCP isomers were identified in nearly all samples [38, 39]. Higher concentrations of TCP isomers were recorded during taxi out, take-off, climb, descent and landing, compared to the cruise phase of flight [37, 38]. TBP was identified in 73% of all flights in one study [77], and in 100% of samples in another study [38].

UFP measurements in the cabin air identified increased concentrations of UFPs, associated with engine and APU power and air supply changes in phases of flight, and correlating with times when oil seals are less effective enabling oil leakage to occur [76]. Oil contamination of the compressor results in very fine droplets in the bleed air under most operating conditions [75] with increases of UFPs during changes of power and air supply changes [69, 83]. Maximum UFP concentrations under these conditions have been identified at up to 60,000—> 500,000 particles / cm^3^ in normal flight [76, 77, 83]. UFP concentrations were identified in an EU study between a baseline of 2 × 10^5^ to a peak of 2.8 × 10^6^ particles / cm^3^ during a confirmed oil fume/smell event, significantly higher than average UFP concentrations (5000 particles / cm^3^) in indoor offices [40]. Simulated oil leakage studies identified UFPs at least 2 orders of magnitude above background levels [74, 75].

### Frequency of fume events

As aircraft have no contaminated air detection systems installed, there have been many attempts to assess how often ‘fume events’ occur. Fume events typically happen when recognisable engine oil or hydraulic fumes contaminate the bleed air stream that supplies the cabin and/or flight deck ventilation system. However, it is not possible to provide an accurate rate of occurrence, due to the three distinct ways in which exposures occur [2, 3, 84]:

- 1. Chronic repeated low dose exposure of aircrew to a complex mixture of oil decomposition products contaminating the ‘bleed air’ supply in normal operations. This is due to current engine/APU air supply designs. These will not be detected or reported.
- 2. Acute contamination events triggering a detectable odour known as a ‘fume event’, although if the rate of contamination increases gradually, this may not trigger an olfactory response.
- 3. Far less frequently a visible haze or smoke.

Fume events can range from a brief familiar occurrence in normal aircraft operations, such as during transient air supply and power changes, to the far less frequent, failure or mechanical events, of which only the latter are more commonly reported.

Other sources of fumes/smoke that can be supplied to the aircraft cabin and flight deck include electrical faults, fan failures, deicing fluid, exhaust, and galley equipment [85, 86]. However, oil and hydraulic fluid are the second most commonly reported type of fumes/smoke that are documented by US airlines, with only electrical fumes being more prevalent [85].

Low level oil leakage is well recognised [1-8, 38, 85, 87-91]. The oil leakage due to engine operating conditions *“pollutes the cabin/cockpit air”* [92]. The frequency of exposure should be viewed in terms of the design factor enabling low level oil leakage to occur in normal flight operation, rather than failure conditions only [5, 6]. The average number of acute events reported per day on the US fleet via official US databases is 5.3 events [93]. However, the lack of training of aircrew regarding fume events has led to significant under-reporting [25, 49, 84, 85]. Contamination events are widely distributed across almost all common aircraft models.

### Biomonitoring

The metabolism and disposition of TCP was studied in animal experiments. Following oral administration, tri-para-cresyl phosphate (T*p*CP) was absorbed from the intestine, distributed to the fatty tissues, and moderately metabolised to a variety of products of oxidation and dearylation of TCP, which were then excreted in the urine, faeces, bile and expired air [94]. However, in biomonitoring a considerable focus has been placed on T*o*CP exposure, its toxic metabolite (cresyl saligenin phosphate- CBDP) and its effect on acetyl cholinesterase (AChE) and butyrylcholinesterase (BChE) inhibition [95-98]. T*o*CP is difficult to detect in blood due to rapid metabolism, but there is an assay for T*o*CP, although not generally available, based on the active site serine of BChE reacting with the active metabolite of T*o*CP, cresyl saligenic phosphate [96, 99]. This cresyl phosphate-modified butyrylcholinesterase is a very sensitive biomarker in human plasma for fume event associated T*o*CP exposure [96, 99], much more sensitive than measurement of acetylcholinesterase. Six of twelve jet airline passengers tested positive for T*o*CP, without showing toxic symptoms [96].

T*o*CP is the least present of the three ortho isomers found in aviation blends of TCP, which make up collectively 0.01% of the oil [58, 61, 62]. There is over 600,000 times more mono-ortho-cresyl phosphate (M*o*CP) than T*o*CP and 1200 times more di-ortho-cresyl phosphate (D*o*CP) [62].

An analysis of 332 aircrew urine samples did not detect T*o*CP (D*o*CP) metabolites, and only one sample contained very low levels of the meta- and para- TCP isomer metabolites, (D*m*CP and D*p*CP) [100]. Metabolites of TBP (DBP) and TPP (DPP) were significantly higher in all urine samples, than in unexposed persons from the general population [100]. This was reported to be due to the release of traces of hydraulic fluids into the cabin air or flame retardants used in the aircraft cabin.

Blood may be taken to assess cholinesterase and where possible neuropathy target esterase (NTE) levels, taking into account the clinical presentation of the person and cost. Cholinesterase inhibition can be assessed in two ways, enzyme activity or mass spectroscopy. At present, the enzyme activity method is the only one that can be readily assessed, while the mass spectroscopy method must be stored locally until the University of Washington mass spectroscopy assessments can be undertaken [66, 101], see Table 1.

**Table 1:** Blood sampling for acetylcholinesterase (AChE), butyrylcholinesterase (BChE) and neuropathy target esterase (NTE)

Method	Enzyme	Half-life (days)	Sample 1 (Time after incident)	Sample 2 (Baseline) (Time after incident)	Sample details
**Enzyme assay**	AChE—Red blood cell (RBC)	33	Preferably 4 – 48 h	2–3 months ^a^	Standard protocol^g^
	BChE—Plasma	12	Preferably 4 – 24 h	1–2 months ^a^	Standard protocol^g^
	NTE^e^ (lymphocytic)	5–7		2–3 months ^a^	Standard protocol^e^ – Only fresh blood can be analysed ^c^
**Mass spec analysis**^f^	Cresylphosphate - modified butyrylcholinesterase				Plasma should be stored till a routine measurement is available
	AChE	33	4 h – 2 weeks	One sample required only ^b^	4 × 6 ml in EDTA tubes ^d^
	BChE	12	4 h – 1 week	One sample required only^b^	4 × 6 ml in EDTA tubes ^d^

^a^A second sample to be undertaken as a baseline. AChE recovers to normal level after around two to three months, while BChE recovers after around one to two months. If symptoms alleviate before this time, undertake a baseline sample before returning to work or when away from further exposures. It is preferable to undertake a baseline before starting flying employment

^b^Store locally at present

^c^NTE analysis: Only fresh blood can be used

^d^Separate (centrifuge) plasma and red cells and store separately at -20^O^C to –80 °C locally. Assays preferably tested in triplicate within the same laboratory with 5% range

^e^NTE blood analysis is not routinely available

^f^Still research study at this time. Assessing the percentage modification of AChE, BChE or other esterases using mass spectrometry

^g^Baseline AChE and BChE values for OP exposures have been generally determined for very high agricultural exposures, but not for aircraft fume event exposures and may not be sensitive enough for the latter. However, we measure them to get more data and experience about its diagnostic value. Note that there is a wide variation between individual baseline levels and therefore it is the 30–70% inhibition below the individual baseline that is the important reference. Each laboratory will use differing reference levels. Reference levels do not take into account the individual variation, which is the most important factor when analysing biomarkers as an indicator of OP exposure, as outlined above

The AChE bound to the erythrocytes (red blood cells) correlates with the AChE activity in the neurones. Reduction of AChE activity in isolated erythrocytes may be between 30 to 70% of the individual reference value (baseline). After reaction with OPs the esterase activity mainly recovers within a period of several weeks after new synthesis. Measurement of red cell AChE and plasma BChE activity can be undertaken by standard activity assays or using a ChE Check Mobile Test Kit [102]. Initial blood samples are best taken preferably between 4–24 h following the fume event for BChE, and 4–48 h for AChE. A second sample is required for baseline activity levels. Measurable AChE / BChE inhibition occurs only at higher level OP intoxications, which are not expected in fume events. Lack of inhibition does not mean OP exposure did not take place.

The neuropathy target esterase (NTE) activity in the nervous tissue is correlated with that in lymphocytes. Research based on animal studies suggests that the irreversible inhibition of NTE in the nervous tissue may be the first indicator of the onset of organophosphate-induced delayed peripheral neuropathy (OPIDN). AChE and NTE are different enzymes that serve as biomarkers of intoxication by many OP compounds. Reference values for the NTE activities were 3.01–24.0 nmol phenyl valerate / (min/mg protein) [103].

Heutelbeck et al. reported that AChE activity levels related to OP exposures were unaffected in eleven subjects within five days after fume events, while NTE activities were clustered at low levels, suggesting inhibition of NTE activities [21]. Reduced BChE levels were recorded after a fume event [19].

Urine samples could be taken to assess for specific OPs or their metabolites, and blood samples could be used to assess for VOCs (Table 2). At present, the sampling methods for the OPs and VOCs are very specific, costly and require organisation with specialist laboratories in advance to ensure the process is undertaken appropriately. In this protocol, the data will be presented for completeness, but it is unlikely that this testing can be widely undertaken at present.

**Table 2:** OPs, VOCs – Blood/urine (Non routinely available)

		Sample 1	Further samples	Sample details	
Blood	VOCs	As soon as possible after event	If possible 6 and 12 h later and 1 month later^a^	5 ml normal EDTA (2 ml transferred asap to coated headspace tubes)^b^	
Urine	OPs	As soon as possible after event	If possible, 6 and 12 h and 1 month later ^a^	20 ml	
What OPs, VOCs to look for:					
OPs	Tricresyl phosphate, meta, para, ortho isomers				Oil
	trixylyl phosphates				Oil
	tributyl phosphate				Hydraulic
	triphenyl phosphate				Oil^c^, Hydraulic
	Others: dibutyl phenyl phosphate (DBPP); triisobutyl phosphate (TiBP); 2,6-di-tert-butyl-p-cresol (BHT)				Hydraulic
	isopropylated phenyl, phosphate (3:1) (TIPP/PIP (3:1)				Hydraulic, Oil^c^
VOCs	Aldehydes, aliphatics, aromatics, ketones, alcohols and organics such as n-heptane, n-hexane, benzene, toluene, formaldehyde, acetaldehyde, n-pentane and n-octane. (Valeric acid / pentanoic acid, heptanoic acid, octanoic acid)				ASHRAE-Guideline 2021—Table 8–1 [55]
Table listing known OP metabolites					
Substance					References
T*m*CP	dicresyl phosphate (DmCP)				Schindler et al. 2013 [100]
T*p*CP	dicresyl phosphate (DpCP); p -hydroxybenzoic acid; di-p-cresyl phosphate (DCP); p-cresyl p-carboxyphenylphosphate (1coDCP)				Schindler et al. 2013 [100], Kurebayashi et al. 1985 [94]
T*o*CP^d^	dicresyl phosphate (DoCP) o-Cresyl dihydrogen phosphate and Di-o-Cresyl hydrogen phosphate, salicylic acid, o-hydroxybenzyl alcohol and o-cresol				Schindler et al. 2013 [100], Abou-donia et al. 1990 [104], Somkuti et al. 1990 [105]
TBP	DBP				Schindler et al. 2013 [100]
TPP	DPP				Schindler et al. 2013 [100]

• See supplement- appendix 7 for further advantages & limitations of biomonitoring

^a^Last sample to be undertaken 1 month later as a baseline. VOCs mainly recover to normal levels within hours and OPs probably within two or three days. If symptoms alleviate before this time, undertake baseline sample with at least one week away from the flying environment

^b^2ml blood samples have to be transferred ASAP to coated headspace tubes for Gas Chromatography-Mass spectrometry (GCMS) analysis. Contact a specialized laboratory certified for the required analysis

^c^This organophosphate is currently used at low levels in one type of 'TCP free' engine oil

^d^ToCP present in engine lubricants is at very low levels and far lower than the more toxic ortho-TCP isomers, Mono-ortho cresyl phosphate and di-ortho cresyl phosphate (MoCP and DoCP) [57, 58, 60-62]

### Clinical effects described by affected persons after an aircraft fume event

#### General

Aerotoxic Syndrome encompasses a constellation of symptoms and health disorders [3, 15-23, 26-35, 48, 81, 106-123], and as in many medical conditions with the ‘syndrome’ label, the complete list of symptoms and clinical findings is not necessarily found in any individual case. It is clear that there is considerable individual susceptibility [26]. Symptoms depend on the intensity and duration of exposure, exposure conditions, repeated exposures over time versus a single exposure and the duration of the individual’s service in the industry. Clinical factors such as diet, smoking and alcohol use, age, co-morbidities, concurrent medication, genetically impaired enzyme detoxification and reproductive status also play a role. The total accumulated dose over time is a key factor. Symptoms may be prompted by a single high exposure, repeated or prolonged low-level exposures.

#### Symptoms and diagnoses

Initial presenting complaints (see Table 3) are commonly and consistently described as foggy thinking, dizziness, recognising an odour in the cabin (commonly described as a ‘dirty socks’ smell), impaired short-term memory and cognitive thinking, fatigue, headache, nausea, tremor, balance impairment, incoordination, breathing difficulties, chest pain, cough, eye, nose, sinus and throat irritation.

**Table 3:** Acute and chronic symptoms of aircrew exposed to aircraft contaminated air—information leaflet

• Following exposure to aircraft contaminated air, whether on a chronic low-level basis or a short transient or longer duration fume event, aircrew and passengers have reported the following acute and chronic longer term adverse effects • If aircrew or passengers experience the symptoms outlined below at the time of exposure or fume event or soon after, it is advisable to report this to your medical professionals	
**Acute signs and symptoms**	**Chronic symptoms**
**Irritation / burning**—eyes, nose, upper airways, skin; blisters/rash on uncovered areas	Irritation / burning—eyes, nose, upper airways, skin, blisters / rash
**Neurology**: Headache/head pressure; impaired / loss of consciousness; dizziness / light-headedness; drowsiness / lethargy; confusion/ disorientation / intoxication; balance problems; vertigo; vision problems; nystagmus; shaking / tremor; gait problems; erratic movement; impaired speech; impaired memory / slowed mental processing / concentration; difficulty writing; paraesthesiae / numbness; sweating / loss of temperature control, pallor / flushing / altered taste / sleep disturbance; anxiety	Headache / head pressure; dizziness / light-headedness; lethargy; vision problems; slowed mental processing / impaired memory and concentration / difficulty multi-tasking / slowed mental processing; balance problems; tremor / gait problems; incoordination; paraesthesia/numbness; sweating / loss of temperature control/pallor / flushing / taste; behavioural / personality changes – unreality / anxiety / depression; sleep disorders / PTSD
**Gastrointestinal:** Nausea / vomiting / diarrhoea	Nausea / vomiting / diarrhoea
**Respiratory**: Breathing difficulties / shortness of breath / cough / chest tightness / wheezing / lung irritation	Breathing difficulties / shortness of breath / cough / chest tightness / wheezing / lung irritation; susceptibility to upper respiratory tract infections
**Cardiological**: Increased heart rate / palpitations / chest pain / tightness	Variable heart rate / palpitations / chest pain / tightness
**General:** fatigue; Joint / muscle pain; twitches; weakness	Fatigue; chemical sensitivity; joint / muscle pain; twitches; weakness; vocal / nasal / throat – polyps / swelling; hair loss

Adapted from [26, 31, 33, 48]

Long-term intractable cough, breathing difficulties, central and peripheral nervous system complaints are the common features of those affected by a fume event. In many cases, symptoms are of short duration, but in others may take many hours, days or weeks to resolve. In some cases, particularly those who have experienced more than one fume event, symptoms can continue for months or years and, occasionally, full recovery never occurs. Most individuals report the onset of symptoms to be time correlated with a flight or immediately afterwards, in one of the following timeframes:

- In-flight (ground or air).
- Immediate post-flight (within one to two days).
- Late/subsequent (beyond two days).

A variety of diagnoses have been applied to fume event affected individuals including chronic bronchitis, nasal pathology and sinusitis, vocal cord polyps, irritant induced asthma, interstitial lung disease, cognitive dysfunction, and toxic encephalopathy [26]. Multiple chemical sensitivity is sometimes diagnosed [20, 23, 25, 26, 29-32]. People suffering from the effects of aircraft fume events are commonly misdiagnosed as being anxious, stressed or experiencing other clinical complaints [20, 23-26, 36, 37, 124, 125]. Misdiagnosis occurs because the toxic effects of fume event-associated exposure to various noxae, including thermally degraded aircraft engine oil, are not widely recognised by health care professionals.

### Respiratory / cardiac complaints

The respiratory tract is the common portal of entry for cabin air contaminants, although entry through the skin and alimentary tract is also recognised. As it receives the total cardiac output it is systemically more exposed than other organ systems thus, theoretically at least, increasing possible toxicity [126].

Respiratory complaints consistent with lung injury among aircrew are common [2, 14, 17, 19-27, 29-34, 48, 106-108, 112, 114-116, 118, 122, 124]. Granulomata consistent with sarcoidosis has been occasionally reported in pilots and military veterans [116, 127]. A European Commission funded study reported that exposure to engine oil and hydraulic fluid fumes can induce considerable lung toxicity [128].

Cardiac involvement is suggested by irregular heart rate, fatigue, cough, breathlessness, cyanosis, flushing, and an increase in blood pressure. There are no systematic documented reviews of the effect of heart rate and blood pressure after fume events, but cardiac abnormalities have been reported by aircrew [14, 17, 19-22, 25-27, 30-33, 48, 106, 108, 112, 114, 118]. At the pathological level, myocardial and pericardial damage has been reported in acute OP poisoning. Importantly, ECG and echocardiography may be normal.

### Neurological complaints

The brain is a recognised OP target organ for toxicity, with the central nervous system (CNS) being particularly vulnerable to toxic insult. A major reason why the brain is a susceptible target organ is that nerve cells must last for a lifetime and are terminally differentiated and cannot, as with most other tissues, repair by cell proliferation [51, 129]. Central nervous system (CNS) effects, reported after exposure to fume events include general incapacity, temporary paralysis, impaired or loss of consciousness, headache, nausea, dysarthria, balance problems, ataxia, cognitive impairment, tunnel or double vision, dilated pupils, nystagmus, and sleep problems, with early symptoms being flu-like. Symptoms of exposure to fume events may also involve the peripheral nervous system (PNS), with motor, sensor and autonomic reactions such as: sweating, loss of temperature control, pallor, flushing and altered taste, tremors, incoordination, muscle weakness, paraesthesia, numbness in limbs and other areas, consistent with peripheral neuropathy.

Although standard neurological testing has often reported negative findings, neurological abnormalities in crew related to fume events have been regularly reported [14, 16, 17, 19-27, 30-34, 48, 81, 106-108, 112, 114, 117, 118, 122, 130-132]. In the case of aircrew, chronic pre-exposure is assumed [2, 3, 26]. Aircrew with a longer flying history appear to be more susceptible than those who have been employed for shorter periods, suggesting a cumulative effect [2, 3, 18, 19, 22, 25, 26, 31]. Repeated low dose exposure to OPs on neuronal cells increase the susceptibility to neurotoxic damage upon further higher dose exposure [26, 133]. The neurological pattern of symptoms reported appear to onset during the flying career and show a temporal relationship with time spent on board aircraft as they onset or worsen when flying and reduce or resolve during days off.

### Mechanisms of OP-toxicity

Many of the symptoms (especially the neurobehavioral symptoms) that have been associated with “aerotoxic syndrome” have been documented in agricultural workers, sheep farmers (sheep dipping) and pesticide sprayers as well as veterans of the United States, and other countries who served in the 1990–1991 Persian Gulf War. In these scenarios, organophosphate exposure (i.e. as pesticides or nerve agents) has been discussed as a plausible explanation for the chronic neurologically based symptoms [2, 3, 134-139].

The symptoms associated with the toxicity of OPs involves three main categories [139, 140]:

1. The primary action of OPs at very high doses is the irreversible inhibition of acetylcholinesterase (AChE), resulting in accumulation of acetylcholine and overstimulation of the nicotinic and muscarinic AChE receptors with cholinergic effects. OPs inactivate cholinesterases by attaching an alkyl phosphate group to the hydroxyl group of a serine residue at the enzyme's active site. Recovery from such inhibition generally takes 10–14 days [141]. Cholinergic symptoms depend on the OP compound, dose, frequency, duration and the route of exposure, combined exposure to other chemicals and individual sensitivity and susceptibility [104, 139, 140]. Initial symptoms of *mild* toxicity include fatigue, dizziness and sweating, sometimes accompanied by headache, inability to concentrate, cognitive dysfunction, weakness, anxiety, tongue and eyelid tremors, miosis and tightness of the chest [140]. If moderate to severe OP toxicity ensues, the so-called “cholinergic crisis” or “cholinergic syndrome” develops, which includes more elevated levels of sweating and salivation, profound bronchial secretion, bronchoconstriction, diarrhoea, muscle tremors and fasciculations, and worsening CNS effects (e.g. dizziness, inhibition of central respiratory centres, convulsions, coma). Death can occur as a result of respiratory failure [142]. Symptoms of *moderate* and *severe* poisoning are not consistent with symptoms reported by aircrew, these are high-dose health effects.
2. Organophosphorus ester-induced delayed neurotoxicity (OPIDN) is a central and peripheral axonopathy with the early stage characterised by peripheral effects that recover as peripheral nerves regenerate. The later stage is central, which is more permanent. OPIDN may be caused by single or repeated exposure and is accompanied by a Wallerian type (or dying back) axonal degeneration and secondary demyelination in the most distal portion of the longest tracts in both the central and peripheral nervous system [139]. The clinical picture is manifested by mild sensory disturbances, ataxia, muscle fatigue and twitching, and improvement may require months or years. The neurotoxic effects of tri-ortho-cresyl phosphate (T*o*CP) and of exposure to other OPs have long been recognised as OPIDN, however the clinical picture being observed does not fit this pattern. Nevertheless, industry risk assessment studies have focused almost entirely on OPIDN as the toxicological endpoint, suggesting the levels of T*o*CP are too low to cause OPIDN [37, 39, 42, 57, 62, 125, 142, 143].
3. Organophosphorus-induced chronic neurotoxicity (OPICN) is associated with exposure to large acutely toxic or small subclinical doses of OP compounds. Excessive cholinergic activity produces delayed neurodegeneration in various brain areas (cortex, cerebellum, hypothalamus, amygdala) and in the spinal cord, that could explain persistent neuropsychiatric, neurologic and behavioural problems [139]. Clinically, OPICN is manifested by headaches, dizziness, anxiety, apathy, restlessness, labile emotions, anorexia, insomnia, fatigue, inability to concentrate, memory deficits, depression, irritability, confusion, generalised weakness, tremors, respiratory, circulatory and skin problems, with not all people exhibiting all these symptoms [139, 140]. Reports on OPICN occurring in individuals, following long-term, subclinical exposures without previous acute poisoning, have been inconsistent, partially due to the difficulty in defining exposure levels [139]. Symptoms may persist for years after exposure and are distinct from cholinergic and OPIDN effects [139].
4. We suggest a fourth category: non-cholinergic mechanism of OP toxicity, after chronic repeated low-dose exposures. The clinical picture in aircrew is not like nerve gas poisoning and does not match classical OPIDN, although there are clearly features in common. The neurological pattern of symptoms constitute a group of non-localising functional deficits which are consistent with a diffuse toxic encephalopathy as described in Michaelis et al. [26]. The pattern is in many ways directly comparable with the symptoms suffered by farmers from ‘dipper’s flu’ [134, 135]. Chronic repeated low-dose exposure to OPs, the norm with air crew, may have effects at exposure levels below those required to cause lowering of acetylcholinesterase, including oxidative stress, and neuro-inflammation, combined with effects on the known OP targets: motor proteins, neuronal cytoskeleton, axonal transport, neurotrophins and mitochondria [136, 137]. Axonal transport is crucial to maintain brain structures in a healthy state by delivering a number of substances and structures, to and from the neuron cell body. This interferes with the delivery of transmitter substances, neurotrophins and function of mitochondria, and could be the basis for the development of a diffuse subacute encephalopathy [2, 26].

In addition to the direct and delayed OP-induced neurotoxicity effects, concern exists about the potential long-term risks of neurodegenerative disease in aircrew exposed to cumulative low-dose OPs. There is a correlation between exposure to OPs and development of neurodegenerative diseases including Parkinson’s, amyotrophic lateral sclerosis/motor neurone disease and Alzheimer’s disease [136, 139, 144-146]. Cohort studies of aircrew report increased disease rates for motor neurone disease and a twice as high mortality rate of amyotrophic lateral sclerosis (ALS, the most common form of progressive motor neurone disease), when compared to the general population [25, 119, 147, 148].

### Neuroimaging

Positron Emission Tomography (PET) was performed in 26 flight attendants, presenting with "neurotoxic complaints" after exposure to contaminated air, associated with fumes from the APU on one or more occasions. PET was abnormal in 12 of these 26 aircrew, with decreased activity in the frontal regions and increased function in occipital areas and the limbic system, consistent with toxic encephalopathy [117].

Diffusion Tensor Imaging (DTI) MRI identified small clusters in the brain in which white matter microstructure was affected in aircrew reporting cognitive impairment and depressive symptoms [28]. Higher cerebral perfusion values in the left occipital cortex and reduced brain activation on an executive function task was observed and cognitive impairment was associated with white matter integrity. Defects in brain white matter microstructure and cerebral perfusion are potential neurobiological substrates for cognitive impairments and mood deficits reported in aircrew [28].

### Neurobehavioural and neuropsychological effects

Reported neurobehavioural and neuropsychological health effects associated with fume events include disorientation, dizziness, confusion, lethargy, altered behaviour, personality changes, anxiety, depression, difficulties with problem solving, concentration, memory and writing, and euphoria [14, 16-27, 31-34, 48, 106-109, 111, 112, 114, 117, 118, 122, 131, 132]. In a survey of international aircrew (50 pilots, 970 cabin crew) to establish how many believed they were suffering occupational health problems as a consequence of their working environment, including fume events, 23% reported no time off sick and only 12 aircrew members reported no time off sick and no symptoms. Forty-five percent of the respondents reported confusion and difficulty in thinking, 55% had difficulty concentrating, and 49% had memory loss [114]. Neurobehavioural symptoms were reported as the highest category in 64% of pilots with chronic ill health in a survey of British Aerospace 146 (BAe 146) pilots exposed to acute and chronic oil fumes [26]. Long-term cognitive dysfunction was identified in four cases involving aircrew exposed to oil and hydraulic fumes in fifteen incidents [26]. Slowed information processing speeds, slowed reaction times and executive dysfunctions were identified in pilots and flight attendants [18, 22, 109, 111, 132]. In these studies, the pilots' neuropsychological profile mirrored that seen in other neurotoxic conditions, such as sheep dipper's flu [138].

### Peripheral nervous system

In surveys of health symptoms in aircrew, sensory complaints such as paraesthesia, tingling, restless legs, muscular jerking, and numbness are reported in 20–77% of cases [17, 24, 26, 30, 32, 107], consistent with a toxic sensory polyneuropathy. In some of these studies respondents reported that symptoms occurred after exposure to oil or hydraulic fluid leaks and fumes from the aircraft ventilation system and subsequently sought medical attention [17, 32]. In other studies, a temporal relation between the onset of symptoms and exposure to fume events was reported [30]. 93 of 106 pilots (88%) reported that they had been involved in at least one fume event [24]. In a larger study of 274 pilots, 88% were aware of exposure to aircraft contaminated air associated with oil fumes, 34% reported frequent fume event exposures and 18% one or two big events [26]. PNS symptoms were reported in 11 of 15 incidents of which 87% involved maintenance confirmation of oil leakage [26]. Winder and Balouet described sensory symptoms (tingling, numbness) in five of seven pilots [31]. Paraesthesia/tingling feelings of the hands were also reported in almost 30% of 34 flight crew members in one study [17], and in 13 of 38 cases (34%) in another study [30].

Symptoms of small fibre neuropathy, a subtype of polyneuropathy of thin myelinated and unmyelinated nerve fibres, are painful burning paraesthesia and hypersensitivity to touch and temperature changes. There is one study on the prevalence of small fibre neuropathy in aircrew reporting adverse effects associated with fume events [118]. In nearly all patients in this study group, neuropathological investigation by skin biopsy showed that the intra-epidermal nerve fibre density was significantly decreased.

### Other findings

Persons exposed to fume events may also present with a variety of other symptoms and clinical findings. Irritation to the respiratory tract, eyes and skin are commonly reported in association with fume events [2, 14, 17, 19, 20, 22-27, 29-34, 48, 106-108, 112-114, 122]. Many of the substances are recognised as skin and respiratory sensitisers, associated with allergy and asthma symptoms and breathing difficulties [20, 24-27, 29-33, 107, 112, 114, 115, 149-151]. Gastrointestinal symptoms such as nausea, vomiting and cramps are regularly reported by aircrew after fume events [14, 16, 17, 19-27, 29-34, 48, 106-108, 114, 118, 122]. Liver function abnormalities are associated with prolonged or repeated exposure to various substances in the fluids [149, 150, 152, 153].

Chemical sensitivity is often reported associated with fume events [17, 20, 23-27, 29-33, 107, 113, 114]. Some of the substances are classified as suspected of causing harm to fertility or the unborn child or toxic for reproduction [26, 32, 113, 114, 149, 150]. Several contaminants are listed as a human carcinogen (BNA) or suspected carcinogens including, TBP, and PBN [26, 149, 150, 152, 153]. In a study of 5,366 flight attendants in the 2014–2015 Harvard Flight Attendant Health study, a 34%-66% increase in female reproductive cancers including breast cancer is reported, compared to 2,729 controls [112, 113]. Higher rates of cancer in aircrew were reported in aircrew surveys [114], including glioblastoma multiforme (GBM) brain tumours [25, 26]. Many risk factors have been examined as potential contributors to glioma risk, including ionizing radiation, circadian desynchronosis, heritable risk alleles and mobile phones. While these studies [25, 26] identified higher rates of exposure to oil fumes via the aircraft air supply [15, 25, 26, 36, 154], supporting that there could be an occupational link, further epidemiological investigation is warranted.

Sleep disturbances [25, 26, 31, 32, 48, 112, 113], fatigue [17, 19-27, 29-34, 48, 107, 112, 114], changes in visual acuity and eye disorders [14, 16, 17, 20, 21, 24-27, 29-32, 107, 113, 114, 122, 155] and joint pains have often been reported [17, 19, 20, 24-27, 29-32, 48, 107, 113, 114]. The product Safety Data Sheets often list a variety of adverse effects associated with breathing oil or hydraulic fumes including: eye nose and throat irritation and *“most important symptoms and effects both acute and delayed—headache, dizziness, drowsiness, nausea and other CNS effects. Shallow respiration, low blood pressure, bluish skin color, convulsions, coma and jaundice”* [156]. Other examples include dermatitis, allergic skin reaction, damage to organs through prolonged or repeated exposure, suspected of damaging fertility [156-158].

### Emerging areas

Our understanding of the clinical, toxicological and pathological issues that underpin the various presentations of the Aerotoxic Syndrome is progressively improving, particularly over the last twenty years. The following are some of the areas where significant progress has been made or can be made in our understanding of the condition:

1. Ultrafine particles.

Studies have shown that UFPs (less than 100 nm/0.1micron) are more toxic than larger particles [159-163]. Exposure to pollution, fine particles (PM < 2.5–10) and UFPs have various adverse effects on health [164-167]. These include cardiopulmonary [162, 163, 165, 168-170], and neurological effects, including impaired cognitive performance [171, 172]. Exposure to airport and traffic related UFPs may increase the risk of brain, lung and childhood cancers [173-176]. Individual particles are capable of inducing inflammation and oxidative stress [160], suggesting that particle number concentrations, which are dominated by UFPs, may be more indicative of potential health impacts than particle mass concentrations. UFPs have a high alveolar deposition fraction and have the potential to translocate into the blood circulation system.

UFPs up to several hundred thousand particles / cm^3^ have been identified in both cabin air studies [38, 40, 69, 76, 77, 177] as well as thermally degraded oil and bleed air studies [70, 73-75]. A 2019 review on aircraft exhaust emissions found that the nanoparticles were dominated by nearly intact forms of jet engine lubrication oil [178]. This oil is exposed to temperatures up to 1700 °C in some areas of the engine during the normal lubricant duty cycle leading to more or less complete thermal degradation, with subsequent UFPs and thermally degradation product formation, of which some will enter the cabin air supply [2].

*Short term* exposures to aviation related ultrafine particles near a major airport was found to be associated with decreased lung function and a prolonged QTc interval in healthy adults [179]. With respect to respiratory irritation, jet engine particles were found to have similar toxicity to diesel exhaust emissions [180]. However, this does not apply to neurotoxicity. The *continual* presence of ultrafine particles over a typical working lifetime in air crew of up to 20,000 h will predispose them to chronic respiratory problems and will exacerbate the translocation of neurotoxic substances across the blood brain barrier [2].

2) Serum autoantibodies against brain specific proteins.

Brain reactive autoantibodies are present at low levels in the vast majority of human sera [181]. Exposure to OP compounds may lead to damage of the blood–brain barrier [17, 182]. As a result, neural proteins can leak into the blood and elicit an autoimmune response, with an increased level of autoantibodies. Testing autoantibodies against brain neuronal and glial proteins to obtain objective evidence of central nervous system injury has been useful in symptomatic commercial aircrew, and in distinguishing veterans with Gulf War disease from healthy controls [17, 182-184]. In 34 aircrew with CNS related complaints higher autoantibody levels against MAP-2, tubulin, MBP, tau and GFAP than matched controls were found [17]. In a report of a 43-year-old airline pilot suffering from many fume events, who presented with neurological deficits of OPIDN and other symptoms, analysis of the serum confirmed grossly elevated levels of serum autoantibody biomarkers for neuronal cell degeneration compared with a control group [130, 131]. The pilot died without regaining good health; his death could be explained by functional disorders of the brain and the heart in combination with intake of pentobarbital. At autopsy, brain and spinal tissues showed axonal degeneration, spongiosis and demyelination. Peripheral nerves showed T-lymphocyte infiltration and demyelination. T- lymphocytes had also infiltrated the heart muscle tissue (myocarditis). It was concluded that the work environment, clinical condition, histopathology and serum biomarkers for nervous system injury were consistent with organophosphate-induced neurotoxicity.

Unfortunately, this test of autoantibodies, performed by the University of Durham, North Carolina is not available at present. This is remarkable because these autoantibodies are studied in several clinical trials [185]. For instance, autoantibodies against tau protein can reduce pathology and functional decline in animal models of tauopathies and may be beneficial in Alzheimer's disease and Parkinson's disease. So, we hope this method of assessment of aircrew with exposure to toxic compounds will soon be available elsewhere. Procedures for determination of autoantibodies to neural proteins are outlined in the supplement annex.

3) Increased genetic susceptibility to toxic compounds.

Inter-individual variability in response to toxic substances is the norm. For example, there are defined genetic polymorphisms which influence aldehyde dehydrogenase activity, significantly affecting individual tolerance for the effects of ethyl alcohol [186].

The same is the case for organophosphate compounds, although much more complicated. Genetic variability and levels of expression of genes involved in the detoxication of organophosphorus compounds (OPs) such as jet engine anti-wear agents and hydraulic fluids contribute to the variability in sensitivity to exposures to these compounds. Inter-individual genetic variations in the ability to metabolise OPs, may explain why some aircrew and passengers develop symptoms even at low doses, whereas others undergoing the same fume event may remain asymptomatic [66, 187, 188].

The key enzymes that influence individual response to OP exposures include cytochromes P450s (especially CYP450 3A4), carboxylesterase, butyrylcholinesterase (BChE) and paraoxonase-1 (PON1). Key enzymes that protect against increased oxidative stress associated with OP exposures include glutathione S-transferases, superoxide dismutases, and PONs 1, 2, and 3 and others.

With respect to genetic variations in levels/activities of specific proteins, it is important to examine the given variations in detail. Effects measured in vitro will not necessarily translate into the same effects in vivo. A good example is the PON1 genetic variant which inactivates paraoxon (the neurotoxic metabolite of the OP insecticide parathion) via hydrolysis in a test tube. One isomer (Arginine-192; PON1_R192_) of PON1 does so at a rate approximately seven times faster than another (Glutamine-192; PON1_Q192_) [189], suggesting that the faster variant is more protective. However, neither variant protects against paraoxon exposure because the in vivo rates of inactivating paraoxon are not sufficient to protect from ill effects [190]. There is little evidence that PON1 hydrolyses the triaryl phosphates added to jet engine oil in vivo.

Some proteins protect by stoichiometric binding to OP compounds, such that higher levels are more protective. This is relevant to carboxylesterase, for example, which varies by at least 18-fold in humans [191]. Also, inter- and intra-individual variations in the levels of BChE have been defined, modulating the effects of OP exposures [192].

High levels of enzymes that modulate the oxidative stress associated with exposure to specific OPs (e.g. glutathione synthetase, glutathione transferases, PONs 1, 2 & 3) may provide some protection against exposures. The CYP450 3A4 enzyme converts several of the triaryl phosphates into metabolites that are potent inhibitors of physiologically crucial enzymes (unpublished results^1^). Genetically based diverging levels of CYP 450 3A4 indicate differences in individual hepatic activity of 40-fold [191].

In summary, inter-individual genetic differences can influence susceptibility to the ill effects of OPs in engine oils and hydraulic fluids, whether those effects follow chronic low dose repeated exposures, a higher dose “fume event,” or a combination of the two. The precise mechanisms will need to be validated through animal studies [e.g. [190, 193]] or, when possible, through cell culture model systems.

4) Low-level repeat exposure to mixtures.

Substances that are not toxic individually may become toxic within a chemical mixture of thermally degraded components. Risk assessments based upon single substance evaluations underestimate the toxicity of a mixture. This could occur through exposure to multiple chemicals that cause the same effect, or interactions between chemicals may change the dose response relationships observed for chemicals tested in isolation [194]. Combined exposure to multiple chemicals can lead to health/environmental effects even if single substances in the mixture do not exceed safe levels [195]. Low-dose exposures to mixtures of chemicals in the environment may be combining to contribute to environmental carcinogenesis [196].

5) Chronic low-level exposure to OPs.

Repeat low level exposure to OPs is distinct from acute single exposures. The acetylcholinesterase-based mechanism cannot alone account for the wide variety of adverse consequences of OP exposure that have been described, especially those associated with repeated exposures to levels that produce no overt signs of acute toxicity [137].

As an example, repeated exposures to the nerve agent DFP, at doses that are below the threshold for acute toxicity, can result in alterations in myelin structure and persistent decreases in axonal transport in the rodent brain [197]. Low-level OP exposure may lead to 1) non-cholinergic toxic effects by interference with axonal transport [136, 137], and to the generation of brain-specific autoantibodies that target proteins known to play critical roles in both myelination and axonal transport [17, 182, 183]. An “autoimmune” response might offer one explanation for why OP exposures could lead to chronic symptoms [197]. Axelrad et al. identified that repeat very low dose exposure to certain OPs on neural cells increased the susceptibility (reduced the threshold for toxicity) to neurotoxic damage upon further higher dose exposure [133]. The continual low dose exposure with occasional higher-dose episodes (‘acute on chronic’ mechanism) must therefore be considered for aircrew chronically exposed to fumes [3, 26]. It is typical among aircrew to log in excess of 20,000 h flying time in a career [2]. Over that period, a person would typically inhale 9,000 cubic metres of air (nine million litres). The constant presence of low dose exposures may have considerable cumulative effects. By looking at the various reported levels of OPs in the literature it is possible to make an estimate of their internalised dose of TCP during a working lifetime as shown in Table 4. The concentrations listed and therefore the estimated dose, will be based on the amount of vapour present. However, this is likely to be an underestimate due to recent research which has shown that much of the internalised dose will be in the form of nanosized oil droplets [62, 178].

6) Endocrine disruptors.

**Table 4:** TCP Internalised dose during aircrew working lifetime

	**Study** **A (maximum)** **B (mean)**	**Conc. µg/M**^**3**^ **(TCP)**	**Vol M**^**3**^	**Dose mg**	**Notes**
A	Cranfield (2011) [77] (Crump et al.)	37.7	9000	339	Minor fume events noted by researcher in 25% of flights. (Assumed, incorrectly, to be minor and therefore not reportable)
B	Cranfield (2011) [77]	0.22	9000	1.9	As above
A	EASA (2017) (Schuchardt et al.) [38]	1.51	9000	13.6	No fume event / oil leakage identified-T-CAC
B	EASA (2017) [38]	0.009	9000	0.081	No fume event
A	Honeywell / Malmo (1999) [54]	20.3	9000	183	Fume event—pilot incapacitation
A	Rosenberger * (2018) [82]	0.981	9000	8.8	Fume event / diversion in 1 of 17 flights
B	Rosenberger * (2018) [82]	0.065	9000	0.58	Fume event / diversion in 1 of 17 flights
A	De Ree et al. (2014) [37]	0.155	9000	1.4	No fume events
B	De Ree et al. (2014) [37]	0.0069	9000	0.062	No fume events

Dose = concentration x volume

TCP (mixed isomers) dose

^*^Rosenberger (2018) - Averaged over 17 flights

OP flame retardants may have effects on the oestrogen receptor, androgen receptor and glucocorticoid receptor [198, 199], by enhancing or blocking the activity of the naturally occurring ligand, such as oestrogen or testosterone. As an example, TCP has endocrine disrupting effects via various hormone pathways including the oestrogen receptor [198-201], which is implicated in breast cancer [202, 203].

7) Causal connection.

How can we move from an observed association to a robust causal inference? Bradford Hill’s seminal paper of 1965 identified nine features of available evidence, which, if present, could help justify a robust causal inference [204-206]. He was careful to point out that if these features of the evidence were absent, then that did *not* justify concluding that the agent being evaluated was *not* causing harm [207]. The weight of evidence is suggestive of a causal link between aircraft cabin contamination and health effects in some crew and passengers, see Table 5. We should not be surprised, as the exposure and effects are biologically entirely in keeping with the science of OPs [51, 136, 137, 197]. Further supportive causation evidence is stated in a paper by Harris and Blain: *“High doses of a toxic chemical will give rise to an acute toxic response, but prolonged exposure to low concentrations of a toxin may only cause a slowly developing chronic response. The circumstances of exposure and the toxicity of the toxin will determine which of these is the more serious”* [129]. In their paper, they quote five cardinal signs on causation in neurotoxicology propounded by Schaumburg [129, 208]:

- (1) *Presence of the suspected agent is confirmed by history and either environmental or clinical chemical analysis*. (Fugitive emissions in engine bleed air is recognised and has been demonstrated on many occasions.) – [1, 2, 4-8, 26, 36, 38, 51, 75, 87-92].
- (2) *Severity and temporal onset of the condition are commensurate with duration and level of exposure*. (Supported by clinical data). [3, 11, 16, 17, 19, 20, 22, 23, 25-27, 30, 32, 36, 48, 51, 81, 96, 131, 155].
- (3) *The condition is self-limiting and clinical improvement follows removal from exposure:* the medical condition of air crew affected by Aerotoxic Syndrome can improve after removal from the cabin environment, though usually slowly and not always completely. [11, 16, 17, 20, 23-26, 32, 33, 131].
- (4) *Clinical features display a consistent pattern of affected organs that correspond to previous cases:* there is a consistency of the presenting symptomatology [2, 17, 20, 25, 26, 32, 33, 48, 51, 81, 197].
- (5) *Development of a satisfactory corresponding experimental in vivo or in vitro model is absolute proof of causation:* there is ample experimental evidence of the toxicological damage caused by repeated low-dose exposure to OPs [51, 136, 137].

**Table 5:** The Bradford Hill approach applied to Aerotoxic Syndrome (2017)

**Strength of association:** Case studies and clinical data indicate clear health impacts in significant proportions of exposed groups.
**Consistency:** Clinical data consistent with known toxic effects of organophosphates; and across varying aircraft types / countries.
**Specificity:** Aerotoxic Syndrome is a syndrome (as is Acquired Immune Deficiency Syndrome; Multiple Chemical Sensitivity; Occupational Asthma; Gulf War Syndrome and Asperger’s Syndrome) and with common neurological/respiratory symptoms linked to oil leakage/pyrolysis products exposure in cabin air.
**Temporality:** Aerotoxic Syndrome was never reported prior to the introduction of engine bleed air pressurization systems and cabin air contamination precedes linked health effects.
**Biological gradient:** High contaminant exposure often causes greater health effects; but low dose effects also apparent, suggesting non-linearity.
**Plausibility:** The known effects of organophosphates and other cabin air contaminants support a causal link.
**Coherence**: Animal and human data support a causal link.
**Experiment:** Some health effects are reversible after exposure cessation, especially for acute exposures.
**Analogy:** Polychlorinated biphenyls; hot rubber fumes; welding fumes; traffic fumes, occupational asthma, leaded petrol, methyl mercury, organophosphate pesticides and tobacco smoke have relevant features.

Data source: [207]

### Medical protocol: The documentation of a fume event, clinical history and physical examination

**Fig. 2:**
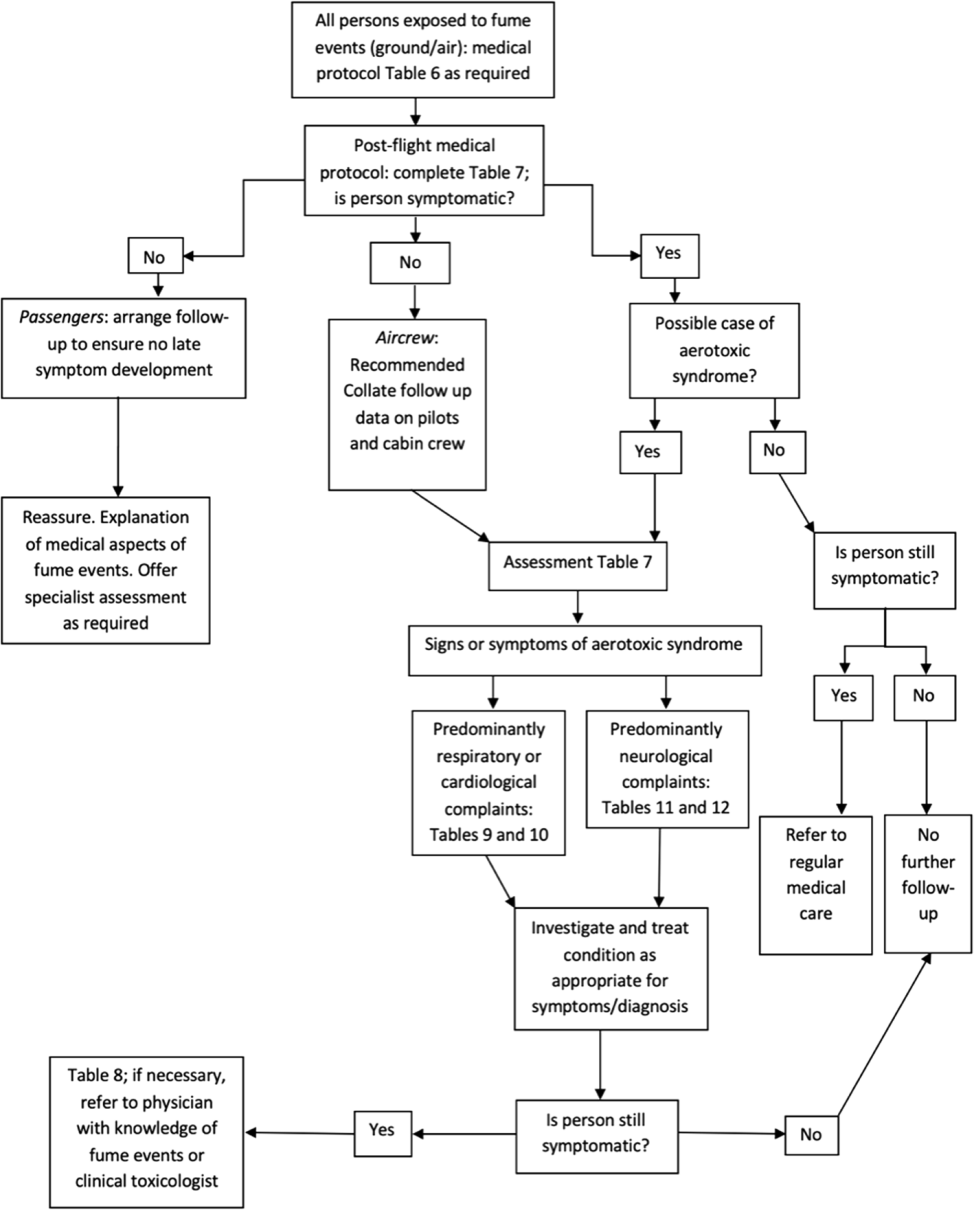
Recommended investigation and management approach for persons exposed to fumes / fume events

In all reported cases of toxicity following fume event exposures, or Aerotoxic Syndrome, the onset of symptoms may occur in-flight (ground/air) at the time of or following the fume event, immediate post-flight (within one to two days) or later/subsequently beyond two days. The recommended management approach upon presentation to a medical facility is outlined in Fig. 2. Our recommendations for data collection, medical examination and special investigations are set below and formatted to examine each organ system dysfunction individually and are divided into time of presentation: Table 6: In-flight; Table 7: Immediate post-flight; Table 8: Late/Subsequent.

**Table 6:** In-flight post event medical protocol (ground/air)

**The following recommendations are made knowing that they may involve non-medically trained persons in an aircraft cabin in-flight (ground or air)**
**Environmental observations**
• Type of aircraft.
• When did the event occur (in-flight, stage of flight, on ground, ascent, descent)?
• Where in the aircraft did the event occur?
• For how long did the event continue?
• What happened (e.g., odour, fumes, smoke)?
• If odorous fumes, describe.
• Who and how many (x out of y) was / were affected, when and for how long (aircrew, passengers)?
• Record of air quality monitor recordings (if available) / maintenance history/previous events if known.
• First aid response.
**History of symptoms and measures related to the fume event**
• A detailed and carefully documented description and severity of the fume event experienced by the individual.
• Record symptoms and progression of symptoms.
• Record observations of others, important in assessment of affected persons
• Record any treatment given / used.
• Record any treatments for past exposures, if known.
• Measure and record oximetry, if available, before oxygen administration.
• Record whether oxygen was used (including flow rate, method of administration (for example, nasal cannulae / mask, when and duration).
• Record any unusual behaviour.
• If possible, record pre-existing health complaints / disorders / findings / medication.
• Record other possible diagnoses (to be considered for differential diagnosis purposes).
**Physical examination**
Trained healthcare professionals may not be present to conduct a medical examination. However, observations of physical findings or behaviours should be recorded because they are helpful to future medical carers in their initial assessment and for guiding ongoing medical management. If a trained health care professional (e.g., a doctor, nurse or paramedic) is present, a physical examination is strongly recommended.

**Table 7:** Immediate post flight medical protocol

**Medical history of event**
A detailed and careful occupational history of the fume event, including timing, severity and duration of the fume event. Also record the frequency, duration and intensity of previous fume exposures:
• Record total flying hours (Pilots will know this from their logbooks. Cabin crew can estimate total hours from contracted annual hours x length of service less time for absences such as annual and sick leave, part-time work and maternity leave).
• Record symptoms and progression of symptoms including those observations made by other people, such as crew members and passengers (important in assessment of affected persons), any treatment given/used, whether oxygen was used and when/duration including flow rate and unusual behaviour (e.g. impaired balance, cognitive status, short term memory) as outlined in Table 5.
**Clinical examination**
• Record general appearance (for example, breathlessness, pallor, agitation).
• Measure and record respiratory and heart rate, blood pressure.
• Auscultation of heart and lungs.
• General physical examination.
• Record percutaneous oxygen saturation, record inspired oxygen concentration).
• Monitor SpO_2_, if initial SpO_2_ < 95%.
• Assess neurological status (conscious state, balance, muscle weakness, numbness, pupils, muscle reflexes, check for tingling of limbs, muscle cramps, tremor).
• Assessment using the Mini-Mental State Examination MMSE: (Orientation for time and place; attention and calculation; memory and processing speed).
• Other abnormal findings.
**General investigations**
General investigations should be undertaken as soon as possible following a fume event, but should ideally be within two to four hours and three days to complement the above clinical examination and may include:
***Routinely available:***
• Full blood examination (Hb, WCC and differential count).
• Acute phase reactants (e.g., C-reactive protein, ESR, fibrinogen).
• Routine biochemistry (U&E/Cr, LFTs, LDH).
• Muscle enzymes (e.g., troponin, CKMM and CKMB, aldolase);
• Bloods for cholinesterase – (AChE, BChE)^a^ see below for details
• Others, as clinically indicated.
• Carboxyhaemoglobin** –** HbCO (should be undertaken within 2–4 h post flight post flight for accurate measurements due to short half-life). Record time since exposure and/or time of last cigarette.
• Methaemoglobin (should be undertaken within two to four hours post flight for best assessment due to short half-life).
• Neurobehavioural: basic quick (5 min) testing of processing speed using the Symbol Digit Modalities test (SDMT) (oral and written) and/or digit span forwards and backwards is recommended initially, followed by early referral for more detailed neuropsychological testing if required.
***Non-routinely available***
• Blood for neuropathy target esterase (NTE)^a,b^ – see Table 1 for details;
• Urine for OPs^a,b^ – As soon as possible after a fume event: see Table 2 for details; Blood for VOCs^b^ – As soon as possible after a fume event: see Table 2 for details.
Auto-antibodies against neuronal and glial proteins in blood biomarker testing (at present not available): See emerging issues and supplement (emerging issues & appendix 8).

^a^Level of OP exposure may not be high enough to show enzyme inhibition or TCP urinary metabolites. Lack of inhibition or metabolites does not indicate that OP exposure did not take place

^b^Testing is not routinely available and requires specialist laboratories (discuss with your laboratory)

Blood and urine sampling – additional information

• Bloods should be taken to assess plasma and red cell cholinesterase and, where possible, NTE levels, taking into account the clinical presentation of the person, cost and practicality of testing. While not routinely available, urine samples could be taken to assess for specific organophosphates, and blood samples could be used for selected VOCs

**Table 8:** Late/subsequent medical protocol

A late or subsequent presentation relates to first consultations with medical staff that take place a few days, weeks or even months following the fume event. The medical approach is not dissimilar to that recommended for earlier presentations, in that a detailed clinical history of the events and symptoms experienced at the time of the fume event and those since need to be recorded and a formal physical examination pertinent to the presenting symptomatology undertaken.
The recommendations below should be taken as a guide:
**Medical history of event**
• As for Immediate post-flight (see Table 6).
**Clinical examination and general investigations as applicable**
• As for Immediate post-flight (see Table 6).
• Referral for specialist consultation should be considered as appropriate.
Further discussion regarding special investigations appears in the sections below. It is important to understand that in some cases it will be necessary to undertake the investigation in all people who have been affected by a fume event, while in other situations an investigation may be undertaken based on clinical indication. In general terms, it is always important to consider whether a test undertaken will assist in diagnosis or management. Negative tests are as useful, in many circumstances, as tests that are positive. Cost and availability may need to be considered in some cases.

A record of the fume event, with details of technical and engineering follow-up, together with the symptoms and the medical management of people who have been exposed should be documented. Detailed records help to plan further investigations and objectively correlate symptoms and functional disorders in organ systems. The level of detail recorded will depend on the extent and degree of adverse health effects experienced.

#### In-flight (ground or air) medical protocol

The in-flight report should be made by those assisting people who have been affected by the fume event (crew members and passengers), see Table 6. If a trained health care professional (e.g. doctor, nurse or paramedic) is present, a physical examination is strongly recommended.

#### Immediate post flight medical protocol

Ideally, the employer or airline should facilitate the investigations recommended in this protocol. However, in the interim, or if there is an inability to undertake the investigations, symptomatic aircrew or passengers should be sent to the Emergency Room at the nearest hospital. The airline should inform the hospital and aircrew of this recommended practice, so that the following data are recorded, see Table 7.

Undertaking all the examinations and special investigations suggested may not be possible nor medically indicated in every case. Some investigations require specialist laboratories, and there will be practical issues of availability, timing and cost for procedures and tests. Requests for special investigations should be based on clinical indication in each individual case. Despite these caveats, it is strongly recommended that as much data is collected as is practically possible in every case, as described in Tables 1, 2, 6, 7, 9 and 11. Initial medical assessments may also be required for asymptomatic, or relatively asymptomatic persons, especially aircrew who have been exposed to fumes in an aircraft cabin, see Fig. 2. In fact, aircrew should not be cleared back to fly without an assessment. This may also be important in the conduct of clinical and epidemiological studies in order to address why some aircrew react poorly to fumes in the aircraft cabin while others do not. The decision to what extent medical assessment should be undertaken after a fume event, should take into account a variety of factors including: potential exposure to hazardous substances and the duty of care, symptoms may arise at the time of the event, soon after, or on a prospective basis.

**Table 9:** Immediate post-flight / event: assessment of patients with respiratory-cardiological complaints after a fume event

**Immediate post-flight /event respiratory and heart rate.**
• Auscultation of lung and heart.
• Blood pressure (if measurement and trained personnel available).
• Oxygen saturation SpO_2_ (record inspired oxygen concentration, e.g. air, 2L/min by mask etc.).
• Monitor oxygen saturation if < 95%.
• Spirometry.
• ECG, if indicated e.g., presence of cardiac irregularity.
• Blood tests as clinically indicated.
**Specialist tests within two weeks as required**:
Respiratory function testing within two weeks:
• Detailed lung function tests (spirometry, DLCO and FeNO and / or DLNO, if available).
• Check oxygen saturation SpO_2_.
Consider:
• Arterial blood gas analysis breathing room air at rest – undertake earlier if there is clinical need – see Notes, below.
• Expired nitric oxide (FeNO) if available.
• Exercise testing with oxygen saturation or blood gas analysis.
• Exhaled gas analysis (ergospirometry, if available).
• Blood tests (troponin, if indicated e.g., presence of cardiac irregularity).
• ECG – if clinically indicated.

It is advisable to provide all crew and passengers with an information leaflet list of acute and chronic symptoms associated with Aerotoxic Syndrome—Table 3.

### Respiratory function testing

Routine lung function testing (spirometry) will often yield normal results and symptoms of respiratory tract irritation may be transitory. However, in the early phase spirometry is a simple test measuring basic lung volumes that can be easily performed because it does not require sophisticated equipment. The single breath diffusing capacity for carbon monoxide (DLCO) or nitric oxide (DLNO) are more sensitive tests for gas exchange abnormalities and should be undertaken in all those presenting with respiratory symptoms [209]. Measurement of DLCO and/or DLNO are procedures that detect injuries of lung diffusion but are not available in all medical settings. However, these tests should be arranged in patients with respiratory symptoms, such as cough, shortness of breath, oxygen saturation < 96% and in all those with abnormal spirometric values. The same approach should be applied for exercise testing or ergospirometry. The measurement of expired nitric acid concentration (FeNO), if available is a simple method of assessing pulmonary inflammation. Respiratory orientated exercise testing with measurement of arterial oxygen saturation (oximetry) or arterial blood gas analysis are sensitive for respiratory and cardiac dysfunction and are recommended in cases of acute and especially of persistent respiratory symptoms. Respiratory provocation testing by use of methacholine is sometimes indicated in cases of suspected fume event-induced irritant asthma.

Chest X-rays are routine, and the more sensitive high-resolution computed tomography should be considered. These are seldom performed at the time of injury or soon after. In the presence of persisting respiratory symptoms, radiological examination of the lungs should be arranged.

For recommended testing see Tables 9 and 10.

**Table 10:** Late / subsequent – if respiratory or cardiological symptoms persist over weeks or months

If significant respiratory / cardiac symptoms are present or continue, consider referral to a respiratory specialist / pulmonologist and / or a cardiologist for an opinion and consideration of the following:
• Repeat routine lung function tests (spirometry, diffusing capacity).
• Static lung volumes.
• Percutaneous oxygen saturation or arterial blood gas analysis, as indicated.
• Appropriate radiology, for example, chest X-ray, high resolution lung scan (HRCT chest).
• Respiratory orientated exercise test or screen with six-minute walk test.
• Respiratory muscle strength testing.
• Bronchial provocation (methacholine, mannitol or other agent) testing.
• Blood tests as clinically indicated.
• Specific cardiac function tests as appropriate.
• Exercise testing with oxygen saturation or blood gas analysis.

### Cardiac function testing

For those presenting with cardiac symptoms, e.g. irregular or rapid heart rates or chest pain, a chest x-ray and ECG (including 24-h ECG) should be performed. Prompt referral to a cardiologist is indicated for those with severe or continuing symptoms. Cardiac monitoring may be warranted [210]. Echocardiography and stress testing should be considered and in those with possible positional orthostatic tachycardia (POTs) syndrome tilt table testing is indicated. For recommended testing see Tables 9 and 10.

### Neuro assessment

Gross neurological status (balance, muscle weakness, numbness, reflexes) can possibly identify balance problems, muscle fasciculations and reduced sensation [118]. In most patients with probable Aerotoxic Syndrome, nerve conduction was found to be unremarkable despite a credible and similar pattern of complaints between patients. However, a few studies report mild sensory polyneuropathy [23, 26, 32]. For recommended testing see Table 11.

**Table 11:** Neurological / neurobehavioural assessment

**Immediate post-flight/event**
• Full general medical assessment.
• Detailed neurological assessment and examination.
• Objective assessment of vestibular function.
• MRI brain scan.
• Consider referral to a neurologist for severe neurological symptoms and signs.
**Late/subsequent**
If symptoms persist over weeks or months:
• Full general medical assessment.
• Detailed neurological assessment and examination.
• Objective assessment of vestibular function.
• MRI – Refer to methodology in [132].
• PET / SPECT – Refer to methodology in [117].
• EMG / ENG—polyneuropathy;
• Skin biopsy / IENF (intraepidermal nerve fibres) – Small fibre neuropathy (Lauria et al. 2010)^a^.
**Neurocognitive/ Neurobehavioural**
Neurocognitive tests that are deemed applicable include the following areas:
• Processing speed, written and oral.
• Attention and concentration.
• Reaction time to stimuli.
• Sequential reaction time.
• Complex problem solving.
• Short and long term visual and verbal memory.
• Cognitive flexibility / capacity to change direction.
Neurocognitive testing:
• Coding test from WAIS.
• Symbol Digit Modalities Test (written and oral versions), see Section 1B.
• CALCAP – Simple and choice reaction time tests.

Note: All tests should be able to be administered by medical personnel

^a^Lauria G, et al. European Journal of Neurology. 2010; 17: 903–12. https://doi.org/10.1111/j.1468-1331.2010.03023.x

Neurocognitive tests that are deemed applicable include the following areas: processing speed, attention and concentration, reaction time to stimuli, sequential reaction time, complex problem solving, short and long term visual and verbal memory and cognitive flexibility/capacity to change direction. For recommended testing see Table 11. If neurobehavioural/ neuropsychological symptoms persist over weeks or months, we recommend a neuropsychological test battery as in Table 12.

**Table 12:** Recommended neuropsychological test battery if neurobehavioural / neuropsychological symptoms persist over weeks or months

**Formal neurocognitive testing:**
• Tests for processing speed such as the Coding Test or Wechsler Adult Intelligence Scale (WAIS), Symbol-Digit Modalities Test (written and oral), Symbol Search (WAIS) and Trail Making Test A.
• Tests of new learning, such as the Austin Maze and the Rey Auditory Verbal Learning Test (RAVLT).
• Memory tests, such as those in the Wechsler Memory Scale, including visual and verbal memory.
• Problem solving tests, such as the Category Test. The Wisconsin Card Sorting Test and the Stroop Test.
• Fine motor tests, such as the Reitan Finger Tapping Test of manual speed, the Grooved Pegboard Test of manual dexterity and the Dynamometer Grip Strength Test.
• In case of sleep disturbances consider full polysomnography;
• Boston Naming Test of language skills.

Similar and alternative tests have been utilised in other studies with aircrew after a fume event [18, 22, 109, 111]. Tests available may vary in different countries; however, most of the tests listed are universal.

### Treatment

Treatment for Aerotoxic Syndrome is symptomatic. Removal from environment may be required for short duration or longer term.

## Discussion

During routine flight, continual low-dose (i.e., chronic) exposure to oil fumes, with less frequent acute exposures, is occurring. Aircraft cabin air can become contaminated by hydraulic fluids along with many different volatile lubrication oil compounds and their thermal degradation products.

Prudence requires that we consider more than one contaminant as being involved in the etiology of ill health. There has been a focus on one particular isomer of the OP in the oils, and this has led to the neglect of other contaminants, the combined mixture, and chronic low-level exposures. The latter would be especially relevant in what is described as Aerotoxic Syndrome.

To date, there has been inadequate recognition of both the types and implications of exposures to contaminated cockpit and cabin ventilation air supply that occurs as a feature not only of the design of the systems utilised, but less frequently during abnormal or failure conditions related to these systems. Our proposed medical protocol, combined with a narrative review, provides a better understanding of exposures sourced to the aircraft ventilation air supply, adverse effects reported, and how to respond to people after aircraft contaminated air and fume events. This should also aid in the systematic gleaning of epidemiological data of aviation workers and the public who travel in aircraft.

Although a variety of sources can contaminate the cockpit and cabin air, by-products of oil and hydraulic fluids have been the key focus of concern. The organophosphate TCP, used in engine oils, has been the major focus of concern. Most attention has been given to the tri-ortho isomer T*o*CP in the oils at very low levels due to its neurotoxic properties associated with OPIDN, a high dose effect of exposure. The neurotoxic properties of the other ortho isomers, at over 600,000 times higher levels in the TCP used in the oils and over 6 million times greater in toxicity than T*o*CP, have been ignored [62]. Neurotoxicity of the non-ortho isomers of TCP in the oils at around 3%, have also been ignored. As an example, the meta and para isomers of TCP showed demyelination of neurons yet did not cause paralysis [65]. The various OPs in the hydraulic fluids can range from low levels up to 80%. The effects of the complex mixture consisting of a wide range of VOCs associated with thermally degraded engine oils have not been given proper consideration. The other compounds including amines, UFPs, carboxylic acids in the base stock of the oils, and carbon monoxide, all need to be given due attention. While cholinesterase effects of OPs have been shown to take up to 4 h to occur in mice, the effects of CO would be quite rapid.

Various engine bleed air and cabin air monitoring studies have been undertaken during routine flight operations. TCP used in engine oils and the hydraulic fluid OPs have frequently been identified at low levels. A wide range of VOCs, many of which have been reported in oil pyrolysis studies, have been identified both in the cabin and in bleed air monitoring investigations. When detected, the levels identified have almost always been well below occupational exposure limits. Despite such exposure limits being applicable to ground based industrial environments (i.e. workplaces), they have often incorrectly been cited to suggest that the air in aircraft is better than in offices and other ground-based environments, including workplaces. However, the meta and para isomers of TCP, as an example, do not have an exposure limit value. The use of exposure limits and individual substances fails to consider the complex mixture and other toxicity factors associated with the various compounds. Passengers travel in the same environment as the aircrew, yet their exposure is not considered. The findings support that chronic, low-level exposure is occurring in normal operations at background levels as well as during transient fume events.

The frequency of fume events is a continuing debate. The focus has been on the less frequent abnormal or failure conditions, while ignoring that low-level exposure to background levels of oil fumes occurs in normal operation as well as in acute transient exposures and fume events. Therefore, exposures are happening in two distinct ways: 1) continual low-level background exposure and transient exposures associated with phases of flight when oil seals are known to be less effective, and 2) the far less common abnormal or failure events (i.e., acute exposure events). However, lack of training and awareness, lack of comprehensive reporting regulations, under-reporting, and the lack of contaminated air detection systems further hinder the full understanding of how often these exposures and fume events occur. Frequency should now be seen in relation to system design factors that enable exposure to occur, rather than in terms of the number of reports submitted [5, 6].

There have been limited efforts related to biomonitoring for OPs and VOCs. Regarding the OPs, the focus again has been on T*o*CP, despite the increased levels and higher toxicity of the other mono- and di-ortho isomers of TCP and the complex mixture used in the commercial formulation of TCP in the oils. Urine metabolites of TBP and TPP used in hydraulic fluids were significantly raised over controls, while metabolites of TCP non-ortho isomers were hardly detected. The methodological limitations of identifying these TCP isomers in urine has been acknowledged [211]. Cholinesterase enzyme activity has limitations. Due to the interindividual variability of cholinesterase values, a baseline reference level must be undertaken. Measurable activity levels of AChE and BChE or mass spec measurements of aryl-phosphate bound to the active site serines of Ache or BChE appear to occur only after higher level OP intoxications, which are not generally expected after fume events. BChE may be a more suitable activity enzyme. Development of more accurate mass spectrometry methods, requiring one sample only, is near completion. While VOCs can be investigated in blood after fume events, these tests are costly, very specific, and not readily available.

Aerotoxic Syndrome encompasses a wide constellation of symptoms and health disorders. These include neurological, neurobehavioural, respiratory, cardio, irritant, sensitising, gastrointestinal, rheumatological, fatigue, chemical sensitivity and others. Symptoms can be prompted by a single high dose or repeated, or prolonged low-level exposures. The adverse physiological effects have consistently, but in a non-uniform manner, been reported globally over many decades in association with air supply contamination.

Symptom intensity varies between individuals for a variety of reasons including repeated exposures, intensity and duration of exposures, genetic variability, and related individual susceptibility factors. While neurotoxic effects are of primary concern, there is a wide range of other organ systems that are involved, continuing to show a consistent but diverse pattern. A number of the adverse effects reported are consistent with EU health-based hazard classifications associated with the individual compounds in the oils and fluids above the hazard classification levels [149, 150, 152].

One of the more comprehensive descriptive studies reported the broad range of short and long-term adverse effects in 274 pilots who had flown on the BAe 146 [25, 26]. This aircraft was acknowledged to have higher oil leakage rates than most. Thirteen percent of the 274 pilots reported a range of chronic ill health outcomes and a permanent loss of fitness to fly. This was 37%—433% above a literature search of civilian and military pilot loss of medical certification respectively, for all reasons [25, 26].

It is regularly suggested that the symptom pattern is too diverse and non-specific to be a syndrome, effects are not seen in all those exposed, and symptoms are not consistent with the OP effect of OPIDN. OPIDN is a known high-dose effect from certain OP exposures, yet this is not being seen in aircrew, with chronic low-dose effects being ignored. Additionally, this fails to consider that air monitoring studies and the engine and air supply systems enable low-level contamination in normal flight. The symptoms associated with chronic low-dose exposure to OPs are acknowledged to be non-specific and diffuse, and not associated with cholinergic effects. Inhibition of enzymes at OP levels that do not affect AChE or BChE activity levels, has been shown in vivo by TAP metabolites, including those reported to be linked to cognition [66, 212, 213].

There are emerging areas to be considered, including repeat exposure to low levels of a complex mixture, exposure to UFPs, dose, and effects other than neurotoxicity. Oil exposed to high temperatures generates high levels of UFPs (up to > 500,000 – 2.8 × 10^6^ particles / cm^3^) to which various substances, including OPs, can adhere. The nanoparticles can then act as a trojan horse when inhaled, crossing the blood–brain barrier with direct access to the brain / CNS [2]. The pattern of exposure is consistent with acute, overlayed on chronic effects of exposure to OPs, with constant low-level dose exposure to nanosized oil droplets significantly raising the internalised dose, with considerable cumulative effects expected. While total dose over time is seen as a key factor, this is generally not considered.

As an example, while TCP measurements are seen as low in the nano or low microgram range, Table 4 suggests that total TCP over a crew’s working lifetime could be far higher at up to > 300 mg based on studies commonly cited, in which fume events were not regarded as occurring. While there is continuing debate about whether any long-term effects are possible and associated with contaminated bleed air, the collection of data in a consistent and standard manner will further support the existing data documenting that such effects are occurring, including signs, symptoms, and diagnoses, many of which were confirmed with oil leakage events [19, 22, 23, 25-27, 29, 31, 120, 151, 155, 214]. There is a range of serious neurodegenerative diseases associated with OPs, and emerging data support disease occurrence in other organs.

It is often cited that there is no causal link between adverse effects reported by aircrew and exposure to the aircraft bleed air contaminants. This relates to the incorrect T*o*CP/OPIDN argument cited above, the absence of sensor equipment in aircraft to measure exposure levels, and the failure to consider the acute, overlayed on chronic patterns of exposure, the complex nature of the mixtures, and cumulative effects. Also, in vitro / in vivo studies have not been adequately undertaken to reflect the appropriate exposure scenario, considering both inhalation, and chronic low-level effects. In addition, epidemiological data are often ignored.

There has been criticism within the aviation industry that the epidemiological data collected are not robust or specific enough, and that the data lack statistical power. Criticism of evidence is expected from those with an interest in denying connections between exposure and effect [215]. While there is a range of epidemiological study types, none should be ignored [216] in assessing the body of evidence for demonstrating the health effects of exposures. The body of evidence for inferring causation is populated by contributions derived and made available from different epidemiological approaches. These contributions range from the less informative case studies to descriptive analyses comparing observed to expected numbers of illness events across exposed and unexposed groups of people. The contribution to causation of these types of studies is limited, being primarily related to hypothesis generation. Observational studies, including case–control (for rare outcome events) and cohort studies (for more common outcomes), are more influential to discussions about causation. The most influential of the epidemiological study designs to discussions about causation are double-blind randomised controlled trials (RCTs).

Given the above hierarchy of epidemiological studies, to further address question of causation, appropriately designed observational studies would be utilised instead of RCTs because it would not be ethical to undertake RCTs, exposing people to toxic fumes. The most informative of the available observational epidemiological study designs is that of the prospective cohort study. In this design, a large enough group of exposed people is followed up for a long enough time for health outcomes to manifest, and their experience is compared to a large enough unexposed group followed up for an equally long period of time. While this would be ideal, it is inappropriate for addressing Aerotoxic Syndrome for the reason that rare health events never achieve statistical power to demonstrate a difference unless many thousands of people were to be adequately followed up. Feasibility and cost considerations would rule such a design impractical. Therefore, when health outcomes are rare, the optimal design from a cost perspective would be that of the case–control study.

In terms of the evidence to date of health effects associated with contaminated aircraft and fume events, numerous case reports, case-series, and descriptive studies have been undertaken and they should not be ignored.

Importantly, there has been an over-reliance on the lack of exposure data collected during fume events, despite there being no sensors to take measurements. This has been at the expense of failing to consider that the air supply and engine system designs enable exposure to oils to occur in both normal operations (ground and air) and abnormal conditions. There are acknowledged difficulties in identifying the source of oil fumes, due to events often being transient, lack of real time sensors, and maintenance investigation procedures more suited to bigger smoke / failure events. However, positive oil/hydraulic findings have been documented in association with adverse effects and fume events. Of 15 fume events investigated, 87% involved positive maintenance findings of oil fumes, with another said to be likely sourced to oil leakage [26].

The risk assessments thus far have considered effectively T*o*CP (and not the more plentiful and more toxic mono- and di-ortho isomers) exposure and limited individual substances, which are reported well below exposure limits, yet ignoring the areas identified above. Industrial occupational exposure limits, where they exist, are not protective for aircrew or passengers in aircraft, exposed continually to this complex, thermally degraded mixture.

We consider that prolonged, chronic exposure of aircrew to an aerosol of oil-bearing nanoparticles is a significant feature in the aetiology of the pattern of illness being manifested. Adverse effects continue to occur. Prior attempts to gather epidemiological data have been neither timely, comprehensive, nor systematic. Therefore, this comprehensive medical protocol, set out with a narrative review, is an essential step forward to enable a greater understanding of this specialised environment, with further epidemiological data to be systematically gathered to more precisely explicate risks. The features associated with exposure to oil, other supply air fumes, and adverse effects outlined here is suggestive of a causal link between exposure and health effects in aircrew and passengers. This clearly points to a discreet occupational syndrome that warrants the appropriate gathering of epidemiological data, and the introduction of risk mitigation.

## Conclusion

There is extensive literature supporting exposure to oil, hydraulic and other supply air fumes entering the aircraft breathing air during routine as well as abnormal flight conditions. There is a consistent diffuse pattern of adverse effects documented in aircrew and some passengers. This narrative review and protocol of the documentation of people exposed to fume events related to the aircraft supply air provides the first comprehensive and systematic approach in documenting and gathering further epidemiological data on what is a discreet and emerging occupational health syndrome.

### Supplementary Information

**Additional file 1.** 

## Data Availability

DOIs and hyperlinks have been provided throughout the literature cited where applicable. Additional information is provided in the attached supplement, which entails the full medical protocol / narrative review for those seeking further information.

## Abbreviations

AChE: Acetyl cholinesterase
APU: Auxiliary power unit
BChE: Butyrylcholinesterase
BNA: Beta-naphthylamine
CNS: Central nervous system
CO: Carbon monoxide
D*o*CP: Di-ortho-cresyl phosphate
M*o*CP: Mono-ortho-cresyl phosphate
NTE: Neuropathy target esterase
OP: Organophosphate
OPICN: Organophosphorus- induced chronic neurotoxicity
OPIDN: Organophosphorus ester-induced delayed neurotoxicity
PAN: N-phenyl-1 napthylamine
PBN: N-2-naphthylaniline
PNS: Peripheral nervous system
SVOC: Semi-volatile organic compounds
TBP: Tributyl phosphate
TCP: Tricresyl phosphate
T*o*CP: Tri-ortho-cresyl phosphate
T*p*CP: Tri-para-cresyl phosphate
TPP: Triphenyl phosphate
TXP: Trixylyl phosphate
UFP: Ultrafine particles
VOC: Volatile organic compounds

## Footnotes

1 ^.^ McDonald, M—Medicinal Chemistry, University of Washington, 2015.
