# Correction: Health consequences of exposure to aircraft contaminated air and fume events: a narrative review and medical protocol for the investigation of exposed aircrew and passengers

**DOI:** 10.1186/s12940-023-01025-3

**Published:** 2023-10-28

**Authors:** Jonathan Burdon, Lygia Therese Budnik, Xaver Baur, Gerard Hageman, C. Vyvyan Howard, Jordi Roig, Leonie Coxon, Clement E. Furlong, David Gee, Tristan Loraine, Alvin V. Terry, John Midavaine, Hannes Petersen, Denis Bron, Colin L. Soskolne, Susan Michaelis

**Affiliations:** 1 Respiratory Physician, St Vincent’s Private Hospital, East Melbourne, Australia; 2 Institute for Occupational and Maritime Medicine, University Medical Center Hamburg-Eppendorf, Hamburg, Germany; 3 European Society for Environmental and Occupational Medicine, Berlin, Germany; 4 University of Hamburg, Hamburg, Germany; 5 Department of Neurology, Medisch Spectrum Twente, Hospital Enschede, Enschede, The Netherlands; 6 Centre for Molecular Biosciences, University of Ulster, Coleraine, Northern Ireland UK; 7 Department of Pulmonary Medicine, Cl?nica Creu Blanca, Barcelona, Spain; 8 Clinical and Forensic Psychologist, Mount Pleasant Psychology, Perth, Australia; 9 Departments of Medicine (Div. Medical Genetics) and Genome Sciences, University of Washington, Seattle, USA; 10 Centre for Pollution Research and Policy, Visiting Fellow, Brunel University, London, UK; 11 Technical Consultant, Spokesperson for the Global Cabin Air Quality Executive, London, UK; 12 Department of Pharmacology and Toxicology, Medical College of Georgia, Augusta University, Augusta, USA; 13 Lifemedic Bilthoven, Bilthoven, The Netherlands; 14 Faculty of Medicine, University of Iceland, Akureyri Hospital, Akureyri, Iceland; 15 Federal Department of Defence, Civil Protection and Sport (DDPS), Aeromedical Institute (FAI)/AeMC, Air Force, D?bendorf, Switzerland; 16 School of Public Health, University of Alberta, Edmonton, AB Canada; 17 Occupational and Environmental Health Research Group, Honorary Senior Research Fellow, University of Stirling, Scotland / Michaelis Aviation Consulting, West Sussex, England

**Environmental Health (2023) 22:43**

10.1186/s12940-023-00987-8

Following the publication of the original article, the author reported that the link in reference 216 leads to the main WHO website. The link should be updated to https://iris.who.int/handle/10665/347877.

The original article has been updated.
