# Supplementary material for: Health consequences of exposure to aircraft contaminated air and fume events: a narrative review and medical protocol for the investigation of exposed aircrew and passengers
Source: Environ Health. 2023 May 16;22:43. doi: 10.1186/s12940-023-00987-8 (PMC10186727; doi:10.1186/s12940-023-00987-8)
Supplement: Supplementary file 1 — Additional file 1. [file 12940_2023_987_MOESM1_ESM.pdf]

Health consequences of exposure to aircraft contaminated air and fume events: A narrative review and medical protocol for the investigation of exposed aircrew and passengers

Practical guideline prepared by the International Fume Events Task Force (chaired by S. Michaelis) with working groups from the DiMoPEx COST-Action* and Collegium Ramazzini**

Jonathan Burdon,[1] Lygia Therese Budnik,[2 ***] Xaver Baur,[3] C. Vyvyan Howard,[4] Gerard Hageman,[5] Jordi Roig,[6] Leonie Coxon,[7], Clement E. Furlong,[8] David Gee,[9] Tristan Loraine,[10] Alvin V. Terry Jr.,[11] John Midavaine,[12] Hannes Petersen,[13] Denis Bron,[14] Colin L. Soskolne,[15] Susan Michaelis.[16]

1. Consultant Respiratory Physician, St Vincent's Private Hospital, East Melbourne, Australia.
2. DiMoPEx-Chair, University Medical Center Hamburg-Eppendorf, Institute for Occupational and Maritime Medicine, Hamburg, Germany.
3. European Society for Environmental and Occupational Medicine, President; Emeritus University of Hamburg, Germany.
4. Emeritus Professor, Centre for Molecular Biosciences, University of Ulster, United Kingdom.
5. Department of Neurology, Medical Spectrum Twente, Hospital Enschede, The Netherlands.
6. Department of Pulmonary Medicine. Clínica Creu Blanca, Barcelona, Spain.
7. Clinical and Forensic Psychologist, Mount Pleasant Psychology, Perth, Australia.
8. Departments of Medicine -Div. Medical Genetics and Genome Sciences, University of Washington, Seattle, United States.
9. Visiting Fellow, Centre for Pollution Research and Policy, Brunel University, London.
10. Airline Captain (ret), Technical consultant, Spokesperson for the Global Cabin Air Quality Executive, London, United Kingdom.
11. Regents Professor and Chair, Department of Pharmacology & Toxicology, Medical College of Georgia, Augusta University, Augusta, USA.
12. Sports Doctor (Specialist Orthomolecular Medicine), Lifemedic Bilthoven, The Netherlands.
13. Faculty of Medicine, University of Iceland, Akureyri Hospital, Iceland.
14. Chief Flight Surgeon, Head of Aviation Medicine (AeMC); Federal Department of Defence, Civil Protection and Sport (DDPS); Air Force; Aeromedical Institute (FAI) / AeMC, Switzerland.
15. Professor Emeritus, University of Alberta, Edmonton, Canada.

16. Honorary Senior Research Fellow, Occupational and Environmental Health Research Group, University of Stirling / Michaelis Aviation Consulting, West Sussex, England.

Corresponding author: Susan Michaelis: susan@susanmichaelis.com

Footnotes:

*Diagnosis, Monitoring and Prevention of Exposure related Noncommunicable diseases, DiMoPEx (www.cost.eu/actions/CA15129) is EU-COST founded action CA 15129, with partners from 27 countries (Late action chair: Lygia Therese Budnik,)

**The Collegium Ramazzini is an independent, international academy founded in 1982 comprising 180 internationally renowned experts in the fields of occupational and environmental health. The mission of the Collegium Ramazzini is to advance the study of occupational and environmental health issues and to be a bridge between the world of scientific discovery and the social and political centers that must act on the discoveries of science to protect public health (www.collegiumramazzini.org).

***The authors recognise Prof. Dr Lygia Therese Budnik for the important part that she played in developing this manuscript. Dr Budnik died on 20 November 2020 [we dedicate this article to her memory].

ABSTRACT

Pyrolysed or thermally degraded engine oil and hydraulic fluid fumes contaminating aircraft cabin air conditioning systems have been recognised and well documented since the 1950s. Research suggests that inhalation of these potentially toxic fumes causes ill health. The evidence that fumes can enter the aircraft cabin is not widely accepted, particularly by the airline industry and its regulators. However, there is a documented history spanning decades that associates the onset of ill health with fume exposure in most cases. Evidence suggests that cumulative exposure to regular small doses of toxic fumes is potentially damaging to health and may be exacerbated by a single higher-level exposure. Whilst organophosphates have been the main subject of interest, oil fumes in the air supply also contain ultrafine particles and numerous volatile organic hydrocarbons, which are likely to be damaging to health. Assessment is made more complex because of the limitations of considering the toxicity of individual substances in complex heated mixtures.

The lack of recognition by some, of illness caused by exposure to pyrolysed engine oil, de-icing and hydraulic fluid in aircraft ventilation supply air is likely to be due to lack of knowledge and clinical acumen. The easier finding of more clinically understood diagnoses in unexplained clinical presentations is recognised.

When examined using the Bradford Hill features of evidence and based on a more probable than not approach, the association between fume exposure and ill health leads to the inference that illness caused by fume exposures is a real clinical entity. There is a need for a systematic and consistent approach to diagnosis and treatment of people who have been exposed to toxic fumes in aircraft cabins and education of all professions involved.

This medical protocol has been written by internationally recognised experts and presents a consensus approach to the recognition, investigation and management of people suffering from the toxic effects of inhaling pyrolysed engine oil and other fluids contaminating the air conditioning systems in most aircraft. A best practice medical protocol is outlined including actions and investigations for in flight, immediately post flight and late subsequent follow up.

INDEX

ABSTRACT 3

INDEX 3

ACRONYMS 8

INTRODUCTION 10

AIM OF THIS PUBLICATION 12

NOMENCLATURE 13

TECHNICAL BACKGROUND OF CABIN AIR CONTAMINATION INCIDENTS (FUME EVENTS) 14
Aircraft air supply: 14
Oil and hydraulic fluid fumes: 14
Fume events: 16
Hazard classifications: 16
Oil and hydraulic fluid compounds: 16
Complex mixture: 17
Air quality studies: 17
Occupational exposure limits: 18

AVIATION INDUSTRY SCIENTIFIC INITIATIVES RELATED TO FUME EVENTS 20
Reviews of oil fume exposures: 20
Air monitoring studies: 20
Health risk assessments: 20
TCP/oil pyrolysis studies: 20
Government and regulator actions and studies: 21
Care pathways: 21

CLINICAL EFFECTS DESCRIBED BY PEOPLE AFTER AN AIRCRAFT FUME EVENT 23
General: 23
Aerotoxic Syndrome: 23
Misdiagnosis: 24
Collection of data: 24

PROPOSAL FOR THE DOCUMENTATION OF A FUME EVENT, CLINICAL HISTORY AND PHYSICAL EXAMINATION 26
General: 26
Collation of data: 26
Assessments: 28

SECTION 1: POST-EVENT MEDICAL PROTOCOL 30
Section 1A. In flight (ground or air): 31
Summary *31*
Details *31*
Environmental observations 31
History of symptoms and measures related to the fume event 32
Physical examination 32
Section 1B. Immediate post-flight/event: 33
Summary *33*
Details *33*
a. Medical history of event 33
b. Clinical examination (symptom related) 33
c. General investigations 34

(ii) Non-routinely available 35
(iii) Other tests as clinically required 35
(iv) Blood and urine sampling – additional information 35
Blood analysis for acetylcholinesterase, butyrylcholinesterase and neuropathy target esterase 36
Blood analysis: volatile organic compounds (see Section 2 and Appendix 6) 39
Urine analysis: organophosphates (see Section 2 and Appendix 6) 39
Section 1C. Late/subsequent 41

SECTION 2: FURTHER INFORMATION ON HUMAN BIOMONITORING 42
Section 2A. Immediate post-flight/event 42
Effect-monitoring: acetylcholinesterase, butyrylcholinesterase, neuropathy target esterase - General 42
Effect-monitoring: Triaryl phosphate organophosphate biomarkers 43
Mass spectroscopy analysis of triaryl phosphate biomarkers 43
Enzyme activity levels of cholinesterases and other esterases. 44
Human biomonitoring: volatile organic compounds and organophosphates 46
Blood analysis for volatile organic compounds 47
Urine analysis for organophosphates 47
General 49

SECTION 3: LUNG/HEART 50
General background: 50
Respiratory system: 50
Respiratory function testing 52
Heart: 53
Recommended testing 55
Section 3A: Immediate post-flight /event 55
Specialist tests within two weeks as required: 55
Section 3B: Late / subsequent – if symptoms persist over weeks or months 56

SECTION 4: NEUROLOGICAL 58
General 58
Target organ toxicity of the nervous system: 58
Non-cholinergic mechanisms: 58
Various non-cholinergic mechanisms have been described: 59
Overview of organophosphate toxicity: 62
Organophosphate-induced toxicity due to acetylcholinesterase inhibition: Cholinergic toxicity 62
Organophosphorus-induced delayed neurotoxicity 63
Organophosphorus-induced chronic neurotoxicity 64
Low-dose exposure 64
Summary 64
Central nervous system: 66
Neurobehavioural and neuropsychological effects 68
Peripheral nervous system: 69
Summary: 70
Recommended testing: 71
Section 4A: Immediate post-flight/event 71
Section 4B: Late/subsequent 71
Section 4C: Neurocognitive 72

SECTION 5: IRRITANTS 74
Management: Immediate post-flight / Late/subsequent: 75

SECTION 6: SENSITISATION 76
Management: Immediate post-flight / Late/subsequent: 76

SECTION 7: SKIN 77
Management: Immediate post-flight / Late/subsequent: 77

SECTION 8: GASTROINTESTINAL – IMMEDIATE POST-FLIGHT / LATE/SUBSEQUENT 78
Management: Immediate post-flight / Late/subsequent 78

SECTION 9. OTHER 79
Fatigue/chronic fatigue: 79
Chemical sensitivity: 79
Reproductive symptoms: 79
Malignancy / mutagenicity: 80
Susceptibility to infection: 82
Sleep disturbance: 82
Visual acuity and eye disorders: 82
Joint aches and pains: 82
General information: 83

SECTION 10: DIAGNOSTIC CODING 85

SECTION 11: EMERGING ISSUES 86
A. Ultrafine particles: 86
B. Autoantibodies against neuronal and glial proteins in blood biomarker testing: 87
C. Increased genetic susceptibility to toxic compounds: 88
C. Low-level repeat exposure to mixtures: 90
D. Chronic low-level exposure to OPs: 91
E. Acute versus chronic exposure: 92
F. Dose: 93
G. Endocrine disruptors: 95
H. Causal connections: 95

SECTION 12: CONCLUSIONS 99

APPENDICES 100
Appendix 1A: International Civil Aviation Organization (ICAO): Examples of potential types of aircraft fumes. 100
Appendix 1B: American Society of Heating, Refrigerating and Air-Conditioning Engineers (ASHRAE) Guideline 28-2012: Air quality within commercial aircraft. 101
Appendix 1C: Frequency of occurrence of contaminants in bleed air samples. 102
Appendix 2: Aerotoxic Syndrome – List of symptoms: Acute & Long-term. 104
Appendix 3: Aerotoxic Syndrome symptoms. 107
Appendix 4: AIRCRAFT CABIN FUME EVENTS – PROCEDURES TO FOLLOW 108
Appendix 5: Symptoms during / after a fume event 124
Appendix 6: Blood and urine testing for VOCs and OPs 126
Appendix 7: Supplementary table: Human biomonitoring 126
Appendix 8: Procedures for Determination of Autoantibodies to Neural Proteins. 129
Appendix 9A: Hazard classifications associated with substances in oils based on EU CLP inventory database. 132
Appendix 9B: Hazard classifications associated with substances in the hydraulic (HF) and Deicing (DI) fluids based on EU CLP based inventory database. 133

REFERENCES 136

TABLES

Table 1: Blood sampling for acetylcholinesterase (AChE), butyrylcholinesterase (BChE) and neuropathy target esterase (NTE) 37

Table 2: Internalised dose during crew working lifetime 94

Table 3: The Bradford Hill approach applied to Aerotoxic Syndrome (2017) 96

FIGURES

Figure 1: Recommended investigation and management approach for persons exposed to fume events 30

Figure 2: Schematic drawing of a nerve cell 60

Figure 3: Schematic drawing of signal transfer between nerve cells 61

ACRONYMS

AChE	Acetylcholinesterase
APU	Auxiliary power unit
ASHRAE	American Society of Heating, Refrigerating and Air-Conditioning Engineers
BChE	Butyrylcholinesterase
BNA	Beta naphthylamine
CBDP	Cresyl saligenin phosphate
CNS	Central nervous system
CO	Carbon monoxide
DLCO	Diffusing capacity for carbon monoxide
DLNO	Diffusing capacity for nitric oxide
DOCP	Di-ortho-cresyl phosphate
ED	Endocrine disruptor
FeNO	Fractional concentration exhaled nitric oxide
ICAO	International Civil Aviation Organization
MOCP	Mono-ortho-cresyl phosphate
MSDS	Material safety data sheet
NTE	Neuropathy target esterase
OP	Organophosphate
OPICN	OP-induced chronic neurotoxicity
OPIDN	OP-induced delayed neurotoxicity
PAN	Phenyl-α-naphthylamine
PNS	Peripheral nervous system
TAP	Triaryl phosphate
TBP	Tributyl phosphate
TCP	Tricresyl phosphate
TIPP/PIP (3:1)	Isopropylated phenyl phosphates /
T*m*CP	Tri-meta-cresyl phosphate
T*o*CP	Tri-ortho-cresyl phosphate
T*p*CP	Tri-para-cresyl phosphate
TPP	Triphenyl phosphate

TXP	Trixylyl phosphate
UFP	Ultrafine particles
VOC	Volatile organic compound

INTRODUCTION

Contaminated aircraft cabin air incidents, commonly identified as 'fume events', were first described in military aircraft in the 1950s (1-5). The onset of fume events coincided with the introduction of synthetic jet engine oils, used in high performance turbine engines (6), and the practice of supplying the aircraft breathing air directly from the engines without undergoing filtration. Over the following decades, continuing reports describing symptomatic aircrew correlated with fume events (7-14). Subsequently, a number of studies have identified adverse effects reported by aircrew as well as medical findings and diagnoses associated with fume events (15-31).

For several decades, medical diagnostics have been used to check possible functional or organ related impacts that correlate with the reported symptoms. These include both acute and chronic health effects: neuropsychological/neurological (32-39); respiratory (40-43); biomarkers (44, 45); and biomonitoring (46-48). Over the years, there has been a lack of timely and systematic investigation of fume events. There has also been a failure to measure and identify potential contaminants at the time of exposure. The lack of exposure data and documentation have hindered not only the assessment of measured abnormalities and their relationship to clinical symptoms, but also the development and implementation of preventative and therapeutic strategies.

Symptoms, signs, objective measurements and diagnoses are recorded in many cases by initial responders, medical personnel and by those people who have been affected. However, there has been no consistency in the way diagnoses are reached and in record keeping. Thus, there is an urgent need for the development of a consistent, internationally accepted medical protocol to facilitate the recognition of health effects associated with fume exposure in aircraft cabins and cockpits. A medical protocol is needed to establish aircraft-related passenger and, in the context of aircrew, occupationally induced disease (28, 49, 50). The goal is to establish a standardised global basis for better documenting and addressing the health impacts of inhaling these fumes on aircraft.

There has been some effort by the aviation industry and governments to outline medical guidance after fume events (51, 52). Generally, the guidance is not specific and there is inconsistency from

region to region and sector to sector in how fume events are recorded. The proposed protocol in this document is a step forward in assembling a uniform database of fume events, their effects (short and long term), diagnostic and treatment options and related outcomes. The proposed protocol is an evidence-based work-in-progress that will be updated as the field progresses.

This document is based on the medical and scientific expertise of a group of international experts and medical centres, including peer-reviewed published findings and grey literature.

AIM OF THIS PUBLICATION

In this protocol, we summarize the data systematically to substantiate the social recognition of illness after cabin air contamination. Its purpose is to document a recommended approach to the observation, measurement and recording of symptoms, signs and treatment (if any), and subsequent management of afflicted persons and their health outcomes. Presentation of health effects are considered in the following timeframes:

- In-flight.
- Immediate post flight (within one to two days).
- Late/subsequent (beyond two days).

The medical management and specific investigations will vary in each case, depending on specific technical and individual contextual factors. Medical management is related to the presenting symptomology at the time of examination.

Although important in the occupational health and safety context, it is not the intention of this document to address the issue of routine monitoring of aircraft cabin air for the presence of toxic fumes.

NOMENCLATURE

Nomenclature for illness resulting from a fume event needs to be agreed upon before proceeding further. In this regard, the term 'Aerotoxic Syndrome' (AS), was suggested by Winder et al. in 2000 (11) to describe the constellation of symptoms experienced by people exposed to fume events.

The term Aerotoxic Syndrome has been used for many years, but it has not received broad acceptance for a number of reasons. The term has been widely criticised by the airline industry and others because the term 'aerotoxic' could be emotive. Additionally, the term 'syndrome' has been questioned because not all the possible clinical manifestations are present in every case. In the medical context, the term 'syndrome' is defined as a clinical picture characterised by a certain constellation of signs and symptoms. Not all signs and symptoms are present in every case due to differences in exposure, genetic susceptibility and other factors. 'Aircraft related illness' has been suggested elsewhere as a suitable term (53).

Given the above, we submit that the term 'Aerotoxic Syndrome' is medically and technically reasonable. We justify our reasons below and note that it is the original term coined by Winder et al. in 2000 (11).

TECHNICAL BACKGROUND OF CABIN AIR CONTAMINATION INCIDENTS (FUME EVENTS)

Understanding the technical background of a cabin air contamination incident is necessary to develop a human investigative and biomonitoring strategy and to implement appropriate investigative techniques.

Aircraft air supply:

In flight, all modern commercial jet transport aircraft, except for the Boeing 787 Dreamliner, use air compressed within the engine as the source of the air used for aircraft ventilation and pressurisation. This air is technically known as 'bleed air' because it is bled off the compression section of the engine. On the ground, a small jet engine normally located in the tail of an aircraft, known as an Auxiliary Power Unit (APU) frequently provides the ventilation air. The B787 uses electrically compressed air drawn directly in from outside rather than being sourced from the engine/APU.

As in other indoor air environments, the air supply system is designed to dilute airborne contaminants which are sourced to occupants, systems and surfaces (generated internally or externally) that enter the cabin air when ventilating the cabin with outside air. Outside air is used to dilute contaminants in the air and flush them to the outside of the cabin (54). As such, ventilation exchange rates in aircraft are higher than in other indoor settings.

Fumes within the aircraft cabin can be sourced to the aircraft ventilation air supply or to items within the cabin and/or flight deck (55). These can be seen in Appendices 1A and 1B (55, 56). Such sources within the aircraft cabin may include carry-on baggage, disinfectants, disinsectants or galley equipment. Fume sources from within the ventilation air supply can include engine oil, hydraulic or de-icing fluids, fuel and electrical faults. In this document, we focus on fumes sourced to the aircraft ventilation supply.

Oil and hydraulic fluid fumes:

It is now increasingly recognised that pyrolysed engine oil, used in jet aircraft engines, leaks at varying levels into the air supply systems in most jet or turbine powered aircraft (52-54). Oil leakage over the engine seals occurs in three main ways:

1) Continual background emissions in normal engine operation, as engine oil seals are not an absolute design.
2) Transient low-level leakage in normal operation with engine power and air supply configuration changes.
3) Less frequent higher-level exposures with certain operational factors or partial or full failure conditions.

The use of engine compressor-generated pressurised air to both seal the oil-bearing chamber and to provide ventilation air for the cabin guarantees that fugitive low-level oil emissions will enter the air supply during normal engine operation (57).

Although contaminants linked to the ventilation air supply may come from a variety of sources, as indicated in Appendices 1A and 1B (55, 56), there has been a particular focus on the oil and hydraulic fumes over many years as highlighted in 2015 by the International Civil Aviation Organization (ICAO): *"Of all of these potential contaminants in the cabin and flight deck, particular concerns have been raised regarding the negative impact on flight safety when crew members are exposed to oil or hydraulic fluid fumes or smoke, and experience acute symptoms in flight"* (55). With regard to oil seals, "*It is accepted that such seals will leak a very small amount of oil vapor during normal service*" (57). The oil leakage due to engine operating conditions "*pollutes the cabin/cockpit air"* (58).

There are two main ways in which exposures may occur (59):

1) Acute high-dose fume events in which there is generally only a detectable odour without a visible haze. This can range from the more common transient lower concentration event during normal operations to the less frequent higher-level event occurring during abnormal operations. Events with symptoms may occur in the absence of an identifiable odour or mist.

2) Chronic repeated low-dose exposure of aircrew on a continual basis to a complex mixture of fugitive jet engine oil emissions. In this scenario most aircrew/persons would not suffer symptoms, but some may.

Fume events:

Fume events are described in a variety of ways. Most have no visual identifying features, such as mist or smoke. Oil fumes are typically described as smelling like dirty socks/smelly feet, foul, musty or oily odours, while hydraulic fluid is often described as acrid (55). The dirty socks or smelly feet description, often used, is increasingly understood to be related to the thermal degradation and hydrolysis of the oil base stocks creating carboxylic acids (60-62). Odour is subjective, with different people describing the same fumes differently, while olfactory fatigue reduces a person's ability to detect odours over time (55). After three minutes of exposure to an odorant, the perceived intensity of the odorant is reduced by about 75% (63). Additionally, fumes have often been regarded as a nuisance, with education and training to increase awareness and reporting having only recently been promulgated (55). In some cases, there may be no detectable odour, however, symptoms characteristic of a fume event may be reported at specific stages of flight.

There are neither detection nor warning systems on board aircraft to advise the crew and maintenance staff when the air is or has been contaminated. Furthermore, there are difficulties in identifying the source of fume events during maintenance practices, particularly for the more common lower-level transient events (64-68).

Hazard classifications:

Aircraft oils and fluids are known to contain hazardous substances and additives, including organophosphates (OPs) (28, 69-74). The hazard classifications related to these fluids listed under the Globally Harmonized System of Classification and Labelling of Chemicals (GHS) (75) (a system established by the United Nations to provide information on hazardous products) include: toxic for reproduction; may damage fertility or the unborn child; suspected of causing cancer; harmful by dermal exposure; specific target organ toxicity – single and repeat exposure; neurotoxicity; eye and skin irritant; skin sensitisation; may cause allergy or asthma or breathing difficulties if inhaled; irritation of the respiratory tract; and, may cause drowsiness or dizziness (28, 73, 74).

Oil and hydraulic fluid compounds:

Tricresyl phosphate (TCP) is used as an antiwear additive in most engine oils at 2.5-5% concentration. While there has often been a focus on one isomer of TCP, tri-ortho-cresyl phosphate (T*o*CP), there are nine other ortho, meta and para isomers in TCP. The commercial formulations of TCP used in engine lubricants contain a wide range of cresols, phenols and

xylenols (76, 77), including trixylyl phosphate (TXP). Isopropylated phenyl phosphates (3:1) [TIPP/PIP (3:1)] are used in at least one brand of oil rather than TCP and in selected hydraulic fluids. Tributyl phosphate (TBP) is a constituent of most (but not all) hydraulic fluids, ranging from 20-80%, and some oils and hydraulic fluids contain triphenyl phosphate. Amines, such phenyl-α-naphthylamine (PAN) or alkylated diphenylamines are used as antioxidants in a concentration of about 1% in the oils. Beta naphthylamine (BNA), a category 1A carcinogen, is a contaminant of PAN. However, BNA is reported to present at very low levels in the oils and, therefore, not listed on the Material Safety Data Sheets (MSDS) (78). Upon exposure to the very high temperature of the engine, the oil and other fluids are known to generate a thermally degraded or pyrolysed[1] complex mixture of substances (61, 70, 78-81). Therefore, there is exposure to a complex mixture of volatile and semi-volatile hydrocarbons and their degradation products, many of which are known to be hazardous to health.

Complex mixture:

The complex mixture of jet engine oils and other aircraft fluids have been documented in a variety of studies. However, the approaches undertaken have rarely been systematic nor widely available for review. The contaminants that could be in the aircraft cabin environment relating to oils, fuel, hydraulic and de-icing fluids, anti-corrosion coatings, amongst others, are categorised by the American Society of Heating, Refrigerating and Air-Conditioning Engineers (ASHRAE) as shown in Appendix 1B (60). According to ASHRAE (60), carbon monoxide (CO), ultrafine particles (UFP) (which have diameter of 0.1 µm or less), particulate matter less than 2.5 micrometres in diameter ($PM_{2.5}$), aldehydes, OPs, alkanes, amines and esters are consistent with oil and hydraulic fluid contaminants. Carboxylic acids, aromatics and ketones are also linked to oil. Fuel is associated with CO, UFPs, aldehydes, aromatics, alkanes and alcohols. UFP measurements of pyrolysed oil found that *"contamination in the compressor will result in a fog of very fine droplets in the bleed air under most operating conditions"* (82).

Air quality studies:

Substances belonging to the categories listed above, have been repeatedly identified in cabin air

[1]. The term pyrolysis has been widely used in association with oils heated to high temperatures within aircraft engines. However, the correct terminology is now recognised to be thermolysis or thermal degradation given that the oils or hydraulic fluids are heated to high temperatures in the presence of air (ASHRAE, 2022). The term pyrolysis will still be used in this report given its wide use, however it is used here to represent thermolysis or thermal degradation.

quality studies (66, 83-88), studies specifically related to the aircraft bleed air supply system in oil contamination (61, 81, 82, 89-93), and oil pyrolysis studies (80, 94-97). The aircraft cabin and bleed air ad hoc studies have generally not been undertaken during fume events. However, in one instance a series of air samples were collected during normal flight in which mild fumes were noticed by some people. TCP and TBP were identified in most of these in most of the samples (88). Sixty-four of the most frequently occurring compounds measured in bleed air are listed in Appendix 1C (89). Ritchie et al. (98) identified kerosene-based jet fuels as an additional source of hydrocarbons. Other contaminants include those related to the black carbonaceous material adhered to the ducting in the air supply, which can contain aluminium, silicon, sulphur and phosphorous that could be released into the cabin air under certain operational conditions (61, 99). These carbonaceous materials and associated chemicals were identified as 'entirely consistent' with the products of pyrolysed aircraft engine oils (61). Additionally, one function of the oil is to wash away very small particles such as metal released from the bearings, seals and gears during normal operation (100). Used engine oil evaluation provides further details on the microscopic wear of metals within the engine and oil system (101, 102).

No detection systems specific to oil or hydraulic fluid fumes exist to monitor contaminant levels in the aircraft cabin during a fume event. Available data on potential contaminants present are obtained from a small number of industry sponsored cabin air quality studies, or occasionally from maintenance studies undertaken hours or days after the event (see references above) as described in Chen et al., (87). Levels of contaminants identified in these ad hoc studies are generally reported to be well below occupational exposure limits (OEL). Air monitoring undertaken in Australia when fumes generally considered as 'normal' were present, identified low levels of TCP and TBP in most samples, with a pilot medical disqualification noted after repeatedly identifying fumes and adverse effects (88).

Occupational exposure limits:

Occupational exposure limits or threshold limit values (TLV) are applicable to individual substances but not to all substances. The limits are set to protect most, but not all, workers handling hazardous substances under a specified occupational health and safety condition in the workplace. The limits are not applicable to the general population, which includes pregnant women and children. OELs are not a fine line between safe and dangerous exposures and do not exclude the development of occupationally induced illness. The application of conventional

occupational health and safety procedures to the specialised aircraft environment "*are inappropriate*" (70) and such threshold limits are not applicable to *"complex mixtures with many components (e.g., gasoline, diesel exhaust, thermal decomposition products... etc.)"* (103).

Individual classical toxicology limits will not be protective for exposure to complex mixtures, such as oil fumes, and especially for aircrew exposed to repeated low doses (57). There is longstanding toxicological knowledge that, with regard to risk assessment, it should be borne in mind that exposure to various substances could occur within a short period of time and, as a result of these mixed loads, an additive or potentiated effect may occur, which does not meet the consideration of the toxicity of the individual substances (104). As stated in an EASA/EU funded pyrolysed oil study, *"the conditions in cabin air may differ from standard conditions on which exposure limits are normally based"* (80).

Additionally, exposure limits may not reflect the physiological effects of altitude that will be present in an aircraft cabin during flight (105, 106). Environmental changes that arise from altitude include reductions in humidity, temperature and atmospheric pressure and an increase in radiation (particularly over the polar areas). These environmental changes may lead to changes in pulmonary physiology and reduction in oxygenation. The clinical effect of the intake of hazardous substances in an aircraft cabin at altitude has not been well defined, especially in children (107). *"No physiological effects due to oxygen deficiency are expected in healthy adults at oxygen partial pressures greater than 132 torr or at elevations less than 5000 feet"* (103). At oxygen partial pressures less than 120 torr (5000-7000 feet), *"symptoms in unacclimatized workers include increased pulmonary ventilation and cardiac output, incoordination and impaired attention and thinking. "These symptoms are recognized as being incompatible with safe performance of duties)"* (103).

AVIATION INDUSTRY SCIENTIFIC INITIATIVES RELATED TO FUME EVENTS

Although records of aircraft fume events are available dating back to the 1950s, there has been a noticeable increase in aviation industry and scientific investigations of fume exposures since the 1990s.

Reviews of oil fume exposures:

There have been various reviews into new and used engine oils. Reviews have suggested that: it would be almost impossible for a person in an aircraft to receive enough jet oil to cause organophosphate-induced delayed peripheral neuropathy (OPIDN) (77); no toxic effects could be expected, but symptoms of irritation could occur due to pyrolysis of the oils (61); and that pyrolysis of the oils generated 127 different compounds and that there were gaps in knowledge with regards to the effects of exposure to the oils and pyrolysis products (80).

Air monitoring studies:

Air monitoring studies have reported that the concentration levels of the volatile organic compounds (VOCs) identified were similar to other indoor environments (66) that no pollutants in the cabin exceeded available health and safety standards and guidelines (83), and that typical concentrations of VOCs found in the cabin can cause transitory symptoms in healthy individuals (therefore questioning the adequacy of current standards) (108).

Health risk assessments:

Limited health risk assessments and reviews have been undertaken that report that T*o*CP exposure cannot be the cause of Aerotoxic Syndrome (109), patterns of illness do not conform to OPIDN and ortho-TCP exposures (110) and or that the pattern of clinical signs observed are serious enough to warrant a call for further elucidation (111).

TCP/oil pyrolysis studies:

Limited studies of TCP include *in vitro* analysis showing impairment of glutamate sensitivity of neurons at very low T*o*CP levels (112); rat ingestion of a blend of TCP used in engine oils significantly impaired various enzymes (113) and *in vitro* neurotoxicity studies of various TCP isomers (including non-ortho) found that the different TCP isomers were roughly equipotent and that prolonged or repeated exposure to TCP may exacerbate the observed neurotoxic effects (114). An *in vitro* neurotoxicity assessment of exposure to pyrolysed engine oils found that

neuroactive pyrolysis products were present (80). The study found a modest trend towards increased neuronal activity when the fumes at higher concentrations were passed through an *in vitro* lung model. When not passed through the lung cells, neuronal activity was significantly increased on an acute basis, and (80) prolonged exposure (48 hours+) markedly decreased neuronal activity was reported raising the need to focus on prolonged and or repeated exposure to pyrolysis products (80). A further in vitro study exposing lung models to simulated pyrolysed oil and hydraulic fumes reported that *"exposure to engine oil and hydraulic fluid fumes can induce considerable lung toxicity, clearly reflecting the potential health risks of contaminated aircraft cabin air"* (115).

Government and regulator actions and studies:

In 2016 the European Commission and the European aviation regulator outlined a four-stage pathway into the investigation of fume events, effect on people and risk mitigation strategies (116). The study was delayed, with a final report released in late 2021, which identified the study was incomplete, with limited results provided only including those identified by He et al., (115-117). Given the limitations of the study and the inability to answer "the ever evolving questions regarding potential health effects", further research has been planned (118, 119). A bill was passed into US law under the Federal Aviation Administration 2018 Reauthorization Act (120). The areas addressed included assessment of the health effects on passengers and aircrew exposed to aircraft bleed air constituents, but this research has not taken place to date.

Care pathways:

A fume event guidance document for health care professionals was published in 2009, however, while good, this was not as specific as presented here and not widely utilised (29). The International Air Transport Association (IATA) published guidance for airline health and safety staff on the medical response to cabin air quality events (51). Limited investigations are outlined for 'high risk exposures' with neurological and mini mental state examinations reported to be 'rarely' required. The UK Civil Aviation Authority (CAA) and National Health Service (NHS) have published an NHS care pathway guideline on how to respond to people exposed to fumes in aircraft (52). The guidelines are not specific and do not provide any suggested investigations. Some airlines and manufacturers are reported to have outlined limited tests that could be undertaken after fume events. The German Social Accident Insurer has published some limited medical procedure guidance for fume events (121). There have been other independent calls for a

defined medical protocol to be developed (28, 49, 50). More recently the CEN European Committee for Standardization has issued a Technical Report which recommends medical monitoring at the commencement of aircrew employment and for aircrew and passengers after fume events using a best practice medical protocol (122).

CLINICAL EFFECTS DESCRIBED BY PEOPLE AFTER AN AIRCRAFT FUME EVENT

General:

The medical presentations of people who have been exposed to and inhaled fumes from jet engine oil, hydraulic and other fluids via the air supplied to the aircraft cabin, are variable and well described in the literature (10-43, 45-48, 50, 57, 59, 88, 123-126). In summary, symptoms initially, and consistently, are described as foggy thinking, dizziness, recognising an odour in the cabin (commonly described as a 'dirty socks' smell), impaired short-term memory and cognitive thinking, fatigue, headache, nausea, tremor, balance, incoordination, breathing difficulties, chest pain, eye, nose and throat irritation. Many other symptoms, with a delayed onset, have also been reported, but the common factor that binds both the acute and delayed complaints is that they are all consistent with volatile organic hydrocarbon and organophosphate toxicity. The regularity and consistency of presentation demands that this constellation of symptoms deserves identifying nomenclature as previously discussed.

Aerotoxic Syndrome:

As shown in Appendix 2, Aerotoxic Syndrome encompasses a constellation of symptoms and, as is common in many medical conditions with the 'Syndrome' label, the complete list of symptoms and clinical findings is not necessarily found in any individual case. In this regard, it is clear from the experience of medical personnel managing cases of fume event exposures over many years and the accumulated reports in the peer reviewed literature that there is considerable individual susceptibility and variation in symptoms (28). It is likely that this observation is based on various factors, particularly environmental factors, such as the intensity and duration of exposure, exposure conditions, repeated exposures over time versus a single exposure and probably, the duration of the individual's service in the industry. Also, clinical comorbidities, lifestyle factors (diet, smoking and alcohol use), age, general health, concurrent medication, genetic susceptibility and reproductive status may play a role. In this regard, it is germane to note that it appears to be the total accumulated dose over time that is a key factor. In short, symptoms may be prompted by a single contamination or repeated low-level exposures. It has been suggested that crew members are the most susceptible to the Aerotoxic Syndrome (28, 59, 99, 127).

Symptoms of Aerotoxic Syndrome are recognised to be non-specific and diffuse and, as such, may be dismissed by a clinician who only sees a single or only occasional case (28, 57, 59). However, at

the epidemiological or population level, a syndrome can be identified (28). Symptoms and signs are variable and may affect any organ system. There is wide variability between individuals in their capacity to metabolise and detoxify OP compounds (59). The highly variable individual susceptibility to damage by OP exposure is exemplified and has been reported in farmers exposed to OP-containing sheep dips. A study involving individuals who dipped sheep for their job found that those with lower concentrations of liver and plasma enzymes that detoxify OPs were more likely to suffer from 'dipper's flu' (128).

Aircrew, aviation workers and frequent fliers appear to be more susceptible to Aerotoxic Syndrome than individuals who do not fly often (59). However, some passengers who fly infrequently may fall into the susceptible category for reasons set out above.

Misdiagnosis:

Experience has shown that people suffering from the effects of aircraft fume events are commonly misunderstood and misdiagnosed as being anxious, stressed or experiencing other clinical complaints. Misdiagnosis occurs because the toxic effects of pyrolysed aircraft engine oil are not widely recognised by health care professionals, including airline medical staff, when first consulted by the affected crew members or passengers. Even when the exposure is raised as the cause of their symptoms by the afflicted person, there is frequently a general view that the exposure was not significant enough to cause toxic symptoms. In this regard, health care professionals need to be aware that it is well recognised that toxicity and injury can occur following exposures well below industry accepted standards and that the absence of this form of toxicity from standard reference material does not mean that it does not occur. As in the case of multiple sclerosis, the presenting symptoms take many different forms.

Collection of data:

Research highlighting adverse effects and outlining possible diagnostic criteria for symptomatic people after cabin air fume events, has led to calls for a clear medical investigation protocol (28, 49, 50). Repeated reports to a poisons unit of noxious fumes released into the aircraft cabin, has led to a call for the aviation regulator to require better safeguards to be put in place to prevent 'Aerotoxic Syndrome' (125). Similar calls have been made by public health agencies for better procedures, understanding and collection of data (126).

The need for a well-defined, internationally recognised medical protocol to be used in the management and investigation of people who have been exposed to fumes in an aircraft cabin is therefore provided here. It includes not only clinical information, but also the necessary data to record about the environment in which the event occurred – the what, when, where and how. Only in collecting information in a systematic way can problems associated with fume events be properly addressed with the safety, health and well-being of all people who fly in mind. A clear medical investigation protocol would also protect manufacturers and the airline industry.

PROPOSAL FOR THE DOCUMENTATION OF A FUME EVENT, CLINICAL HISTORY AND PHYSICAL EXAMINATION

General:

As outlined above, in all reported cases of fume event exposures, or Aerotoxic Syndrome, the onset or continuation of symptoms may follow one of several different time patterns as follows:

- In-flight.
- Immediate post-flight (within one to two days).
- Late/subsequent (beyond two days).

Most individuals report that the onset of symptoms is time correlated with a flight or immediately after, in some cases necessitating medical attention.

As previously discussed, the clinical symptoms reported can differ between individuals and are heterogeneous; symptoms may include functional failure in organs or organ systems, to a greater or lesser degree, as identified in Appendix 2 (28). Typical short and long-term symptoms have been reported elsewhere (29) and are shown in Appendix 3 (27).

Experience over the past decade or so suggests that in many cases symptoms are of short duration, but may take hours, days or weeks to resolve. In some cases, particularly people who have experienced more than one fume event exposure, symptoms can continue for months or years and occasionally full recovery never occurs. A systematic examination of the correlation between the intensity, duration, frequency of exposures, the effect of cumulative exposure over prolonged periods and ill-health has yet to be evaluated and published. It is anticipated that by adopting the medical protocol described in this report on a global basis that data will be accessible to address unanswered questions.

Collation of data:

The personal information, physiological and pathological data relating to fume events that should be collected and recorded are outlined below. These recommendations are based on the authors' broad and extensive collective experience in managing flight crew who have been exposed to a fume event of any type and who have developed any of the wide variety of symptoms that have

been reported to be associated with the cabin air incidents/Aerotoxic Syndrome (10, 13, 15-19, 21-23, 26-29, 32-43, 45, 46, 125, 129).

Undertaking all of the examinations and special investigations suggested in this protocol may not be possible or medically indicated in every case. Some investigations require specialist laboratories, and there will be practical issues of availability, timing and cost for procedures and tests. Requests for a special investigation should be based on clinical indication in each individual case. Despite these caveats, it is strongly recommended that as much data is collected as is practically possible in every case. Our recommendations for data collection, medical examination and special investigations are set below and formatted to examine each organ system dysfunction individually and are further broken down into time of presentation (In-flight; Immediate post-flight; and Late/subsequent).

Fume events, as discussed in this protocol, are associated with compounds in a gaseous/vapour or particulate/aerosol phase, generally with an odour and are not visible. However, this is not always the case and infrequently a visible mist or smoke may become evident and, less frequently, there may be no detectable odour. Fume events are more commonly cited as being of short duration but sustained more noticeable events are also reported. Some people may be symptomatic to varying degrees, while others may remain symptom-free or experience a delayed onset.

Hospital accident and emergency departments have standard protocols and/or medical triage procedures. The methods described in the medical protocol outlined in this report are, for the most part, based on presenting symptoms, clinical signs or other concerns, such as symptom severity (irritant only or otherwise). Thus, useful information for the ongoing care of the affected individual may not be gathered in the busy milieu of primary care or emergency facilities.

Only with a comprehensive approach in the context of a fume event presentation will this issue be solved. Given that this protocol addresses fume events related to aircraft design in both normal and abnormal operations, the authors hold that a more comprehensive review should be considered, including a toxicological assessment independent of symptomatic presentation alone.

Assessments:

Initial medical assessments undertaken at the completion of flight duties may also be required for asymptomatic, or relatively asymptomatic, people who have been exposed to fumes in an aircraft cabin. This needs to be considered when decisions are made on an initial medical management approach. Such persons also may be important in the conduct of clinical and epidemiological studies in order to address why some people react poorly to fumes in the aircraft cabin while others do not.

The recommended investigation and management approach suggested upon presentation to a medical facility is outlined below and, in the following flowchart (see Figure 1).

1. In-flight assessment ----> Section 1A.

Recommended to be undertaken for all people exposed to a fume event on an aircraft (in flight/on ground) as appropriate.

2. Immediate post-flight/event ----> Section 1B.

- All occupants:
    - Symptomatic (crew or passengers) – Full medical assessment strongly recommended (1B a, b, c).
    - Asymptomatic (crew or passengers) – Offer medical assessment: brief or full if concerned (strongly recommended for crew members).

Section 1 relates to the general investigations that should be considered after a fume event. Section 2 provides further details on human biomonitoring. Sections 3 to 11 provide relevant information and investigations for specialist areas.

The decision to what extent medical assessment should be undertaken after a fume event, should take into account a variety of factors including: potential exposure to hazardous substances and the duty of care, symptoms may arise at the time of the event, soon after, or on a latent basis. We recommend collecting the data at the time of the incident or at first presentation.

Crew should not be cleared back to fly without an assessment.

In Appendix 4, we summarise our collective experiences using each of the time pattern headings identified above. Appendix 4 has been designed to guide fume-affected people, those who assist them and medical staff who are subsequently consulted. Appendix 5 may provide assistance with regard to documenting symptoms and timing after a fume event.

Figure 1: Recommended investigation and management approach for persons exposed to fume events

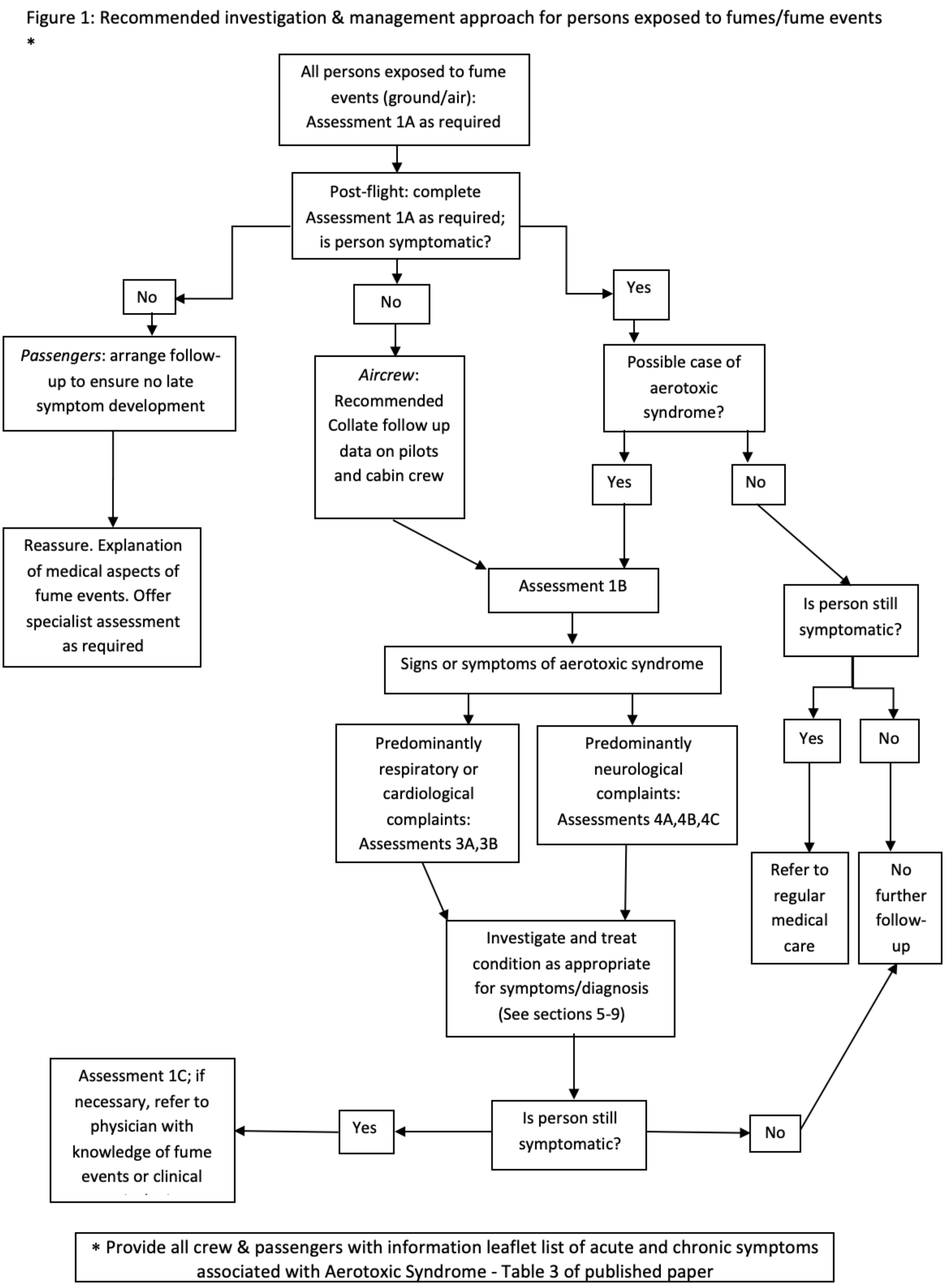

SECTION 1: POST-EVENT MEDICAL PROTOCOL

Section 1A. In flight (ground or air):

May be undertaken by non-medically trained personnel.

Summary

- **Environment observations (technical record of the fume event).**
- **First aid.**
- **History of the symptoms of affected people and measures taken related to the fume event.**
- **Physical examination (if possible).**

Details

A detailed record of the fume event, with details of technical and engineering follow-up, together with a record of the symptoms and the medical management of people who have been exposed are indispensable for longer-term medical management. Detailed records help to plan an ongoing bio-monitoring strategy and objectively correlate symptoms and functional disorders in organ systems. If medical help is available, collect and record data listed under 1B below. The level of detail recorded will depend on the extent and degree of adverse health effects experienced.

The following in-flight report is recommended:

Environmental observations

(Record as follows)

- Type of aircraft.
- When did the event occur (in-flight, stage of flight, on ground, ascent, descent)?
- Where in the aircraft did the event occur?
- For how long did the event continue?
- What happened (e.g. odour, fumes, smoke)?
- If odorous fumes, describe.
- Who and how many (x out of y) was/were affected, when and for how long (aircrew, passengers)?

- Record of air quality monitor recordings (if available)/maintenance history/previous events if known.
- First aid response.

(Record to be made by those assisting people who have been affected by the fume event (crew members and passengers, for example).

History of symptoms and measures related to the fume event

(Record as follows)

- A detailed and carefully documented description and severity of the fume event experienced by the individual.
- Record symptoms and progression of symptoms.
- Record observations of others, important in assessment of affected persons.
- Record any treatment given/used.
- Record any treatments for past exposures, if known.
- Measure and record oximetry, if available, before oxygen administration.
- Record whether oxygen was used (including flow rate, method of administration (for example, nasal cannulae/mask, when and duration).
- Record any unusual behaviour.
- If possible, record pre-existing health complaints/disorders/findings/medication.
- Record other possible diagnoses (to be considered for differential diagnosis purposes).

Physical examination

Trained healthcare professionals may not be present to conduct a medical examination. However, observations of physical findings or behaviours should be recorded because they are helpful to future medical providers in their initial assessment and for guiding ongoing medical management.

If a trained health care professional (for example, a doctor, nurse or paramedic) is present, then a physical examination as listed in Section 1B 'Immediate post-flight' (see below) is strongly recommended.

Section 1B. Immediate post-flight/event:

Summary

- **Go to the emergency room that is closest to the airport**
    - Medical history of the event.
    - Clinical examination.
    - General investigations - Collect samples (blood/urine – see below) for human biomonitoring as soon as possible after a fume event.
    - Recommend transfer to medical specialists for timely further clarification/investigation (as required).

Details

Record a medical history of the event and carry out a thorough clinical examination.

a. Medical history of event

- A detailed and careful occupational history of the fume event, including timing, severity and duration of the fume event. Also record the frequency, duration and intensity of previous fume exposures (see Environmental Observations, in Section 1A, above).
- Record total flying hours (Pilots will know this from their logbooks. Cabin crew can estimate total hours from contracted annual hours x length of service less time for absences such as annual and sick leave, part-time work and maternity leave).
- Record symptoms and progression of symptoms, observations made by other people such as crew members and passengers (important in assessment of affected persons), any treatment given/used, whether oxygen was used and when/duration including flow rate (in-flight or post-flight) and unusual behaviour, as outlined in the section: Medical history of event, above (see Section 1A, above).

b. Clinical examination (symptom related)

- Record general appearance (for example, breathlessness, pallor, agitation).
- Measure and record respiratory and heart rate, blood pressure.
- Auscultation of heart and lungs.
- General physical examination.
- Record use of oxygen and when applied.

- Record oxygen saturation (SpO_2 -– if an oximeter is available, record whether supplemental oxygen was used).
- Monitor SpO_2, if initial SpO_2 <95%.
- Assess neurological status (conscious state, balance, muscle weakness, numbness, pupils, muscle reflexes, check for tingling of limbs, muscle cramps, tremor).
- Assessment using the Mini-Mental State Examination MMSE: (Orientation for time and place; attention and calculation; memory and processing speed).
- Other findings.

c. General investigations

General investigations should be undertaken as soon as possible following a fume event, but should ideally be within two to four hours and three days to complement the above clinical examination and may include:

(i) Routinely available:

- Full blood examination (Hb, WCC and differential count).
- Acute phase reactants (e.g., C-reactive protein, ESR, fibrinogen).
- Routine biochemistry (U&E/Cr, LFTs, LDH).
- Muscle enzymes (e.g., troponin, CKMM and CKMB, aldolase);
- Bloods for cholinesterases – (AChE, BChE)[a] see below for details and Section 2.
- Others, as clinically indicated.
- Carboxyhaemoglobin – HbCO (should be undertaken within two hours post flight to a maximum four hours post flight for accurate measurements due to short half-life). Record time since exposure and/or time of last cigarette. (Indication of CO intoxication).
- Methaemoglobin (should be undertaken within two to four hours post flight for best assessment due to short half-life).
- Neurobehavioural: basic quick (5 min) testing of processing speed using the Symbol Digit Modalities test (SDMT) (oral and written) and/or digit span forwards and backwards is recommended initially, followed by early referral for more detailed neuropsychological testing if required (refer to section 4C).

(ii) Non-routinely available

- Blood for neuropathy target esterase (NTE)[a,b] – see below for details and Section 2.
- Urine for OPs[a,b] – As soon as possible after a fume event: see below for details, Section 2, Appendices 6 and 7.
- Blood for VOCs[b] – As soon as possible after a fume event: see below for details, Section 2, Appendices 6 and 7.

Footnotes:

a. Level of OP exposure may not be high enough to show enzyme inhibition or TCP urinary metabolites. Lack of inhibition or metabolites does not indicate that OP exposure did not take place.

b. Testing is not routinely available and requires specialist laboratories.

Note that a formal chain of custody should be considered for all samples.

(iii) Other tests as clinically required

See sections:

- Respiratory/heart - Section 3
- Neurological - Section 4
- Neurobehavioral - Section 4
- Irritants - Section 5
- Sensitisation – Section 6
- Skin – Section 7
- Gastrointestinal - Section 8
- Other - Section 9

(iv) Blood and urine sampling – additional information

Bloods may be taken to assess cholinesterase and, where possible, NTE levels. Details are provided below. Cholinesterase inhibition can be assessed in two ways (enzyme activity or mass spectroscopy). At present, the enzyme activity method is the only one that can be readily assessed, while the mass spectroscopy method must be stored locally until the mass spectroscopy assessments can be undertaken. The sampling method is set out in Table 1, below. It is important to follow this outline carefully, taking into account the clinical presentation of the person, cost and practicality of testing.

Urine samples could be taken to assess for specific OPs, and blood samples could be used to assess for VOCs (see Appendix 6). At present, the sampling methods for the OPs and VOCs are very specific, costly and require organisation with specialist laboratories in advance to ensure the process is undertaken appropriately. While this sampling will not be currently widely available, further details are provided in Appendix 6 to assist those wishing to undertake this form of sampling. However, in this protocol, the data will be presented for completeness, but it is unlikely that this testing can be widely undertaken at present.

Blood analysis for acetylcholinesterase, butyrylcholinesterase and neuropathy target esterase

(See Table 1, below)

Acetylcholinesterase - Butyrylcholinesterase - Cholinesterase enzyme assay

- Red blood cell (RBC) cholinesterase – (acetylcholinesterase – AChE).
- Plasma cholinesterase – (butyrylcholinesterase – BChE).

The AChE bound to the erythrocytes (red blood cells) correlates with the AChE activity in the neurones. Reduction of AChE activity in isolated erythrocytes may be between 30% to 70% of the individual reference value (baseline). After reaction with OPs the esterase activity mainly recovers within a period of several weeks after new synthesis. Measurement of red cell AChE and plasma BChE activity can be undertaken by standard activity assays or using a ChE Check Mobile Test Kit (130).

Blood analysis should ideally be carried out at laboratories with established methods and experience with the required assays. Initial blood samples are to be taken preferably between 4–24 hours following the fume event for BChE, and 4–48 hours for AChE. A baseline is required to be taken three months after the initial sample or when symptoms stabilise and at least one week before return to the work environment. Ideally, a person should define their baseline activity levels before starting to fly.

Note: Level of OP exposure may not be high enough to show AChE / BChE enzyme inhibition. Lack of inhibition does not mean OP exposure did not take place.

Acetylcholinesterase, butyrylcholinesterase and cholinesterase mass spectrometry

Cholinesterase sample analysis with mass spectroscopic blood analysis for triaryl phosphate (TAP) biomarker research is currently being undertaken at the University of Washington in the United States. The University of Washington cannot accept samples at this stage. However, blood can be stored locally and forwarded to the University of Washington when advised. The following protocol for obtaining blood samples from a person exposed to a fume event to be analysed by mass spectroscopy is as follows: Collect 4 x 6 ml of blood in EDTA tubes, taken not less than 4 hours and not greater than one week (for BChE) or two weeks (for AChE) following the fume event. Separate (centrifuge) plasma and red cells and store separately at -20°C to –80°C locally. A single sample only is required. See Section 2A for further information.

Neuropathy target esterase

The neuropathy target esterase (NTE) activity in the nervous tissue is correlated with that in lymphocytes. Research based on animal studies suggests that the irreversible inhibition of NTE in the nervous tissue may be the first indicator of the onset of organophosphate-induced delayed peripheral neuropathy (OPIDN). AChE and NTE are different enzymes that serve as biomarkers of exposure to many OP compounds. There is no connection between AChE inhibition and NTE inhibition. Reference values for the NTE activities were 3.01–24.0 nmol phenyl valerate / (min/mg protein) (131).

Note: Level of OP exposure may not be high enough to show NTE enzyme inhibition. Lack of inhibition does not mean OP exposure did not take place.

Table 1: Blood sampling for acetylcholinesterase (AChE), butyrylcholinesterase (BChE) and neuropathy target esterase (NTE).

Method	Enzyme	Half-life (days)	Sample 1 (time after incident)	Sample 2 (Baseline)	Sample details

				(time after incident)	
Enzyme assay	AChE - Red blood cell (RBC)	33	Preferably 4 - 48 hours	2-3 months [a]	Standard protocol **
	BChE - Plasma	12	Preferably 4 – 24 hours	1-2 months [a]	Standard protocol **
	NTE* (lymphocytic)	5-7		2-3 months [a]	Standard protocol* – Only fresh blood can be analysed [c]
Mass spec analysis	AChE	33	4 hours - 2 weeks	One sample required only [b]	4 x 6ml in EDTA tubes [d]
	BChE	12	4 hours - 1 week	One sample required only [b]	4 x 6ml in EDTA tubes [d]

* NTE blood analysis is not routinely available.

** Baseline AChE and BChE values for OP exposures have been generally determined for agricultural exposures, but not for aircraft fume event exposures. Note that there is a wide variation between individual baseline levels and therefore it is the 30–70% inhibition below the individual baseline that is the important reference. Each laboratory will use differing reference levels. Reference levels do not consider the individual variation, which is the most important factor when analysing biomarkers as an indicator of OP exposure, as outlined above.

Note: Measurement of erythrocytic AChE/BChE activity also available as a ChE Check Mobile Test Kit. (A follow-up baseline activity determination is also required for accuracy.)

Footnotes

a. A second sample to be undertaken as a baseline. AChE recovers to normal level after around two to three months, while BChE recovers after around one to two months. If symptoms alleviate before this time, undertake a baseline sample before returning to work or when away from further exposures. It is preferable to undertake a baseline before starting flying employment.

b. Store locally at present.

c. NTE analysis: Only fresh blood can be used.

d. Separate (centrifuge) plasma and red cells and store separately at -20°C to –80°C locally. Assays preferably tested in triplicate within the same laboratory with 5% range.

Blood analysis: volatile organic compounds (see Section 2 and Appendix 6)

Important note: Not routinely available. Contact a specialist laboratory in advance for advice on storage, shipping and analysis.

- Volatile organic compounds (VOCs): Collect 5 ml normal EDTA Blood (2ml blood samples must be transferred rapidly to coated headspace tubes for gas chromatography–mass spectrometry (GC–MS) analysis). Contact a specialised laboratory that is certified to carry out the required analysis.

Initial samples should be taken as soon as possible after the fume event; follow up samples should be undertaken as described in Appendix 6.

Urine analysis: organophosphates (see Section 2 and Appendix 6)

Important note: Not routinely available. Contact a specialist laboratory in advance for advice on storage, shipping and analysis.

- 20 ml urine collected as described in Appendix 6. Store and ship in accordance with advice from a specialist laboratory. *

Initial samples should be taken as soon as possible after the event; follow-up samples should be undertaken as described in Appendix 6.

Footnote:

**Sampling, analysis and shipping arrangements should be discussed in advance with a specialist laboratory. The collection tubes are normally provided by the laboratory. The samples can be sent to the laboratory using special human sample bags (coated with plastic). It is possible to send 2 x 10ml tightly closed urine samples in the human sample bag via regular post. The samples are sent at room temperature as quickly as possible after sampling and then cooled in the laboratory.*

Note: Level of OP exposure may not be high enough to show TCP urinary metabolites – see Section 2.

Section 1C. Late/subsequent

A late or subsequent presentation relates to first consultations with medical staff that take place a few days, weeks or even months following the fume event. The medical approach is not dissimilar to that recommended for earlier presentations, in that a detailed clinical history of the events and symptoms experienced at the time of the fume event and those since need to be recorded and a formal physical examination pertinent to the presenting symptomatology undertaken.

The recommendations below should be taken as a guide:

Medical history of event

- As for Immediate post-flight (see Section 1B, above).

Clinical examination

- As for Immediate post-flight (see Section 1B, above).
- Referral for specialist consultation should be considered as appropriate.

Further discussion regarding special investigations appears in the sections below. It is important to understand that in some cases it will be necessary to undertake the investigation in all people who have been affected by a fume event, while in other situations an investigation may be undertaken based on clinical indication. In general terms, it is always important to consider whether a test undertaken will assist in diagnosis or management. Negative tests are as useful, in many circumstances, as tests that are positive. Cost and availability may need to be considered in some cases.

Many sources of information are available relating to the fluids, substances in the products or pyrolysis products in the aircraft ventilation air supply and bleed air. Some examples include phenyl-α-naphthylamine (PAN) *"may cause effects on the blood, resulting in formation of methaemoglobin. The effects may be delayed"* (132). Carbon monoxide may be formed in association with the pyrolysed mixture of the fluids, depending on temperatures. An oil industry material safety data sheet states: *"product may decompose at elevated temperatures… And give off irritating and/or harmful (carbon monoxide) gases/vapors/fumes"* (133).

SECTION 2: FURTHER INFORMATION ON HUMAN BIOMONITORING

Section 2A. Immediate post-flight/event

Because each hazardous substance and each biomarker protein have different half-lives, the timing for urine and blood collection will differ as set out in Table 1, Appendix 6 and as shown below. Understanding the half-lives will aid in calculating the enzyme activity or levels of the excretion gradient. Both effect-monitoring and human-biomonitoring can be undertaken.

Effect-monitoring: acetylcholinesterase, butyrylcholinesterase, neuropathy target esterase - General

Organophosphates (OPs) are regarded as one of the most likely causes of symptoms following a fume event. There are many types of esterases and other serine active site hydrolases, proteins that metabolise a host of biological compounds (for example, lipids, proteins and physiological metabolites). These proteins are potential biomarkers of OP exposure. Acetylcholinesterase (AChE) is commonly referenced when addressing health effects of OP exposures because inhibition of AChE has serious consequences from neurological damage to death. However, as described below, there is a wide variability in normal AChE and butyrylcholinesterase (BChE) levels in humans, complicating protocols that measure cholinesterase activities. Solutions to this issue follow.

There are two methods to assess inhibition of cholinesterases. First, the more common historical approach is to assess the enzyme activity level using specific assays. Limitations of the enzyme activity assay are that activity levels are recognised to vary somewhat from day-to-day, between laboratories and much more between individuals (134), so single time point assays provide inadequate information. Establishment of an individual's baseline activity level prior to or some time following exposure is important, followed by further recordings to track any changes as outlined previously. The second more complex but more accurate method is to assess the percentage modification (or inhibition) of AChE, BChE or other esterases using mass spectrometry as indicated below. Mass spectroscopy determination of cholinesterase modification correlates well with activity inhibition levels and avoids the issue of inter-individual variability and interlaboratory variability.

Note: OP levels of exposure may not be high enough to show enzyme inhibition. Enzyme Inhibition requires high exposure or individual susceptibility factors. A negative test result does not equal no exposure.

Effect-monitoring: Triaryl phosphate organophosphate biomarkers

Mass spectroscopy analysis of triaryl phosphate biomarkers

The ortho isomers of tricresyl phosphate (TCP), a triaryl phosphate (TAP) used in anti-wear lubricant additives, are converted by the cytochromes p450 in brain, liver and other tissues into potent inhibitors of serine site enzymes including NTE, which has long been known to be associated with serious neurological damage. While the meta and para isomers of TCP, T*m*CP and T*p*CP, are converted by the P450 enzymes to metabolites that inhibit various enzymes and can cause some demyelination of neurons (135), they have not been reported to cause paralysis. Use of paralysis as a measure of safety for TAP is not a useful endpoint because other damage can occur without the onset of paralysis. Some proteins may be inhibited directly by the TAPs without bioactivation (136).

The University of Washington is undertaking a research study into biomarker proteins modified by TAP exposure (113, 137). The study uses *in vitro* mass spectrometric assays to measure the percentage modification of the active site serines with bioactivated (and non-bioactivated) TAPs, eliminating the need for determining baseline activity levels. The same approach is used as a quick screen for developing less toxic TAPs (113). While the blood test for TAP exposure is still under development, the researchers have suggested that 4 x 6 ml of blood in EDTA tubes, optimally taken not less than four hours and not greater than one week (for BChE) or four weeks (for AChE) after the fume event, may inform the clinician about the percentage inhibition of plasma cholinesterase (BChE) and red cell acetylcholinesterase (AChE). The half-life of BChE in blood is 11-12 days, and red cell AChE is 33 days.

Activity levels of AChE and BChE vary significantly between individuals. Therefore, when using only activity measurements as an indicator of exposure, it is necessary to determine the baseline enzyme activity of the individual prior to exposure or after their system has fully recovered from

the fume event. Baseline measurement of enzyme activity is not required when using mass spectroscopy because this method accurately measures the percentage modification (inhibition) of the enzyme's active site (138).

The following considerations guide these recommendations. Plasma cholinesterase (butyrylcholinesterase or BChE) has a half-life in blood of approximately 12 days (43) and the current limit of detection is about 2% modification of the active site of this enzyme by TAPs. Further, the cresyl group falls off the modified enzyme (aging process) leaving only a phosphate attached to the active site serine of BChE (44). Red cell AChE has a much longer half-life (about 33 days) (139). However, it is less sensitive than BChE to inhibition by TAPs and there are much lower levels of AChE on the red cell membranes than BChE in plasma (140). Unlike with BChE, the cresyl group is not lost through the aging process – it remains attached to the phosphoserine, providing solid evidence that the individual was exposed to a triaryl phosphate (141). The challenges associated with using only the inhibition of cholinesterase activities are noted above and below.

Enzyme activity levels of cholinesterases and other esterases.

Inhibition of the activity of enzymes will vary depending on the substance and there is a known wide range of individual susceptibility, creating the need for determining an individual's baseline activity level. As an example, a variation of AChE activity in isolated erythrocytes from individuals varies by as much as 30–70% compared to the individual baseline reference value (142).

Various studies have been undertaken looking at several of the esterase enzymes related to fume event exposures. Taking into account the limitations of enzyme activity assays as highlighted above and the need for baseline activity, BChE is suggested as a more suitable esterase than AChE to analyse following a fume event that may include OPs (44, 47, 143), with the caveat noted above related to the more accurate mass spectroscopy assays.

Although AChE was shown to be normal in 11 crew members after a fume event, without a baseline (46), BChE was shown to be inhibited in a crew member for up to five days after a fume event (47). However, the inaccuracy of the activity assays in these studies highlights the need for mass spectroscopy analysis. Activity of AChE may be less sensitive to inhibition than BChE.

Nonetheless, mass spectroscopy analysis of modification of AChE, has two important advantages: 1) the cresyl group does not fall off the modified protein after time leaving only a phosphate attached as with BChE; and 2) the half-life of the AChE is much longer at 33 days providing a longer window for analysis following exposures.

Individual AChE and neuropathy target esterase (NTE) activity levels were investigated after fume events with regard to the role of OPs (46). Substances that inhibit AChE or NTE, which include OPs, are used in lubricant additives and flame retardants. As an example, tri-ortho-cresyl phosphate (T*o*CP) does not directly influence serum cholinesterase activity. However, the bioactivated metabolite cresyl saligenin phosphate (CBDP) is a very potent inhibitor of serum cholinesterase activity (113, 144, 145). The measured AChE activities in the preliminary evaluation undertaken at the University for Medicine of Göttingen were usually normal, but these preliminary observations need to be confirmed with further research. The second effect monitoring parameter, NTE activity, clustered at low levels. Values up to 6.3 nmol phenyl valerate / (min/mg protein) were recorded. In the healthy general population, higher levels (up to 24) were observed. These data suggest a likely inhibition of NTE activity in patients after a fume event and indicates a possible exposure to OPs in fume events (46). These data also suggest that a mass spectroscopy analysis of NTE modification by TAPs could be a useful, more accurate assay. In summary methods for generating accurate mass spectrometric measurements for the modification of AChE, BChE and NTE and other serine active site enzymes in affected individuals should be considered as noted above.

Note: In a typical post-fume event investigation, it would not be expected to see NTE inhibited. However, inhibition may occur with a combination of very high exposure and a vulnerable person.

When using activity measurements for determining the percentage inhibition of biomarker enzymes as indicators of exposure, the need for repeat cholinesterase (BChE and AChE) measurements should be considered, noting that it is unlikely that an afflicted person will have had a pre-exposure measurement. Thus, a repeat measurement three months after exposure or at least one week away from the flying environment is sensible to determine whether the initial measurement represents a true change from normal. In this regard, it is important to recognise that an occasional individual will have a normal level well below the recognised ‘normal’ range. For example, one of the control subjects for these studies has a BChE baseline level of only 30%,

which with a single point assay would suggest that they had experienced a serious exposure, however this is their normal activity level. For these reasons, the development of accurate mass spectroscopy methods for determining the percentage modification of biomarker proteins is essential. These methods are amenable to high throughput protocols for mass spectroscopy analyses in some clinical laboratories.

Human biomonitoring: volatile organic compounds and organophosphates

With regard to fume events (with or without odour or visible components), it is still unclear which of the many contaminants provoke symptoms and health complaints. Repeated low-dose OP exposure and exposure to complex mixtures will not be addressed by toxicological assessment of individual compounds (57). However, in suspected fume events with symptomatic subjects, human biomonitoring strategies, combined with technical knowledge of the aircraft engineering may help to define the exposure time on suspected flights. Human biomonitoring strategies allow the different components of the contaminating sources to be toxicologically assessed in relation to the symptoms experienced and the toxic effects on human organ systems, including the heart, lungs and the nervous system.

The composition of the potential substances related to either normal or abnormal engine and aircraft operation, which contaminate the cabin air, has been described above. Specific sources of contaminants related to oil, aircraft fluids and fuel can be reviewed in Appendices 1A and 1B and in the published literature (30, 60, 61, 66, 70, 71, 80, 83, 87, 89, 90, 94, 95, 98, 146). The relevant descriptions and review of the literature form the basis for the selection of substances and groups of substances that are taken into account in human monitoring. Thus far, the main experience has been gained with various components, such as OPs (in flame retardants, lubricant additives) and volatile organic compounds (VOCs), which are described as ingredients or products of the pyrolysis of oil or hydraulic fluids.

Ongoing human biomonitoring is advantageous compared to air monitoring because it reflects the specific individual’s hazard load, in this case the VOC and OP levels, as reflected in body fluids (blood, urine) after exposure to a fume event and the ongoing natural history of the exposure (See Appendix 6 for further human biomonitoring information).

The method for human exposure biomonitoring blood and urine analysis has been published elsewhere (147, 148). Advantages and limitations of human biomonitoring are set out in appendix 7.

Blood analysis for volatile organic compounds

Blood should be taken as soon as possible after the fume event for human biomonitoring and at intervals as mentioned in Section 1B and Appendix 6:

- VOCs: Human biomonitoring can be undertaken for aldehydes, aliphatics, aromatics, ketones, alcohols and organics such as n-heptane, n-hexane, benzene, toluene, formaldehyde, acetaldehyde, n-pentane and n-octane (valeric acid/pentanoic acid, heptanoic acid, octanoic acid).

Urine analysis for organophosphates

There are several concerns related to measuring OPs or OP metabolites in urine. The first is that the short half-life of many OPs in the body ranges from hours to a few days. Secondly, research suggests that measuring a given metabolite does not necessarily mean that the individual was exposed to the parent OP because the metabolite can be taken up as a breakdown product directly from pyrolysis or may have been in the original formulation (149).

Analysis of modified proteins by mass spectrometry has important advantages over urine analysis. Firstly, the modified proteins have variable half-lives in the blood for example ranging from 12 days half-life in BChE to a 33-day half-life for red blood cell AChE. Secondly, this method avoids the necessity to determine pre-exposure baseline activity measurements in enzymatic assays as noted above.

When undertaking measurements of OP related urinary metabolites, the urine should be taken as soon as possible after the fume event for human biomonitoring and at intervals as mentioned in Section 1B and Appendix 6.

The following OPs are potential candidates for human biomonitoring: triaryl, trialkyl or triaryl-alkyl OPs. The analysis group for the OPs may include: tricresyl phosphates (TCP)[a]; trixylyl

phosphates (TXP)[a]; tributyl phosphate (TBP)[b]; triphenyl phosphate (TPP)[c], with other potential OPs being: dibutyl phenyl phosphate (DBPP)[b]; triisobutyl phosphate (TiBP)[b]; 2,6-di-tert-butyl-p-cresol (BHT)[b] and, isopropylated phenyl, phosphate (3:1) (TIPP/PIP (3:1))[c], as well as mixed esters – See Appendix 1B.

Note:

a. Utilised in selected oils.
b. Utilised in selected hydraulic fluids.
c. Utilised in selected oils and hydraulic fluids.

OP metabolites:

Urinary metabolites of T*o*CP in animal studies include *o*-Cresyl dihydrogen phosphate and Di-*o*-Cresyl hydrogen phosphate, salicylic acid, *o*-hydroxybenzyl alcohol and *o*-cresol (150, 151). However, T*o*CP present in engine lubricants is at very low levels and far lower than the more toxic ortho-TCP isomers, Mono-ortho cresyl phosphate and Di-ortho cresyl phosphate (M*o*CP and D*o*CP) (76, 77, 152).

Animal studies identified urinary metabolites of tri-para cresyl phosphate as *p*-hydroxybenzoic acid; di-*p*-cresyl phosphate (DCP); and *p*-cresyl *p*-carboxyphenyl phosphate (1coDCP) (153).

Three metabolites of tricresyl phosphate *oo*, *mm*, *pp* isomers – dicresyl phosphate (D*o*CP, D*m*CP, D*p*CP), as well as the dialkyl phosphate metabolites of tributyl phosphate (DBP), and triphenyl phosphate (DPP) were quantified in urine in 332 aircrew (48). TBP and TPP metabolites were identified in 100% of the aircrew. Median metabolite levels of tributyl phosphate (TBP), and triphenyl phosphate (TPP) (DBP 0.28 µg/l; DPP 1.1 µg/l) were found to be significantly higher than in unexposed persons from the general population. The maximum metabolite levels (DBP- 9.72 µg/l; DPP 302.2 µg/l) were significantly raised over the controls (48). However, the T*o*CP metabolite (D*o*CP) was not detected in any of the samples, while the T*m*CP and T*p*CP metabolites (D*m*CP, D*p*CP) were detected in only one sample at just above the detection limit.

Earlier PhD work by Schindler (not specific to aircrew or engine oils) reported that the D*m*CP and

D*p*CP metabolites (of the meta/para TCP isomers) could not be proven to be formed and excreted in urine in humans, with the 'probable' metabolites likely below the limits of detection (154). D*m*CP and D*p*CP could not be determined in any human urine samples (155). This therefore questions the adequacy of DCPs as human urinary metabolites of TCP after fume events.

With respect to human biomonitoring and physiological and pathological results and findings, it is important to recognise the short individual half-lives of urinary biomarkers of exposure of the substance or substance groups being tested, although the limitations in Appendix 7 should be considered. This again is why we recommend mass spectrometric analysis of adducted biomarker proteins.

Finally, in each fume event, irrespective of severity, it is important to consider the feasibility of obtaining a control sample from a non-exposed person. It is accepted that this may be difficult and, realistically, impractical. However, this subject is raised here for scientific completeness. It would be useful to compare the levels of adducted proteins to those found in the non-flying general population.

Note: OP levels may be inadequate to yield TCP metabolites. Once again, a negative result does not mean no exposure has taken place.

General

Various biomarkers of exposure have been recommended above. Cholinesterase activity levels may only show adverse effects following high levels of exposure during a fume event, but individual susceptibility varies widely. Although urinary metabolites for certain OPs (TBP & TPP) confirmed exposure, the metabolites for TCP are likely inadequate to show TCP exposure.

SECTION 3: LUNG/HEART

General background:

Respiratory and cardiac symptoms are generally well recognised and include breathlessness, cough, chest pain, tightness and discomfort/shortness of breath at rest or with exercise, wheezing, a sensation of chest and throat irritation, palpitations, irregular heart rate, tiredness, fatigue and general exhaustion at rest or with activity. Physical observations include cough, breathlessness, cyanosis, flushing, or an increase in blood pressure.

Respiratory system:

The respiratory tract is the most common portal of entry for aircraft air supply contaminants, although entry through the skin and alimentary tract is also recognised. Thus, it is not surprising that recurrent acute and chronic sinusitis and symptoms referable to the lower respiratory tract, such as cough and breathlessness, are common complaints among aircrew. Furthermore, not only is the upper and lower respiratory tract the main portal of entry in most cases, but it is also systemically more exposed than other organ systems because it receives the total cardiac output, thus, at least, theoretically increasing the possible toxicity (156).

Nasal and sinus pathology can be caused by inhaled pollutants. Research suggests that diesel exhaust particulates are potent in increasing and augmenting nasal airway resistance and nasal secretions (157). In an aircraft, flight deck, cabin crew and passengers may be similarly affected by contaminants including airport ground pollutants trapped within the aircraft cabin and those which may enter during flight, such as particulates, odours and contamination through the air conditioning system. The latter include de-icing and hydraulic fluids and pyrolysed engine oil entering in engine bleed air as seen in what is described as Aerotoxic Syndrome. Contaminated air may cause nasal and sinus disease but can also aggravate symptoms in people with pre-existing disease, particularly flight crew who have had long-term/repeated exposed to low cabin air humidity, which is recognised to harm nasal mucosa and promote sinus and upper airway conditions, such as chronic sinusitis.

The emerging evidence of the toxic effects of ultrafine particles on the respiratory tract is largely based on animal studies. Several studies suggest that carbon nanotubes cause systemic immune responses, pulmonary inflammation and fibrosis (158-160). The toxic effect of multi-walled carbon nanotubes is increased in conditions characterised by underlying inflammation, such as

asthma (161). The results of early animal studies indicate that ultrafine particle toxicity may lead to and account for currently observed human ill health in susceptible individuals. The presence of nanoparticles/ultrafine particles associated with the engine oils exposed to high temperatures has been reported (82, 92, 93). There is now a greater understanding of the toxic effects of fume events on the lung and other organ systems (57). A European Commission funded in vitro study exposing lung models to simulated pyrolysed oil and hydraulic fumes reported that *"exposure to engine oil and hydraulic fluid fumes can induce considerable lung toxicity"*(115).

Respiratory complaints among aircrew are common (9, 10, 14-19, 21-24, 26, 28-30, 34, 35, 40-42, 45, 47, 57, 125, 129). The Swiss Bureau of Air Safety reported that after a confirmed oil fume event a *"medical examination of the co-pilot the next day indicated a distinct adverse effect on the vocal cords and bronchial tubes as a result of a toxic exposure"* (162). Such complaints are consistent with lung injury secondary to VOCs and particulate inhalation and the effects are sometimes irreversible (57). Thus, respiratory symptoms are likely to be secondary to direct irritation or damage to airways and lung tissue. Substances that are not toxic individually may become highly toxic within a pyrolysed mixture (91). As an example of respiratory effects associated with the oils, the Eastman Turbo Oil 2380, safety data sheet states that *"Inhalation of thermal decomposition products may lead to adverse effects including pulmonary edema"* (163).

Furthermore, there is growing evidence that the response to low-level exposure to mixtures of toxic substances can differ from the response to acute, high-dose exposure to single toxins (164-166). The predominance of respiratory symptoms (second only to neurological symptoms) in these studies suggests there are appreciable levels of irritants in cabin air during fume events.

A variety of diagnoses have been applied to fume event-affected individuals including chronic bronchitis, sinusitis, asthma, reactive airways dysfunction syndrome and occasionally interstitial lung disease. Multiple chemical sensitivity, also called idiopathic environmental intolerance, may also be occasionally diagnosed (41). Long-term unbearable cough and breathing difficulties are some of the symptoms triggered by a variety of odours, smells and irritants, which are a common feature of people affected by a fume event.

Sarcoidosis has been associated with exposure to carbon nanoparticles, metal dust, inorganic particulates, fluid aerosols, some bacteria and exposure to inorganic triggers of inflammation

(167). An example of sarcoidosis is in a US Air Force veteran who had been exposed to petrochemicals, solvents, lubricants and jet fuel/aircraft engine/diesel exhaust (168).

In Australia, the New South Wales Court of Appeal found in favour of, and directed compensation to, a flight attendant who complained of a burning throat, sore eyes and headache and subsequently from a permanent cough after a fume event in 1992 when synthetic oil leaked from the auxiliary power unit into the aircraft cabin (169). The High Court of Australia subsequently upheld this judgement, which stated that inhaling heated Mobil Jet Oil II was harmful to the lungs (170).

Respiratory function testing

Routine/basic lung function testing (spirometry) in fume event-afflicted persons will often yield normal results. This is explained by the fact that routine spirometric tests are not sensitive enough and address only lung volumes and airway calibre. Lung injury caused by a fume event is often subtle, affecting the gas exchange part of the lung at the alveolar-capillary level. The single breath diffusing capacity for carbon monoxide (DLCO) and the single-breath determination of nitric oxide uptake (DLNO), if available, are sensitive tests for gas exchange abnormalities at the alveolar-capillary level and should be undertaken in all those presenting with respiratory symptoms (171). DLCO and DLNO tests require specialised equipment and expertise and referral to a respiratory laboratory is necessary. The measurement of expired nitric acid (FeNO), if available, is a simple method of assessing early pulmonary inflammation.

Lung function tests assess the diffusing capacity of the lung. However, exercise testing combined with measurements of arterial blood oxygen and carbon dioxide tensions and assessments of gas exchange generated by these measurements – such as the calculation of the alveolar-arterial oxygen gradient, venous admixture and true shunt – are more likely to detect subtle changes in gas exchange. However, these tests are semi-invasive, requiring an arterial puncture. A non-invasive alternative is measurement of oxygen saturation during exercise testing.

Oximetry is a simple method of measuring blood oxygenation and is non-invasive and a painless examination using a so-called 'thimble' with a small light source (LED) placed on a finger and from which the blood oxygen content can be determined.

Respiratory provocation testing by use of methacholine is sometimes indicated in cases of suspected fume event-induced asthma or reactive airways dysfunction syndrome.

Chest X-rays and high-resolution computed tomography (CT) chest scanning almost never get taken at the time of injury or within a short period, so it is difficult to know whether there are any acute radiological changes (43). In the presence of persisting respiratory symptoms, radiological examination of the lungs should be arranged. These investigations may also be required for reasons of differential diagnosis.

Lung function tests have been measured at the University for Medicine of Göttingen in Germany and other medical centres using standardised diagnostic methods to investigate the respiratory symptoms listed above. Preliminary results have been reported for about 350 patients experiencing symptoms following a fume event (42). With regard to routine spirometry, the relative distribution of $FEV_{1.0}$ (which is the forced expiratory volume a person can exhale in one second) and vital capacity is comparable to that of a normal healthy population. Subtle abnormalities with small airway obstruction and abnormalities in oxygen absorption (reduced alveolar–arterial gradient) were observed in the patients attending the University for Medicine of Göttingen who had experienced symptoms following exposure to a fume event, particularly the diffusing capacity (DLCO) and oxygenation during exercise testing (ergo spirometry) (42).

Measurements made during respiratory function testing are subject to slight variation related to inter- and intra-laboratory differences including device types, operator factors and to the time of day (172).

Heart:

The cardiac effects of OP exposure (173, 174) are less well known and are largely related to myocardial instability and arrhythmias. This subject has been reviewed by Roth et al., (173) and it is clear from their analysis that OP exposure is associated with cardiotoxicity. Many reports relate to OP ingestion associated with suicidal intent, but others document the outcomes of accidental ingestion with long-term effects of OP poisoning listed as a risk factor for cardiovascular diseases (175).

It is clear from all of these studies that OP exposure may cause electrocardiogram (ECG) changes (the detailed technical explanation behind the ECG changes is of prolongation of the QT interval and elevation of ST segments). Atrial and ventricular ectopic beats are commonly reported, but atrial fibrillation has been infrequently associated with inhalation lung injury (176). Ventricular fibrillation, late arrhythmias, bradycardia, tachycardia and sudden death associated with OPs have been documented (177-179).

While there are no systematically documented reviews into the alterations in heart rate and/or blood pressure after fume events, cardiac abnormalities reported by aircrew after fume events have been documented (9, 10, 14-16, 19, 21, 23, 24, 26-30, 35, 42, 45, 47). The higher rates of heart disease reported by flight attendants exposed to cabin air contaminants in this study suggest that they may be related (24) but the association was not confirmed in the authors' later study (25).

At the pathology level, patchy myocardial and pericardial damage has been reported in acute OP poisoning as a result of direct cardiac toxicity and may be a factor in serious cardiac complications. The authors warn that these findings may not result in ECG or echocardiographic changes, and, for this reason, cardiac monitoring is warranted (180).

An aircrew post-mortem study identified lymphocytic myocarditis, which was thought to be related to OPs (181). A UK coroner referring to other aircrew post-mortem cases noted that, in two cases of young fit aircrew, there was evidence of lymphocytic myocarditis and peripheral nerve damage (182). In another report, a left ventricular myocardial biopsy showed the histological features of a toxic myocarditis (183). Cardiac diagnostic procedures such as ECG, echocardiogram recordings, stress tests and myocardial perfusion scans are not routinely undertaken prior to or in-flight, so the pre-mortem cardiac status of affected persons has not been described.

In summary, OP exposure may lead to cardiotoxicity and has been shown to cause rhythm disturbances and myocardial damage. As the latter may be present in the absence of clinical signs and ECG changes, it is important to consider more detailed cardiac investigations in all cases, particularly if cardiotoxicity is suspected.

A number of the substances in the oils and aircraft fluids are associated with respiratory and cardio

effects under the Globally Harmonized System (GHS) and Classification and Labelling of Chemicals (CLP) systems (28, 72-75). As an example, an oil MSDS states in addition to a variety of neurological effects states*: "most important symptoms and effects both acute and delayed.... low blood pressure, bluish skin color..."* (184). There are various references to respiratory and cardio effects as highlighted by a REACH/CLP review (74). Two such examples include: (74)

- PAN: May cause damage to organs through prolonged or repeated exposure: cardiovascular, may cause allergy or asthma symptoms or breathing difficulties if inhaled.
- Oil base stock additives: May cause respiratory irritation.

Further details are presented in the irritant and sensitisation sections.

Recommended testing

Section 3A: Immediate post-flight /event

- Respiratory and heart rate.
- Auscultation of lung and heart.
- Blood pressure (if measurement and trained personnel available).
- Oxygen saturation SpO_2 (record inspired oxygen concentration, e.g. air, 2L/min by mask etc).
- Monitor oxygen saturation if <95%.
- Spirometry.
- ECG, if indicated e.g., presence of cardiac irregularity.
- Blood tests as clinically indicated.

Specialist tests within two weeks as required:

Respiratory function testing within two weeks:

- Detailed lung function tests (spirometry, DLCO and FeNO and/or DLNO, if available).
- Check oxygen saturation SpO_2.

Consider:

- Arterial blood gas analysis breathing room air at rest – undertake earlier if there is clinical need – see Notes, below.
- Expired nitric oxide (FeNO) if available.
- Exercise testing with oxygen saturation or blood gas analysis.
- Exhaled gas analysis (ergo spirometry, if available).
- Blood tests (troponin, if indicated e.g., presence of cardiac irregularity).
- ECG – if clinically indicated.

Notes

- Arterial blood gas analysis is a semi-invasive procedure that perhaps could be avoided in patients that do not complain about respiratory symptoms or who show an oxygen saturation value >96% at rest and/or during a six-minute walk test.
- Spirometry is a simple test measuring basic lung volumes that can be easily performed because it does not require sophisticated equipment. It should be performed promptly because symptoms of respiratory tract irritation may be transitory.
- Measurement of DLCO and/or DLNO are procedures that detect injuries of lung diffusion but are not available in all medical settings. However, these tests should be arranged in patients with respiratory symptoms, such as cough, shortness of breath, oxygen saturation <96% and in all those with abnormal spirometric values. The same approach should be applied for exercise testing or ergo spirometry.
- In case the aforementioned investigations are not available, or, in the presence of serious respiratory abnormalities, the patient should be referred immediately to a respiratory specialist or hospital.

Section 3B: Late / subsequent – if symptoms persist over weeks or months

If significant respiratory/cardiac symptoms are present or continue, consider referral to a respiratory specialist/pulmonologist and/or a cardiologist for an opinion and consideration of the following:

- Repeat routine lung function tests (spirometry, diffusing capacity).
- Static lung volumes.
- Percutaneous oxygen saturation or arterial blood gas analysis, as indicated.
- Appropriate radiology, for example, chest X-ray, high resolution lung scan (HRCT chest).
- Respiratory orientated exercise test or screen with six-minute walk test.
- Respiratory muscle strength testing.
- Bronchial provocation (methacholine, mannitol or other agent) testing.
- Blood tests as clinically indicated.
- Specific cardiac function tests as appropriate.
- Exercise testing with oxygen saturation or blood gas analysis.

SECTION 4: NEUROLOGICAL

General

Reported neurological symptoms may involve both the central and peripheral nervous systems. Central nervous system (CNS) effects recorded include: general incapacity, temporary paralysis, impaired or loss of consciousness; (severe) headache, pressure sensation in head; trouble speaking (dysarthria); balance problems, erratic movement, ataxia; memory deficits, cognitive impairment; vision problems, tunnel or double vision, dilated pupils, nystagmus and sleep problems. Peripheral nervous system (PNS) effects include motor, sensor and autonomic reactions such as: shaking, tremors, incoordination; muscle weakness; paraesthesia, numbness in limbs and other areas, peripheral neuropathy; sweating, loss of temperature control, pallor, flushing and altered taste.

Although standard neurological testing has often reported negative findings, neurological abnormalities in crew related to fume events have been regularly reported (9, 10, 13-19, 22-24, 26-30, 34, 35, 37, 38, 42, 45-47, 50, 125, 181). In the case of aircrew, chronic pre-exposure is assumed (28, 57, 59).

Target organ toxicity of the nervous system:

Tri-ortho-cresyl phosphate (T*o*CP) exposure has long been recognised in terms of the neurotoxic effects related to the acute high-dose end point and recognised as organophosphate-induced delayed neurotoxicity (OPIDN). Aircrew symptomology being observed does not match classical OPIDN, although there are clearly features in common. This is not surprising given the common mode of action of OPs. Industry risk assessment studies have focused almost entirely on OPIDN as the toxicological endpoint. They have inappropriately used this to justify the continuing safety of exposure to unfiltered bleed air suggesting the levels of T*o*CP are too low to cause OPIDN (185). The safety and concentrations of the more toxic mono- and di-o-CP are not considered.

Non-cholinergic mechanisms:

Repeated exposures to low levels of OPs are reported to cause a range of effects in the absence of overt signs of acute toxicity with the 'canonical' cholinesterase-based mechanism of OP toxicity being unable alone to explain the wide variety of adverse consequences of OP exposures (186,

187). The various mechanisms can cause harm at exposure levels below those required to cause lowering of acetylcholinesterase (59) with the OPs altering the function of a number of additional enzymes and proteins in addition to cholinesterase (186). "*OP interactions with proteins involved in fundamental neuronal processes such as axonal transport, neurotrophin support, and mitochondrial function (both oxidation-related processes as well as those that affect their morphology and movement in axons) may explain some of the more protracted effects of OPs*" (186).

Various non-cholinergic mechanisms have been described:

1) OPs alter the function of various enzymes and motor proteins involved in axonal transport. Axonal transport is responsible for the movement of lipids, mitochondria, synaptic vesicles, enzymes, receptor proteins and growth factors to and from a neuron's cell body through the cytoplasm of its axon (186). In an animal study repeated exposures (14-day exposure period) to OPs (chlorpyrifos) at doses that were not associated with acute signs of toxicity led to a significant reduction of both anterograde and retrograde axonal transport of vesicles in the sciatic nerves of rats. Chlorpyrifos is not associated with OP-induced delayed neurotoxicity, except at very high doses (187).
2) OPs can elicit oxidative stress to peripheral neuronal cells. Chronic low-level exposure has been shown to induce apoptotic neurodegeneration, impairing mitochondrial complex activities (187).
3) Chronic low-level exposure to OPs induces inflammatory responses by upregulating inflammatory cytokines (187, 188).

Non-OPIDN OP mechanisms might be related to the Aerotoxic Syndrome with it being suggested that neurons were impaired at very low T*o*CP concentrations, with functional endpoints being more sensitive than those focusing on neuronal structures (112). While the TCP focus has always been on T*o*CP, early oral animal studies showed that the meta and para isomers of TCP showed "*traces of demyelination in the spinal cord*" (135). Overall, the extensive studies outlined in the review by Terry (186, 187) fit with the clinical picture being observed in humans.

Biomarker studies identified that both the commercial formulation of TCP, DURAD 125 and the para isomer of TCP caused inhibition of various enzymes, rather than the T*o*CP isomer alone (113). It is also important to remember that the mono- and di-ortho-CP isomers are more toxic than ToCP and are present in higher concentrations than ToCP in anti-wear additives.

The central nervous system (CNS) is particularly vulnerable to toxic insult for a number of reasons. The nerve cells that are a component of the adult brain have to last for a lifetime. Many other organs in the body, for example the liver, can repair by cell proliferation. This does not apply to the nerve cells in the CNS. The brain has a very high metabolic rate and neurons have to maintain their microstructures over long distances. For example, the axon that carries outgoing signals from the neuron can be more than one metre long (Figure 1). To maintain such structures in a healthy state there is a mechanism called 'axonal transport', which will deliver a number of substances and structures – in both directions, to and from the neuron cell body. Transmitter substances help to deliver information across synapses (Figure 2) to the next neurons in the neuronal chain. Neurotrophins are also secreted across the synapse and are essential to maintain the target neurons in good health. Mitochondria are the 'powerhouses' in which pyruvate from glycolysis is metabolised to maintain the high metabolic rate essential for neuronal health, even in the most distant parts of the nerve cell.

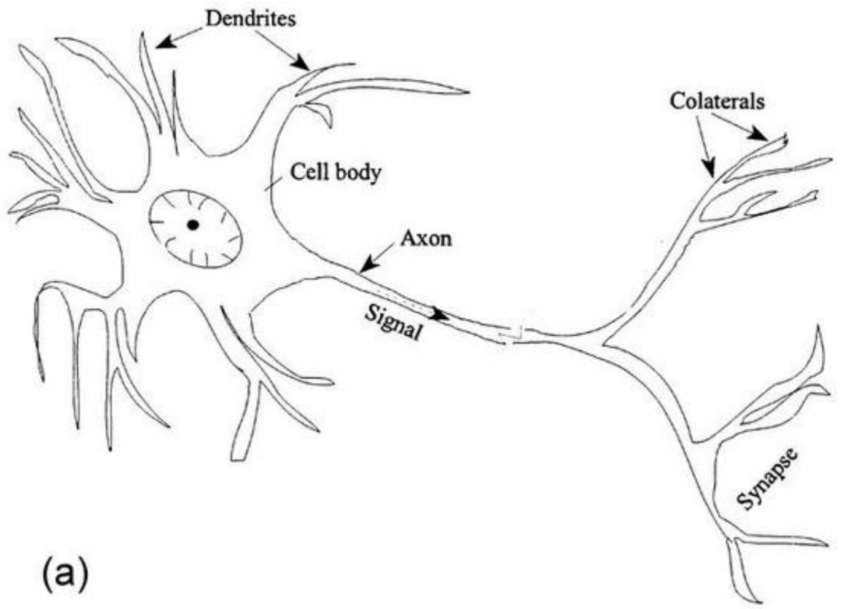

Figure 2: Schematic drawing of a nerve cell

Data source (189)

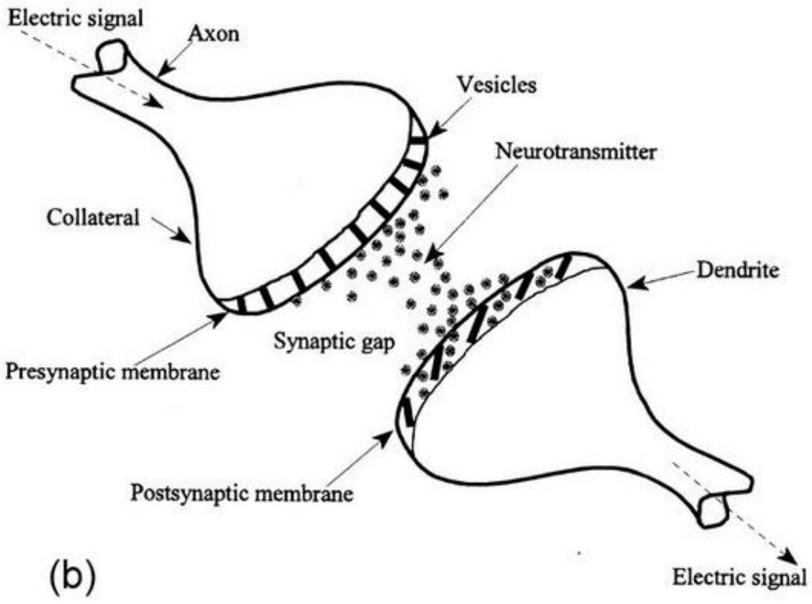

Figure 3: Schematic drawing of signal transfer between nerve cells

Data source (189)

The reaction of the CNS to toxic insult is variable. High-dose acute toxicity will cause acute toxic damage. However, repeated low-dose exposure to neurotoxic substances can cause subacute chronic toxicity over a long period of time (190). This is true of OPs. OP nerve agents (for example, soman, sarin, VX) can cause acute death by inhibiting the enzyme anticholinesterase. However, of much more relevance to the aetiology of Aerotoxic Syndrome, chronic low-dose exposure to OPs at levels well below any cholinergic symptoms can cause neurotoxic effects. Terry has reviewed this topic and shown that axonal transport can be affected by repeated low-dose OP exposure (186, 187). This would interfere with the delivery of transmitter substances, neurotrophins and mitochondria, to target neurons and could be the basis for the development of a diffuse subacute encephalopathy (57).

The existing literature on low-dose repeated exposure to OP compounds was analysed with respect to medical problems being reported among aircrew, concentrating on non-cholinesterase mechanisms at levels of exposure that produce no overt signs of acute toxicity (28). These include covalent binding of OPs to tyrosine and lysine residues, which suggests that numerous proteins can be irreversibly modified by OPs. In addition, the mechanisms of oxidative stress, and neuro-inflammation combined with the known OP targets of motor proteins, neuronal cytoskeleton,

axonal transport, neurotrophins, serine active site enzymes and mitochondria means that there is a potential mechanism for chronic irreversible neuronal damage.

The nature of exposure to fugitive emissions from gas turbine engine bleed air to the concept of 'dose' when dealing with irreversible molecular processes is relevant, particularly with respect to the extended periods of exposure experienced by aircrew over a working lifetime. Additionally, the toxicology of complex mixtures is important, and the potential effects of the continual presence of ultrafine particles in engine bleed air was also considered (57).

Overview of organophosphate toxicity:

The symptoms associated with the toxicity of OPs involves three main categories, with a newer fourth area related to repeat low-dose exposures as described above. The classical description in OP poisoning is that of nerve gas poisoning caused by the inactivation of the enzyme acetylcholinesterase. The resulting effects are classified as 'muscarinic' (airway over secretion, bronchial constriction, slow heart rate, gut over motility, sweating, pupillary constriction), 'nicotinic' (muscle fasciculation and weakness, fast heart rate, raised blood pressure, pupillary dilatation) and 'central' (headache, tunnel vision, staggering gait, disorientation, coma). It should be noted that these are high-dose health effects. Chronic repeated low-dose exposure to OPs, the norm with air crew, can lead to adverse neurological effects at levels below those required to cause cholinergic effects. For completeness, the differing type of effects are listed below.

Organophosphate-induced toxicity due to acetylcholinesterase inhibition: Cholinergic toxicity

> The primary action of OPs is the irreversible inhibition of acetylcholinesterase (AChE), resulting in accumulation of acetylcholine and overstimulation of the nicotinic and muscarinic AChE receptors with cholinergic effects. OPs inactivate cholinesterases by attaching an alkyl phosphate group to the hydroxyl group of a serine residue at the enzyme's active site. Recovery from such inhibition generally takes 10–14 days (191). Cholinergic symptoms depend on the OP compound, size of the dose, frequency, duration and the route of exposure, combined exposure to other chemicals and individual sensitivity and susceptibility (20, 192, 193). Initial symptoms of mild toxicity include fatigue, dizziness and sweating, sometimes accompanied by headache, inability to

concentrate, cognitive dysfunction, weakness, anxiety, tongue and eyelid tremors, miosis and tightness of the chest. Moderate poisoning may result in salivation, lacrimation, abdominal cramps, nausea, vomiting, slow pulse, bradycardia, fall in blood pressure and muscular tremors (20). For the sake of completeness, we list severe OP exposure symptoms, however this is not what is generally being observed in practice. Severe exposure symptoms include: pinpoint and non-reactive pupils, muscular twitching, wheezing, increase in bronchial secretion, respiratory difficulty, cough, pulmonary oedema, cyanosis, diarrhoea, loss of sphincter and urinary bladder control, tachycardia, elevated blood pressure, convulsions, coma, heart block, and possibly death (20).

Single acute toxic exposures to OP esters generally range from 5 to 60 minutes after exposure but may not occur in some cases until 24 hours after exposure. Repeated small OP chronic exposures have cumulative effects with early symptoms being flu-like. As exposure continues, clinical manifestations appear until the full pattern develops (20).

The clinical picture that we are observing is not like nerve gas poisoning. The symptoms being observed are more diffuse as described in Michaelis et al. (28).

Organophosphorus-induced delayed neurotoxicity

Organophosphorus ester-induced delayed neurotoxicity (OPIDN) is a central and peripheral axonopathy with the early stage characterised by peripheral effects that recover as peripheral nerves regenerate. The later stage is central, which is more permanent. OPIDN may be caused by single or repeated exposure and is accompanied by a Wallerian type (or dying back) axonal degeneration and secondary demyelination in the most distal portion of the longest tracts in both the central and peripheral nervous system (192). The clinical picture is manifested by mild sensory disturbances, ataxia, muscle fatigue and twitching, and improvement may require months or years. A full description of the varying phases is available (20, 193). The earliest recorded cases of T*o*CP-induced OPIDN have been documented in 1899, attributed to the use of creosote oil for treatment of tuberculosis (194). It is important to note that not all people will exhibit the same effects, however the clinical picture being observed does not fit this pattern.

Organophosphorus-induced chronic neurotoxicity

Excessive cholinergic activity produces delayed neurodegeneration in various brain areas (cortex, cerebellum, (hypo)thalamus, amygdala and spinal cord, that could explain persistent neuropsychiatric, neurologic and behaviour problems (192). Organophosphorus-induced chronic neurotoxicity (OPICN) is associated with exposure to large acutely toxic or small subclinical doses of OP compounds. Clinically, OPICN is manifested by headaches, drowsiness, dizziness, anxiety, apathy, mental confusion, restlessness, labile emotions, anorexia, insomnia, lethargy, fatigue, inability to concentrate, memory deficits, depression, irritability, confusion, generalised weakness, tremors, respiratory, circulatory and skin problems, with not all people exhibiting all these symptoms (20). Reports on OPICN occurring in individuals, following long-term, subclinical exposures without previous acute poisoning, have been inconsistent, partially due to the difficulty in defining exposure levels (192). Although largely characterised by chronic neurobehavioral alterations, OPICN involves other molecular, neurochemical, neurophysiological, neuropathological, neuropsychological and neurological changes (192). Although the symptoms of OPICN are a consequence of damage to both the PNS and CNS, they are primarily related to the CNS injury and resultant neurological and neurobehavioural abnormalities (192). Symptoms may persist for years after exposure and are distinct from cholinergic and OPIDN effects (192). These sorts of symptoms have been noted by Terry (186) in non-cholinergic mechanisms of OP toxicity.

Low-dose exposure

Repeated low-dose exposure and a number of other mechanisms are described in research published in 2012 by which OPs can cause harm at exposure levels below those required to cause lowering of AChE, such as oxidative stress, neuroinflammation, and impaired axonal transport (186). Further details have been outlined above.

Summary

We have included a full description of various aspects of the neurotoxicology of OPs, for the sake of completeness. Many clinicians will be familiar with the high-dose acute exposure scenario affecting cholinesterase enzymes. Fewer will have encountered the low-dose repeated exposure

scenario, which has not yet filtered down to some mainstream toxicology/pharmacology texts. A question therefore remains on how to put this information into perspective for clinicians facing the outcome of an aircraft fume event.

The typical clinical picture in aircrew reporting symptoms consistent with Aerotoxic Syndrome and who have or have not suffered a recognised fume event, has been described by Michaelis et al. (28). This does not amount to a classical appearance of OPIDN. Clearly there are features in common, to be expected because of a common mode of action between OPs, however it seems reasonable to state that because presenting symptoms are more consistent with a diffuse encephalopathy than a florid case of OPIDN.

The toxicity of the mixture to which aircraft passengers are exposed is being underestimated by a large margin (185). OPs have been shown to be able to synergise in mixtures. Chronic low dose pre-exposure of neuroblasts to OPs made them more susceptible to toxicological damage when challenged with a higher OP dose (195, 196).

After a fume event, aircrew have been shown to seek medical assistance with much higher frequency than passengers, as shown in Figure 4 (59, 197), which is consistent with an increase in aircrew susceptibility to OP damage as a consequence of chronic repeated low-dose pre-exposure. In high dose fume events, for example with a visible haze in the cabin, more 'cholinergic' symptoms may be likely to be encountered on occasion, but this is certainly not inevitable.

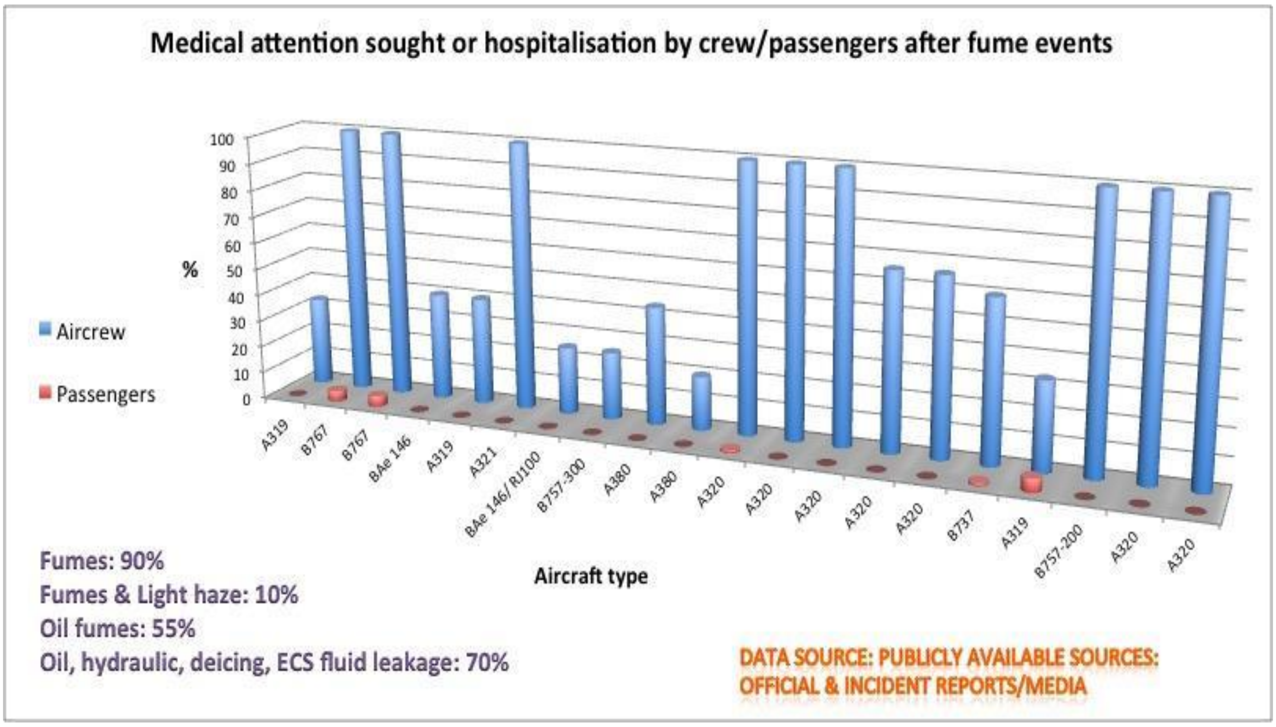

Figure 4: Medical attention sought or hospitalization by crew/passengers after fume events (2000-2018)

Data source: (197)

Central nervous system:

The neurological pattern of symptoms reported by Michaelis et al. (28) and elsewhere when considered together "*constitute a group of non-localizing functional deficits which are consistent with a diffuse toxic encephalopathy*" (57). The pattern is reported to be in many ways "*directly comparable with the symptoms suffered by farmers from 'dipper's flu'*, (198). *The common aetiological factor being exposure to organo-phosphorous compounds*" (57). A toxico-pathological explanation of the influence of continual low dose exposure to a complex mixture of OPs has been suggested by Howard et al., (59).

In a study of aircrew involved in 15 individual incidents or series of fume events, a range of reported neurological findings and diagnoses included: neurotoxic injury, toxic encephalopathy, neuropathy on vocal chords/limbs, cognitive dysfunction, dementia, attention deficit hyperactivity disorder (ADHD), seizure disorder, neurological chemical injury, CNS injury, Wallerian degeneration, frontal lobe damage, optic nerve damage and migraines, to record just a few (28). The various diagnoses were associated with a wide variety of acute symptoms ranging

from impairment to incapacitation at the time of and subsequent to the event as well as a variety of short term medical findings and diagnoses (28).

In most cases these symptoms appear to onset during the flying career and show a temporal relationship with time spent on board aircraft as they onset or worsen when flying and reduce or resolve during days off. Most crew reported that their symptoms occurred after exposure to fumes in the cabin.

Functional brain scans have been a favoured approach over structural magnetic resonance imaging (MRI) scans in a study of 26 cabin crew after one or multiple fume events (38). These included single photon emission computed tomography (SPECT) and positron emission tomography (PET) brain scans after neurotoxic exposures. Significant abnormalities were identified, supported with neurological and neuropsychological evaluations with toxic encephalopathy diagnosed. "*Most striking was the frequent occurrence of hypofrontality (decreased frontal and increased posterior brain function), and increased function in some limbic areas*" (38).

State-of-the-art MRI scans, in one study, identified small clusters in the brain in which white matter microstructure was affected in aircrew reporting cognitive impairment and depressive symptoms (37). Higher cerebral perfusion values in the left occipital cortex and reduced brain activation on a functional MRI executive function task was observed and the extent of cognitive impairment was strongly associated with white matter integrity (37).

Advanced MR-imaging, PET or SPECT scans may not be available or may be normal. In that case, objective evidence of central nervous system injury can be found by detecting serum brain specific autoantibodies (see autoantibodies sections 1B and 2). For example, 34 air crew with CNS related complaints were found to have higher autoantibody levels than matched controls (45).

An autopsy on a crewmember provided histopathology results that showed axonal degeneration and demyelination of the brain and spinal tissues. Both the post-mortem and pathological examination of the central nervous system confirmed the autoantibody biomarker results reported elsewhere (45, 181, 199).

In addition to the direct and delayed OP-induced neurotoxicity effects, concern exists about the

potential long-term risks of neurodegenerative disease in pilots and flight attendants exposed to cumulative low-dose OPs. While still the subject of debate (200), numerous studies have reported a correlation between exposure to OPs and development of neurological system diseases including Parkinson's, amyotrophic lateral sclerosis/motor neurone disease and Alzheimer's (187, 192, 201-203). Many of these diseases have a genetic and environmental component. The environmental exposure to OPs seems to accelerate the development of these brain diseases (192, 201), which would be in keeping with a diffuse encephalopathy.

Several cohort studies report increased disease rates for motor neurone disease and a twice as high mortality rate of amyotrophic lateral sclerosis (ALS, the most common form of progressive motor neuron disease), when compared to the general population (21, 39, 204, 205).

Neurobehavioural and neuropsychological effects

Reported neurobehavioural and neuropsychological health effects associated with fume events include discomfort, intoxication, disorientation, confusion, altered behaviour, personality changes, unreality, anxiety, depression, dizziness, lightheadedness, lethargy and drowsiness. Neurobehavioural cognitive effects reported include cognitive problems, difficulties with problem solving, concentration, memory and writing, giggling and euphoria. These symptoms have been widely reported (9, 10, 13-18, 21-24, 26-30, 32-38, 42, 45-47, 50, 125).

Slowed information processing speeds, slowed reaction times and executive dysfunctions were identified in pilots and flight attendants (32, 33, 35-37). In a survey of international air crew, 45% of the respondents reported that they suffered from confusion and difficulty in thinking, 55% said they had difficulty concentrating, and 49% said they had memory loss (26).

A study reviewing 15 incidents related to contaminated air exposures identified that acute cognitive adverse effects were reported by one or more or all crew in 93% of the incidents, with 60% showing longer-term effects in one or more or all crew (28). Neurobehavioural effects were recorded as the highest category of adverse effects at a rate of 64% in pilots identified with long-term chronic ill health (21, 28). It is reported that the *"pattern of test results reflects studies that have been carried out in other occupational settings where workers were exposed to*

organophosphates and solvents" (32) and that study results mirror *"that seen in other OP-exposed cohorts"* (36). Additionally, a *"significantly higher number of* cognitive tests scored in the impaired range in aircrew compared to controls, and the extent of cognitive impairment was strongly associated with brain white matter integrity" (37). As previously reported *"chronic (or repeated) exposures to OPs at levels that are not associated with acute toxicity can result in a variety of neurobehavioral symptoms, particularly cognitive deficits"* (186).

Peripheral nervous system:

In surveys of health symptoms in pilots and cabin crew sensory complaints, such as paraesthesia, tingling and numbness are reported in 20-77% of cases (16-19, 28, 45). This may be caused by OP-induced delayed neurotoxicity (see NTE, section 1B), however in this relatively rare disease in most cases motor symptoms, muscle pain, distal weakness (foot drop, bilateral wrist weakness) prevail. The clinical picture of T*o*CP-induced delayed neurotoxicity is well known from several outbreaks reported throughout the world (ginger jake paralysis): acute paralysis of distal limbs, subsequent development of pyramidal tract signs and only minimal sensory disturbances. In addition, nerve conduction studies show pure motor symmetrical distal axonal neuropathy. This is not consistent with the clinical symptoms reported by cabin crew. It appears that there is a distinct toxic predominantly sensory polyneuropathy in patients with probable Aerotoxic Syndrome.

Winder and Balouet described sensory symptoms (tingling, numbness) in 5 of 7 pilots, pursers and flight attendants who had been exposed to cabin contamination (15). In the past 20 years, peripheral nerve impairment was studied in larger studies of patients with probable Aerotoxic Syndrome. Various complaints of patients, attending the occupational clinic of the University for Medicine of Göttingen in the period of 2014–2017, as presented by Heutelbeck in a progress report (42), were correlated with fume events, such as restless legs, muscular jerking and tingling sensations. Paraesthesia/tingling feelings of the hands were reported in almost 30% of 34 flight crew members in one study (45), and in 13 of 38 cases (34%) in another study (19).

Gross neurological status (balance, muscle weakness, numbness, reflexes) can possibly identify balance problems, muscle fasciculations and reduced sensation (42). One frequently used test is vibration sensitivity, which evaluates peripheral somatosensory function. In chronic low-dose OP-

exposure a decreased sensitivity is described in farmers and sheep dippers (206). In most patients with probable Aerotoxic Syndrome, nerve conduction was found to be unremarkable despite a credible and similar pattern of complaints between patients (42). However, a few studies report mild sensory polyneuropathy (16, 28, 34).

Symptoms of small fiber neuropathy, a subtype of polyneuropathy of thin myelinated and unmyelinated nerve fibers, are painful burning paraesthesia and hypersensitivity to touch and temperature changes (207). Diagnostic criteria are based on: 1) Clinical signs (pinprick and thermal sensory loss, hyperalgesia); 2) Quantitative sensory testing (abnormal warm or cold thresholds); 3) Reduction of intraepidermal nerve fibers by skin biopsy; and 4) Exclusion criteria were any clinical sign of large fiber impairment (light touch and vibratory sensation, absent deep tendon reflexes) or any abnormality at nerve conduction studies (208). There is a guideline on the use of skin biopsy in the diagnosis of this neuropathy, reported by a joint task force of the European Federation of Neurological Societies and the Peripheral Nerve Society (209). In the context of exposure to solvents and T*o*CP a reduced density of epidermal nerve fibers is described (207). Research published in 2019 describes a large study of patients with small fiber neuropathy that demonstrated that quantification of intraepidermal nerve fiber density (skin biopsy) remains the most reliable tool to confirm diagnosis for small fiber neuropathy (210).

The above symptoms of small fiber neuropathy are reported less frequently by pilots and cabin crew. However, there is one study on the prevalence of small fiber neuropathy in patients with probable Aerotoxic Syndrome (42). In nearly all patients in this study group, neuropathological investigation by skin biopsy showed that the intra-epidermal nerve fiber density was significantly decreased and, as such, clearly under the mean value of the healthy norm population. So, this sensory, typically painful, disease may well be responsible for both sensory and autonomic complaints in pilots and cabin crew.

<u>Summary:</u>

The mechanism of toxicity of OPs is primarily attributed to inhibitory actions on various forms of cholinesterase, leading to excessive peripheral (and central) cholinergic activity. There is evidence that this cholinesterase-based mechanism cannot alone account for the wide variety of adverse consequences of organophosphate exposure – especially in people associated with repeated exposures to levels – that produce no overt signs of acute toxicity (186, 187).

Many of the symptoms (especially the neurobehavioral symptoms) that have been associated with "Aerotoxic Syndrome" have (for decades) been documented in agricultural workers and pesticide sprayers as well as veterans of the United States, United Kingdom, Canada, France, and other European countries who served in the 1990–1991 Persian Gulf War. In both scenarios, organophosphate exposure (i.e. as pesticides or nerve agents) has been discussed as a plausible explanation for the chronic neurologically-based symptoms (187).

The various substances in the oils, hydraulic and de-icing fluids are listed under the Globally Harmonized System (GHS) and Classification Labelling and packaging of Chemical (CLP) hazard classification warning systems with regards to the neurological system (28, 57, 69, 72-75). Refer to various MSDSs and other material for recognised neurological hazards associated with exposure to the fluids presented here. An oil MSDS states:

- Mobil Jet oil II MSDS (2019): *"Most important symptoms and effects both acute and delayed - Headache, dizziness, drowsiness, nausea and other CNS effects. Shallow respiration, low blood pressure, bluish skin color, convulsions, coma and jaundice"* (184).

Recommended testing:

Section 4A: Immediate post-flight/event

- Full general medical assessment.
- Detailed neurological assessment and examination.
- Objective assessment of vestibular function.
- MRI brain scan.
- Consider referral to a neurologist for severe neurological symptoms and signs.

Section 4B: Late/subsequent

If symptoms persist over weeks or months:

- Full general medical assessment.
- Detailed neurological assessment and examination.
- Objective assessment of vestibular function.
- MRI – Refer to methodology in (37).
- PET/SPECT – Refer to methodology in (38).

- EMG/ENG: polyneuropathy.
- Skin biopsy/IENF (intraepidermal nerve fibers) – Small fiber neuropathy, refer (209). There is an international guideline on how to perform this diagnostic (209).

Section 4C: Neurocognitive

Neurocognitive tests that are deemed applicable include the following areas:

- Processing speed, written and oral.
- Attention and concentration.
- Reaction time to stimuli.
- Sequential reaction time.
- Complex problem solving.
- Short and long term visual and verbal memory.
- Cognitive flexibility/capacity to change direction.

Neurocognitive testing:

- Coding test from WAIS.
- Symbol Digit Modalities Test (written and oral versions), see Section 1B.
- CALCAP – Simple and choice reaction time tests.

Note: All tests should be able to be administered by medical personnel.

If neurobehavioural/neuropsychological symptoms persist over weeks or months

Formal neurocognitive testing:

- Tests for processing speed such as the Coding Test or Wechsler Adult Intelligence Scale (WAIS), Symbol-Digit Modalities Test (written and oral), Symbol Search (WAIS) and Trail Making Test A.
- Tests of new learning, such as the Austin Maze and the Rey Auditory Verbal Learning Test (RAVLT).
- Memory tests, such as those in the Wechsler Memory Scale, including visual and verbal memory.

- Problem solving tests, such as the Category Test. The Wisconsin Card Sorting Test and the Stroop Test.
- Fine motor tests, such as the Reitan Finger Tapping Test of manual speed, the Grooved Pegboard Test of manual dexterity and the Dynamometer Grip Strength Test.
- In case of sleep disturbances consider full polysomnography.
- Boston Naming Test of language skills.

Similar and alternative tests have been utilised in other studies with aircrew after a fume event (32, 33, 35, 36). Tests available may vary in different countries; however, most of the tests listed are universal and come primarily from the US.

As an example, tests utilised in Germany may include:

- FAKT-II
- RSAT
- KVT-C
- IGD
- CompACT-VI
- Regensburger Wortflüssigkeits-Test

SECTION 5: IRRITANTS

The various substances in the oils, hydraulic and de-icing fluids are listed under the Globally Harmonized System of Classification and Labelling of Chemicals (GHS) hazard classification warning system as irritants to the respiratory tract, eyes and skin (28, 69, 72-75). The irritant nature of the fluids and pyrolysis substances is widely recognised (70). Eye, nose, throat, skin and respiratory irritation have been frequently reported in association with fume events (9, 10, 14-19, 21-30, 34, 35, 41, 45, 47, 57, 71, 125). The enhancement of dermal irritant effects associated with used engine oil exposures raised concerns about increasing dermal impacts with prolonged and/or repeated exposures (211).

The UK aviation regulator, when examining pyrolysed oil, reported that, *"the symptoms of irritation could be induced by short-chain organic acids formed during pyrolysis of aircraft lubricants"* (61).

In addition to the various irritant hazard warnings under the GHS system and international chemical hazard databases, the oil material safety data sheets (MSDS) and the OP additive tricresyl phosphate (TCP) MSDS often report that they are associated with eye, nose, throat, respiratory and skin irritation. Several examples include:

- Mobil Jet Oil II (2017): *"Symptoms from acute exposure to these decomposition products in confined spaces may include headache, nausea, eye, nose, and throat irritation"* (133).
- Boeing Lubricating oil (2007): *"Signs and symptoms of exposure: Exposure may cause irritation, characterized by tears, redness and burning sensation (eyes), redness, swelling or cracking of the skin, or burning sensation in the nose, throat and lungs (inhalation)"* (212).
- Mobil (1983): *"if cabin air becomes contaminated with any lubricant and/ or its decomposition products in sufficient quantities, some degree of discomfort due to eye, nose and throat irritation could be experienced"* (213).
- TCP (1991): *"It is an irritant of the skin and eyes. It is also an irritant of the mucous membranes and respiratory tract"* (214).
- Durad 125/TCP (2006): *"May cause mucous membrane and upper respiratory tract irritation. Mist generated by heat will irritate skin, eyes, nose, throat and respiratory system"* (215).

- Mobil Jet Oil II label (1999): *"prolonged or repeated breathing of oil mist, or prolonged or repeated skin contact can cause nervous system effects... Avoid prolonged or repeated overexposure to skin or lungs"* (216).
- Eastman Turbo Oil 2197 label (2018): *"Do not breathe mist or vapour from heated material. Do not get in eyes and avoid contact with skin and clothing*" (217).
- Mobil Jet Oil II (2017): *"Negligible irritation to skin at ambient temperatures"* (133).

Hydraulic and de-icing fluids are associated with skin, respiratory and eye irritation (28, 73, 74).

Some examples of wording on MSDS's for hydraulic and deicing fluids include:

- ExxonMobil HyJet IV-A Plus (2021): "*When heated, the vapors/fumes given off may cause respiratory tract irritation"* (218).
- Eastman Skydrol PE-5 (2019): *"Causes skin irritation... Symptoms: ...Irritation, pain, rash, redness..."* (219).
- Dow UCAR PG Aircraft Deicing Fluid (2009): "*Vapor from heated material or mist may cause respiratory irritation and other effects"* (220).

Various studies have reported vocal and nasal polyps and sinus irritation among workers (10, 14-16, 21-24, 26-28, 30, 35, 57). For further description of the toxic ingredients and health hazards associated with exposure to these fluids, refer to specific safety data sheets.

Management: Immediate post-flight / Late/subsequent:

- Avoid ongoing exposure to irritants.
- Manage symptoms as appropriate to the organ system involved.

SECTION 6: SENSITISATION

A number of the substances in the oils and aircraft fluids are associated with sensitisation under the Globally Harmonized System (GHS) and Classification and Labelling of Chemicals (CLP) systems (28, 70-75). For example, tricresyl phosphate (TCP), trixylyl phosphate (TXP), isopropylated phenyl phosphates (TIPP/PIP (3:1)) and phenyl-α-naphthylamine (PAN) are recognised as skin sensitisers and PAN is associated with allergy or asthma symptoms and breathing difficulties (73, 74). Some of the substances in the hydraulic fluids are associated with both skin and respiratory sensitisation. For example, warnings about exposure to PAN, a common antioxidant in the oils, include *"Repeated or prolonged contact may cause skin sensitization"* (132) and PAN *"N-phenyl-1-naphthylenamine... may produce an allergic reaction"* (163).

An example of MSDS wording highlighting sensitisation include:

- Eastman Skydrol PE-5 (2019): *"May cause an allergic skin reaction"* (219).

See further hazard warnings in Section 7 Skin.

Both respiratory and skin sensitisation has been widely recorded in aircrew studies (15-19, 21, 23, 24, 26-28, 30, 41). For further acknowledgement of hazards associated with fluids, refer to specific safety data sheets etc.

<u>Management: Immediate post-flight / Late/subsequent:</u>

- Avoid ongoing exposure to irritants.
- Manage symptoms as appropriate to the organ system involved.
- Consider referral to organ system specialist.

SECTION 7: SKIN

Skin rashes have been reported among airline cabin crew and pilots (15-17, 19, 21, 22, 24, 26-30, 34, 41, 45, 70). These are usually transient but may recur. Selected MSDSs list adverse effects on the skin as a symptom of exposure to the oils. Examples include:

- *"oil acne/folliculitis signs and symptoms may include formation of black pustules and spots on the skin of exposed areas"* (221).
- *"Prolonged or repeated skin contact without proper cleaning can clog the pores of the skin resulting in disorders such as oil acne/folliculitis"* (221).
- *"Irritant and allergic dermatitis have been reported"* (222).
- *"Prolonged skin contact may defat the skin and produce dermatitis"* (163, 223).
- *"Excessive skin contact may cause skin irritation, but the practice of good personal hygiene should prevent the occurrence of any problems of dermatitis"* (224).

For further acknowledgement of hazards associated with fluids, refer to specific safety data sheets etc.

Management: Immediate post-flight / Late/subsequent:

- Avoid ongoing exposure to irritants.
- Consider standard dermatological treatment.
- Manage symptoms as appropriate to the organ system involved.
- Consider referral to a dermatologist if symptoms are recurrent.

SECTION 8: GASTROINTESTINAL – IMMEDIATE POST-FLIGHT / LATE/SUBSEQUENT

Nausea has been very commonly reported by aircrew during and shortly after fume events, with some reporting ongoing gastrointestinal effects (9, 10, 13-19, 21-23, 26-30, 34, 35, 41, 42, 45-47, 125). Other gastrointestinal symptoms reported include vomiting, diarrhoea, cramps, bloating, digestive problems, and pain. The MSDSs for oil frequently report nausea as an acute effect, as is also the case with hazard substances databases (214).

There are various references to effects on the liver in the GHS and CLP chemical classification systems in relation to prolonged or repeat exposure to various substances in the oil, hydraulic and deicing fluids (72-74). Jaundice has been listed as a key finding in an oil MSDS (184), while effects on the liver after prolonged or repeat exposure are identified in a commonly used hydraulic fluid (218).

Occasional abnormalities of liver function have been reported. Transient gastrointestinal symptoms are listed as an outcome of high-dose acute oral exposure to TCP (76). Changes in this regard are reported again and again by flight crew, but to date, there are no systematic insights into appropriate diagnostic methods.

Note: Gastro-intestinal symptoms may also develop in connection with small fiber neuropathy.

Management: Immediate post-flight / Late/subsequent

- Investigations as clinically indicated.
- Consider referral to a gastroenterologist.

SECTION 9. OTHER

Other clinical symptoms and medical conditions reported by those exposed to aircraft contaminated air fume events include:

Fatigue/chronic fatigue:

Fatigue has routinely been reported as both a short- and longer-term effect related to FE exposures (15-19, 21-24, 26-30, 34, 35, 41, 45-47, 125).

Chemical sensitivity:

Sensitivity to chemicals has been regularly reported by aircrew reporting fume event exposures (15-18, 21-23, 25-28, 30, 34, 41, 45). The relationship between chemical exposures and chemical sensitivity has been reported (70, 71, 225-228).

Where a crewmember reports an intolerance to certain chemicals, a 50 question 'Quick Environmental Exposure and Sensitivity Inventory' (QEESI) questionnaire could be considered as part of a physician's clinical evaluation (229). There is also a three-question 'Brief Environmental Exposure Sensitivity Inventory' (BREESI), which may be a useful screening tool (229, 230). The BREESI and QEESI surveys assess symptoms of chemical intolerance at a point in time and can be downloaded and used free of charge at www.tiltresearch.org. The physician may also administer an additional QEESI to document whether and to what degree the crewmember recalls symptoms prior to a documented fume event or prospectively at intervals to follow symptoms over time.

Reproductive symptoms:

There are limited data supporting aircrew reporting fume event exposures and infertility and a range of other reproductive signs and symptoms (16, 25, 26). Anecdotal reports are often cited. Of note, TCP is listed on the European Union's Classification, Labelling and Packaging (CLP) Regulation ((EC) No. 1272/2008) as a chemical notified in their database as "*Suspected of damage to fertility or unborn child*", while TXP is listed as toxic for reproduction REP 1B on the Globally Harmonized System (GHS) and Classification Labelling and packaging of Chemicals (CLP) as "*may damage fertility*" and on the substance of very high concern candidate list on the European Chemicals Agency database (28, 72-75). In a similar manner

TIPP/PIP (3:1) used in some hydraulic fluids meets the classification of being suspected of causing *"damage to fertility or unborn child"* (73, 74). Fertility problems are included on some of the MSDSs for TCP and some of the oil cans. Limited examples are provided below outlining recognition of reproductive effects. Further information can be found under a REACH/CLP review of the relevant substances (74).

Mobil Jet Oil II (2020): "Reproductive toxicant (fertility): Category 2 Suspected of damaging fertility" (231)
ExxonMobil HYJet IV-A Plus (2021): Reproductive toxicant (developmental): Category 2. Reproductive toxicant (fertility): Category 2 (218)
Eastman Turbo Oil 2197 (2020): "Suspected of damaging fertility or the unborn child" (223).

Malignancy / mutagenicity:

There are emerging reports of increased levels of selected cancers in aircrew. Occupational risk factors include exposure to ionizing radiation, disruption of circadian rhythms, and exposure to chemical toxins. McNeely et al. (24) reported a 34% increased rate of female reproductive cancers (breast, ovary, uterus) in flight attendants in comparison with the general population. The finding was confirmed in a second study published in 2018 that also reported a higher incidence of cancers in flight attendants than the general population (breast, ovary, uterus, cervix, lung, oral, oesophagus, prostate, testis, colon, bladder, melanoma, non-melanoma skin, leukaemia, thyroid, brain, lymphoma, liver, kidney, stomach and pancreas) (25).

The incidence of female reproductive cancers (breast, uterine, cervical and ovarian) were 66% higher than the alternate study population (25). This was suggested to be consistent with most of the epidemiologic literature on this topic (232).

An aircrew online health survey reported cancer in aircrew in 5.8% of the 1,020 respondents, with an average age of 41, which is ten times higher than the national average for that age group (26). Several cancers were reported in a small study of UK pilots who worked on the BAe (British Aerospace) 146 aircraft, including two cases of glioblastoma multiforme (GBM) brain tumours (21). The BAe 146 aircraft was acknowledged to have a greater level of oil

leakage than other aircraft types (see ref. 12, pp. 34-35). Another GBM was recorded in a pilot with an extensive history of oil fume exposures (30).

There are various references to substances in the oil, hydraulic and deicing fluids that are suspected of causing cancer in the GHS and CLP chemical classification systems (72-75). Tributyl phosphate (TBP) and 2,6-di-tert-butyl-p-cresol used in many hydraulic fluids are listed as a 'Carc. 2 – suspected of causing cancer' (72-74). Beta naphthylamine (BNA) listed on previous versions of MJO II MSDSs, is reported to be at very low levels, as a contaminant of the commonly used oil additive phenyl-α-naphthylamine (PAN) (78). BNA is a category 1A carcinogen - 'may cause cancer in humans' (72-74). In a similar manner N-2-naphthylaniline (PBN), also listed as a low-level contaminant of PAN, is categorised as a Carc. 2 (72-74).

While MSDS and product labelling varies considerably, as an example the Eastman Skydrol LD4 and PE-5 hydraulic fluid MSDSs state: 'H351*: suspected of causing cancer*', whereas the ExxonMobil HyJet IV-A Plus fails to list this hazard in this manner (218, 219).

Additionally, research published in 2015 describes that low-dose exposures to mixtures of chemicals in the environment may be combining to contribute to environmental carcinogenesis (233). The research found that: 1) cumulative effects of individual low dose (non-carcinogenic) chemicals acting on different pathways and a variety of related systems, organs, tissues and cells could plausibly conspire to produce carcinogenic synergies; and 2) 59% of environmental chemicals reviewed for specific actions on key pathways/mechanisms that are important for carcinogenesis were found to exert low-dose effects, with 30% of these in a non-linear dose-response pattern (233).

Some of the substances in the fluids are classified as possible or suspected mutagens under the REACH/CLP process by the substance registrants (73, 74). The ortho isomers of TCP are listed under the CLP 'notified' database as a 'Muta. 1B -may cause genetic defects', as is 2,6-di-tert-butyl-p-cresol used in some hydraulic fluids, which also carries a 'Muta. 2 - Suspected of causing genetic defects' hazard classification. Ethylene glycol used in some de-icing fluids is also listed as a 'Muta. 1B-May cause genetic defects' (73, 74).

Susceptibility to infection:

Susceptibility to infection is a common complaint among flight crew. In particular, acute and recurrent sinusitis are prevalent (10, 14-16, 22, 25, 26, 28, 30, 57). Systematic insights into appropriate diagnostic methods are missing thus far, but in recurrent cases referral to an otolaryngologist should be considered.

Sleep disturbance:

Changes to sleep patterns are regularly reported by flight crew (15, 16, 21, 24, 25, 28-30), but to date there are no formally published recommendations regarding investigation and management. Persistent cases should be referred for a home-based sleep study or consideration for referral to a sleep disorders specialist.

Visual acuity and eye disorders:

Changes in visual acuity are often reported by air crew (9, 13-19, 21-23, 25, 26, 28, 30, 41, 45, 46, 126). However, to date there are no formally published recommendations regarding investigation and management. Eye disorders reported after fume events have included nystagmus, dry eye, and abnormal eye movements such as saccadic eye movement and convergence insufficiency (234).

The visual system is the most widely distributed system throughout the CNS. Therefore, in diffuse encephalopathies, visual symptoms will usually be among the most common presenting symptoms. This is certainly true, for example, in multiple sclerosis where lesions can arise randomly in any part of the brain and affect the visual system with high probability. It would seem likely that this also applies to the diffuse encephalopathy associated with Aerotoxic Syndrome. Persistent problems should be referred to an ophthalmologist or a neuro-ophthalmologist.

Joint aches and pains:

Joint pain has been reported by flight crew (15-19, 21-26, 28-30, 41, 45, 47) but to date there are no formally published recommendations regarding investigation and management. Persistent problems should be referred to a rheumatologist.

General information:

The oils and hydraulic fluids safety data sheets (SDS) are continually updated, but they vary considerably between manufacturers despite the products being very similar overall. As an example, an oil SDS identifies in section 1 that the intended use of the oil is: *"Aviation lubricating oil, turbine oil"* and states that the product is *"not expected to produce adverse health effects under normal conditions of use"* and the product *"should not be used for any purpose other than the intended use in section 1 without expert advice. Health studies have shown that chemical exposure may cause potential human health risks which may vary from person to person"* (133). Under hazards identification it states: *"this material is hazardous according to regulatory guidelines"* and lists the classifications as *"Reproductive toxicant (fertility): category 2. Specific target organ toxicant (repeated exposure) category 2... Hazard statements: H361: Suspected of damaging fertility. H373: May cause damage to organs through prolonged or repeated exposure: Blood, Kidney. Precautionary statements: ... Do not breath mist/vapors..."* (133). A Shell turbine oil SDS states the oil is to be used as a *"synthetic lubricating oil for aircraft turbine engines... This product must not be used in applications other than those listed... Not expected to be a health hazard when used under normal conditions"* (221).

Toxicity data for Mobil Jet Oil II states, as an example, that the following applies to exposure to PAN (N-phenyl-1-naphthylamine): *"A single oral overexposure may lead to signs of cyanosis, including headache, shallow respiration, dizziness, confusion, fall in blood pressure, convulsions, coma, jaundice. Anemia may occur later. Repeated exposure in laboratory animals caused liver and kidney damage and depression of bone marrow activity. Hematuria may occur due to bladder and kidney irritation. Genotoxic in vitro. .... Undiluted PAN is a skin sensitizer. Human testing with lubricants containing 1.0% PAN caused no reactions indicative of sensitization"* (133).

ExxonMobil advised that breathing turbine oil aerosols or vapours accidentally released into aircraft cabins *"are not what we would refer to as 'normal use'"* but considered such exposures as safe based on their internal and published risk assessments (76, 235). In order to receive more complete information regarding the hazards associated with individual chemicals, it is

advisable to review the GHS chemicals database, or database in a specific region such as the EU CLP classifications list and associated REACH online database (69).

Appendices 9A and 9B outline the hazard classification statements based on the EU CLP chemical classifications regulation (69). These hazard classifications are applicable on a harmonised or notified basis for the substances in the oils, hydraulic and de-icing fluids at or above the levels that attract the various classifications. Other substances in the fluids have hazard classifications, however these are not outlined here because the substances are in the fluids at levels below the classification cut off level. This review does not take into account the hazards associated with the complex mixture created through thermal degradation, pyrolysis or hydrolysis.

SECTION 10: DIAGNOSTIC CODING

Many insurance companies and others require a specific diagnosis according to accepted disease processes recorded in the literature or published diagnostic coding systems, such as the International Classification of Diseases (ICD). These can be accessed at https://www.icd10data.com. Some useful ICD10 diagnostic codes are listed below:

- J68.8: Other respiratory conditions due to chemicals, gases, fumes and vapours.
- J68.9: Unspecified respiratory condition due to chemicals, gases, fumes and vapours.
- T52.8: Toxic effects of other organic solvents.
- T52.9: Toxic effects of unspecified organic solvent.
- T65.9: Toxic effect of unspecified substance.
- G64: Other disorders of the peripheral nervous system.
- G62.2: Polyneuropathy due to other toxic agents.
- G62.9: Polyneuropathy, unspecified.
- F06.7: Mild cognitive disorder.
- G92: Toxic encephalopathy (If the toxic agent is to be indicated an additional code number (Chapter XX) must be used).
- J98.8: Other specified respiratory disorders.
- J98.9: Respiratory disorder, unspecified – (respiratory disease (chronic) NOS).

SECTION 11: EMERGING ISSUES

A. Ultrafine particles:

A growing body of literature now supports that exposure to ultrafine particles or fine particulate matter has various adverse effects on health (236-239). For example, *"exposure to fine particulate air pollution has adverse effects on cardiopulmonary health"* with results having *"important scientific, medical, and public health implications that are broader than debates over legally mandated air quality standards"* (238). This indicates that reliance on exposure standards or thresholds will not be protective for such exposures. Adverse effects associated with air pollution/ultrafine particles have also been reported for cognition (240) and other effects on brain (241), brain development (242), dementia (243), birth weight (244), the lung (245, 246), spontaneous pregnancy loss (247), coronary events (248) and a wide range of non-communicable diseases (249, 250). Exposure to airport and traffic related UFP's may increase the risk of brain, lung and childhood cancers (251-254).

There is little to no dispute among medical scientists that chronic exposure to particulate aerosols is detrimental to health. Epidemiological studies worldwide have consistently demonstrated links between ambient particulate matter exposure and adverse health outcomes, including increased rates of respiratory and cardiovascular illness, hospitalisations and premature mortality (238, 248, 249, 255-257). Particles are usually defined by their size, for example, PM_{10} and $PM_{2.5}$, as the mass of particles with aerodynamic diameters less than 10 or 2.5 µm, respectively. Interest is now also focusing on the fraction of ultrafine particles with a diameter less than 0.1 µm, which are abundant in number but contribute little to the mass (258, 259).

The ultrafine particles are usually measured only for research purposes and are effectively outside regulatory control. It is these emissions that are the main theme of this evidence. Studies have shown that ultrafine particles are more toxic than larger particles (256, 257, 260-262). Furthermore, individual particles have been shown to be capable of inducing inflammation and oxidative stress (262), suggesting that particle number concentrations, which are dominated by ultrafine particles, may be more indicative of some potential health impacts than particle mass concentrations. Ultrafine particles are also important because of their high alveolar deposition fraction, large surface area, ability to induce inflammation, and potential to translocate into the blood circulation system. At a given mass, ultrafine particles (diameter < 0.1 µm) have 10^2 to 10^3

times more surface area than fine particles with diameters in the 0.1– 2.5 µm range and approximately 10^5 times more surface area than coarse particles (2.5 µm < diameter < 10 µm) (263). The surface area to-mass effect may affect the relative toxicity of particles to respiratory systems, in combination with a higher deposition efficiency of ultrafine particles in the alveolar region (264).

An estimated two million excess deaths globally per year are due to particle inhalation, of which approximately 370,000 per year are within the European Union. Health effects are not limited to lung injuries. The deaths also include cardiovascular diseases and cancers (265).

Ultrafine particles up to several hundred thousand particles/cm^3 have been identified in both cabin air (66, 83, 85, 116, 266, 267) studies as well as oil pyrolysis/bleed air studies (80, 82, 92, 93). A 2019 review on aircraft exhaust emissions found that the nanoparticles were dominated by nearly intact forms of jet engine lubrication oil (268). The explanation of how the high temperatures in engines lead to the oils generating UFPs, of which some will enter the cabin air supply, is provided in Howard et al., (57). The continual presence of ultrafine particles over a typical working lifetime in air crew of up to 20,000 hours will predispose them to chronic respiratory problems and will exacerbate the translocation of neurotoxic substances across the blood brain barrier (57). Short term exposures to aviation related ultrafine particles near a major airport were found to be associated with decreased lung function and a prolonged QTc interval in healthy adults (269) [the QTc interval is a measurement on an ECG that is used to calculate the heart rhythm]. With respect to respiratory irritation, jet engine particles were also found to have physicochemical properties, toxicity and adverse effects similar to diesel exhaust particles and other traffic emissions (270, 271). However, this does not apply to neurotoxicity.

B. Autoantibodies against neuronal and glial proteins in blood biomarker testing:

Serum biomarkers such as cytoskeletal proteins, resulting from axonal degeneration, have been used in diagnosing brain injury. These proteins are usually measured in serum shortly after the onset of brain injury because they have short half-lives. Research published in 2017 described how assays have been performed to detect circulating autoantibodies against cytoskeletal proteins, which can be used as biomarkers for brain injury, months or even years after onset (199). Evidence indicates that short-term testing after a fume event can yield information.

Autoantibody biomarkers can be investigated on the day of exposure or months or years after exposure because chronic exposure is assumed for aircrew (28, 57, 59). It would be beneficial to investigate passengers.

In a study of flight crew members reportedly exposed to cabin air emissions containing OPs, elevated autoantibodies to nervous system-specific proteins and possible development of neuronal injury and gliosis were identified (45). The proteins selected represented various types of proteins present in nerve cells that are affected by neuronal degeneration. In the serum samples, immunoglobin G (IgG) was measured using a western blot against neurofilament triplet proteins (NFP), tubulin, microtubule-associated tau proteins (tau), microtubule-associated protein-2 (MAP-2), myelin basic protein (MBP), glial fibrillary acidic protein (GFAP), and glial S100B protein. The study demonstrated a temporal relationship between exposure to air emissions, clinical condition, and level of serum autoantibodies to nervous system-specific proteins (45). Further studies using this protocol have been reported, so this method could be considered in OP neurotoxicity (199, 272).

While testing for autoantibody biomarkers is not currently routinely available, it would require a specialist laboratory to initially establish this protocol. This would enable sera isolated from samples to be assayed for the presence of autoantibodies against 10 neuronal and glial proteins that indicate markers of central nervous system (CNS) injury. Further text and methodology in Appendix 8 will provide additional information for the autoantibodies against neuronal and glial proteins in blood biomarker testing.

C. Increased genetic susceptibility to toxic compounds:

Inter-individual variability in response to pharmaceuticals and toxic substances is the norm. For example, there are defined genetic polymorphisms which influence aldehyde dehydrogenase activity, significantly affecting individual tolerance for the effects of ethyl alcohol (273).

The same is the case for organophosphate compounds, although much more complicated. Genetic variability and levels of expression of genes involved in the detoxication of organophosphorus compounds (OPs) such as insecticides, nerve agents, jet engine anti-wear

agents and hydraulic fluids contribute to the variability in sensitivity to exposures to these compounds. Inter-individual genetic variations in the ability to metabolise certain chemicals, together with the effects of diet and medications that influence enzyme activity (113, 274, 275), may explain why some aircrew and passengers develop symptoms even at low doses, whereas others undergoing the same fume event may remain asymptomatic.

The key enzymes that influence individual response to OP exposures include cytochromes P450s (especially CYP450 3A4), carboxylesterase, butyrylcholinesterase (BChE) and paraoxonase-1 (PON1). Key enzymes that protect against increased oxidative stress associated with OP exposures include glutathione S-transferases, superoxide dismutases, and PONs 1, 2, and 3 and others.

With respect to genetic variations in levels/activities of specific proteins that may influence an individual's response to specific OP compounds, it is important to examine the given variations in detail. Effects measured *in vitro* will not necessarily translate into the same effects *in vivo*. A good example is the PON1 genetic variant which inactivates paraoxon (the neurotoxic metabolite of the OP insecticide parathion) via hydrolysis in a test tube. One isomer (Arginine-192; $PON1_{R192}$) of PON1 does so at a rate approximately seven times faster than another (Glutamine-192; $PON1_{Q192}$) (276), suggesting that the faster variant is more protective. However, neither variant protects against paraoxon exposure because the *in vivo* rates of inactivating paraoxon are not sufficient to protect from ill effects (277). The only OP exposures that PON1 has been clearly shown to protect against to-date are the active metabolites of chlorpyrifos and diazinon. There is little, if any, evidence that PON1 hydrolyses the triaryl phosphates added to jet engine oils; for example, mice which were genetically engineered to have no PON1 enzyme activity at all did not react to TCPs differently than mice with fully functional PON1 activity (unpublished results[2]).

Some proteins protect by stoichiometric binding to OP compounds, such that higher levels are more protective. This is relevant to carboxylesterase, for example, which varies by at least 18-fold in humans (278). Also, inter- and intra-individual variations in the levels of BChE have also been defined (279). These enzymes are recognised to have a protective role in modulating the effects of OP exposures, but the specific effects of gene mutations and variations of activities of specific

---

[2]. Furlong, C, Cole T. Dept. Medicine – Div. Medical Genetics, University of Washington. (2015).

proteins, must be defined for each specific OP of concern because effects are unlikely to be the same.

Finally, it is possible that high levels of enzymes that modulate the oxidative stress associated with exposure to specific OPs (e.g. glutathione synthetase, glutathione transferases, PONs 1, 2 & 3 and other enzymes involved in modulating oxidative stress) may provide some protection against exposures. The CYP450 3A4 enzyme converts several of the triaryl phosphates into metabolites that are potent inhibitors of physiologically crucial enzymes (unpublished results[3]). The activity and levels of CYP450 3A4 can be significantly increased or decreased by both diet (specific foods and drugs) and mutations in either the gene encoding P450 3A4 or numerous genes which encode proteins that regulate the levels of P450 3A4. Genetically based diverging levels of CYP 450 3A4 indicate differences in individual hepatic activity of 40 fold (278). It is important to know whether high levels of CYP 450 3A4 increase or decrease sensitivity to specific TAPs. If high levels are detrimental, naringenin, a compound found in grapefruit is a simple and safe way to inhibit the activity of CYP450 3A4 (113). If high levels are protective, compounds that induce very high levels of CYP450 3A4 can be administered. The former seems the most probable. There are protocols for measuring the activity of an individual's CYP 450 3A4 [e.g. (278)]; however, diet, medications and environmental conditions will cause the activity to vary over time, so a spot activity test result will not necessarily reflect the activity at the time of exposure.

In summary, inter-individual genetic and dietary differences can be expected to influence susceptibility to the ill effects of OPs in engine oils and hydraulic fluids, whether those effects follow chronic low dose repeated exposures, a higher dose "fume event," or a combination of the two. The precise mechanisms will need to be validated through animal studies [e.g. (277, 280)] or, when possible, through cell culture model systems.

C. Low-level repeat exposure to mixtures:

There is growing understanding that low-level exposure to mixtures may react very differently to exposure to a single acute level of an individual substance. The FAA medical division highlighted that "*it is not so simple to adjudicate and predict the toxicity caused by the constituents and the pyrolytic products of engine oil, hydraulic fluids, and lubricant*" (99). It also reported that "*substances*

[3]. McDonald, M - Medicinal Chemistry, University of Washington, 2015.

that are not toxic individually may become highly toxic within a pyrolysed mixture." and *"the chemicals found in the carbonaceous material may not necessarily be individually toxic at the found concentrations, but if they are mixed together at those concentrations, the mixture might be highly toxic... The issue of the interaction of chemicals in regard to the toxicity of mixtures has apparently not been fully addressed... because of the complexity, the best approach to resolve this toxicological and aviation safety issue would be preventative 33[sic]*(61) *– that is to minimize oil leaks into bleed air..."* (99).

A European Commission-funded study identified the need for: *"precautionary actions on the assessment of chemical mixtures even in cases where individual toxicants are present at seemingly harmless concentrations"* (164). A UK government study reported that *"current risk assessment practices are largely based on evaluating the toxicity of single chemicals... It is conceivable that risk assessments based upon single substance evaluations may underestimate the toxicity of a mixture... This could occur through exposure to multiple chemicals that cause the same effect ...[or] it is possible that interactions between chemicals may change the dose response relationships observed for chemicals tested in isolation"* (165). A 2018 EU policy statement identified that *"combined exposure to multiple chemicals can lead to health/environmental effects even if single substances in the mixture do not exceed safe levels"* (166). An EU/EASA funded oil pyrolysis study reported that the *"possible effects relating to mixture toxicology need further investigation"* (80).

Exposure to mixtures of certain OPs was shown to generate synergistic neurotoxicity by a direct mechanism at the cellular level, thus increasing the toxicity (195). Additionally, it is reported that *"known synergistic effects between organophosphates and pyrethrins, based on carboxylesterases inhibition, can be expected in the presence of Tricresylphosphates (TCPs)"* (281).

D. Chronic low-level exposure to OPs:

Evidence suggests that repeat low level exposure to OPs is distinct from acute single exposures. Terry (186) reports that *"There is now substantial evidence that this canonical (cholinesterase-based) mechanism cannot alone account for the wide variety of adverse consequences of OP exposure that have been described, especially those associated with repeated exposures to levels that produce no overt signs of acute toxicity. This type of exposure has been associated with prolonged impairments in attention, memory, and other domains of cognition, as well as chronic illnesses where*

these symptoms are manifested (e.g., Gulf War Illness, Alzheimer's disease)."

As an example, with regard to a different OP, Naughton et al., reported that "*results indicate that repeated exposures to the nerve agent, DFP at doses that are below the threshold for acute toxicity, can result in alterations in myelin structure and persistent decreases in axonal transport in the rodent brain. These observations could explain some of the long-term neurological deficits that have been observed in humans who have been repeatedly exposed to OPs... In addition, repeated exposures to the OP, tricresyl phosphate (TCP), used as an anti-wear additive to jet engine oil, has been implicated in 'Aerotoxic Syndrome'*" (282). Studies in the past decade (45, 199, 272) "*appear to support the argument that OP exposure may lead to the generation of autoantibodies that target proteins known to play critical roles in both the structure and function of neurons including myelination and axonal transport. An "autoimmune" response might offer one explanation for why OP exposures could lead to chronic (in some cases lifelong) symptoms*" (282). Further information is available in Section 4 – Target organ toxicity of the nervous system.

Axelrad et al., (196) identified that repeat very low dose exposure to certain OPs on cells increased the susceptibility (reduced the threshold for toxicity) to neurotoxic damage upon further higher dose exposure.

E. Acute versus chronic exposure:

The aviation industry and associated inquiries have undertaken a range of investigations over the years. These include not recognising Aerotoxic Syndrome as a diagnosis, because symptoms vary too widely and are not consistent (54), and that there is no causal association between cabin air exposures and ill health (283). A report published in 2013 stated that "*contamination of cabin air by components and/or combustion products of engine oils, including triaryl phosphates, does occur ... Episodes of acute illness, sometimes severely incapacitating, have occurred in temporal relation to perceived episodes of such contamination*" (110). The UK CAA now reports that "*It is acknowledged that people who experience a fume event (of any type) may report symptoms such as irritation to the eyes, nose and throat. These symptoms usually resolve... Long term ill health due to any toxic effect from cabin air is understood to be unlikely, although such a link cannot be ruled out*" (65). The EU Commission FACTS air quality research tender stated, "*whilst a causal association between cabin air contamination by oil mists and ill-health in commercial aircrew could not be identified, a number*

of incidents with a temporal relationship between CAC event reports and acute ill-health effects indicated that such an association was nevertheless plausible" (284).

While there has been a continuing reliance on individual substances by industry and governments, the toxicity assessment of complex mixtures has been disregarded to date and therefore the short and long-term effects have often been dismissed. The UK CAA has continued to rely upon individual substances and therefore questions the adverse effects that may be possible, when stating: *"From what is currently known about the concentrations of potentially toxic chemicals in contaminated air, long-term toxic effects would not be expected, but this remains an area of scientific uncertainty"* (52). We believe the references and evidence cited in this document go a long way to explaining the causal mechanism.

F. Dose:

The constant presence of a low-dose complex mixture of fugitive engine emissions is established. A common argument employed is that the concentration of the constituents of this aerosol are too low to cause any effect. However, this argument contains an (unwritten) assumption that any possible adverse effect would be totally reversible. Such an assumption is contradictory to the published literature on chronic low dose exposure to OPs. One of the main consequences of chronic low dose repeated exposure reported by Terry (186) is interference with axonal transport. One of the functions of anterograde axonal transport is to deliver neurotrophins to the postsynaptic neurons. Neurotrophins are essential for the continued healthy functioning of neurons and chronic perturbing of their delivery can have pathological consequences.

The dosing regimen of toxic substances must be considered, particularly in the case of the nervous system. Acute high dose exposure can have acute neurotoxic consequences – an example would be accidental exposure to pesticides. However, repeated low dose exposure to OPs has also been demonstrated to have measurable neuropathological consequences in the case of "dipper's 'flu" in farmers applying OPs with sheep dips (198).

Most of the oil and OP studies have been undertaken on an ingestion or dermal basis. However, *"Chemicals tend to be more toxic by the inhalation route than by the oral route due to rapid absorption and distribution, bypassing of the liver's metabolic protection (portal circulation), and potentially*

serious portal-of-entry effects, such as irritation, edema, cellular transformation, degeneration, and necrosis. An inhalation risk assessment that is based on oral data generally underestimates the inhalation risk because it cannot account for these factors" (285).

In toxicology, it is standard practice to assume that 100% of inhaled substances are retained and can pass directly to the systemic bloodstream without passing through the liver and therefore count towards the 'dose'. It is typical among aircrew to log in excess of 20,000 hours flying time in a career. Over that period, a person would typically inhale 9,000 cubic metres of air (nine million litres). By looking at the various reported levels of OPs in the literature it is possible to make an estimate of their internalised dose during a working lifetime as shown in Table 2. The concentrations listed and therefore the estimated dose, will be based on the amount of vapour present. However, this is likely to be an underestimate due to recent research which has shown that much of the internalised dose will be in the form of nano sized oil droplets (185, 268). It must be noted that this does not address the amount of the total mixture (which comprises more than 100 chemicals) internalised.

The maximum levels identified could, in some cases, be associated with low-level permissible oil consumption in normal operations or failure conditions. These exposures would not be applied to the whole 20,000-hour career but provide an understanding of dose.

Assumptions in Table 2:

Dose = concentration x volume

TCP (mixed isomers) dose

Table 2: Internalised dose during crew working lifetime

	Study<br>A (maximum)<br>B (mean)	Conc.<br>µg/M3<br>(TCP)	Vol M3	Dose mg	Notes
A	Cranfield, 2011<br>(83)	37.7	9000	339	Minor fume events noted by researcher in 25% of flights.<br>(Assumed incorrectly to be minor and not reportable)
B	Cranfield, 2011	0.22	9000	1.9	As above

**A**	EASA, 2017 (66)	1.51	9000	13.6	No fume event/ oil leakage identified-T-CAC
**B**	EASA, 2017	0.009	9000	0.081	No fume event
**A**	Honeywell/Malmo, 1999 (122, 286, 287)	20.3	9000	183	Fume event - pilot incapacitation
**A**	Rosenberger*, 2018 (288)	0.981	9000	8.8	Fume event/ diversion in 1 of 17 flights.
**B**	Rosenberger*, 2018	0.065	9000	0.58	Fume event/ diversion in 1 of 17 flights.
**A**	TNO, 2013 (109, 289)	0.155	9000	1.4	No fume events
**B**	TNO, 2013	0.0069	9000	0.062	No fume events

* Averaged over 17 flights

G. Endocrine disruptors:

The literature reports that some OP flame retardants can act as endocrine disruptors (ED). OPs have potentially wide-ranging effects on the oestrogen receptor, androgen receptor and glucocorticoid receptor, amongst other receptors (290, 291). Endocrine disruptors act by interfering with the action of receptors by enhancing or blocking the activity of the naturally occurring ligand, for example hormones such as oestrogen or testosterone. The dose-response is usually highly non-linear. Long term effects are difficult to predict but can include cancer induction. The link between breast cancer and xeno-oestrogens, for example, is well established (292). TCP as an example has been recognised to have oestrogen-disrupting effects (290, 291, 293).

H. Causal connections:

Bradford Hill's still widely used seminal paper of 1965 (294) focuses on how we can move from an observed association to a robust causal inference. The paper identified nine 'features' (often misnamed as 'criteria') of the available, and often 'ragged', evidence (295, 296), which, if present, could help justify a robust causal inference. Bradford Hill was careful to point out that if these features of the evidence (Table 3) (297) were absent, then that did *not* justify concluding that the agent being evaluated was *not* causing harm. In other words, the features of the evidence were *asymmetrical*, a word he did not use despite making the conceptual point very explicit when discussing several of the features of the evidence (252).

Bradford Hill would have approached the evidence with: "*the decisive question... whether the frequency of the undesirable event B will be influenced by a change in the environmental feature A?*" (297). And he would have reminded us that an observed association "*may be new to science, or medicine, and must not therefore be too readily dismissed as implausible or even impossible*" (297).

It is reasonable to conclude, from Table 3, that the overall weight of evidence is suggestive of a causal link between aircraft cabin toxic contamination and health effects in some crew and passengers. The link is more likely than not i.e., at or around the "*balance of probabilities*", or the "*fair*" strength of evidence (297), which Bradford Hill considered a sufficiency of evidence to justify preventative measures.

Table 3: The Bradford Hill approach applied to Aerotoxic Syndrome (2017)

Data source:(297)

**Strength of association:** Case studies and clinical data indicate clear health impacts in significant proportions of exposed groups.
**Consistency:** Clinical data consistent with known toxic effects of organophosphates; and across varying aircraft types/countries.
**Specificity:** Aerotoxic Syndrome is a syndrome (as is Acquired Immune Deficiency Syndrome; Multiple Chemical Sensitivity; Occupational Asthma; Gulf War Syndrome and Asperger's Syndrome) and with common neurological/respiratory symptoms linked to oil leakage/pyrolysis products exposure in cabin air.
**Temporality:** Aerotoxic Syndrome was never reported prior to the introduction of engine bleed air pressurization systems and cabin air contamination precedes linked health effects.
**Biological gradient:** High contaminant exposure often causes greater health effects; but low dose effects also apparent, suggesting non-linearity
**Plausibility:** The known effects of organophosphates and other cabin air contaminants support a causal link.

Coherence: Animal and human data support a causal link.

Experiment: Some health effects are reversible after exposure cessation, especially for acute exposures.

Analogy: Polychlorinated biphenyls; hot rubber fumes; welding fumes; traffic fumes, occupational asthma, leaded petrol, methyl mercury, organophosphate pesticides and tobacco smoke have relevant features.

Further supportive causation evidence is contained in the paper '*Neurotoxicology: what the neurologist needs to know*' is authored by Professors Harris and Blain, two internationally recognised and highly regarded neurotoxicologists (190); in particular the last paragraph of the section on page 2, entitled 'Exposure, Concentration, and Duration of Exposure'. It states: *"High doses of a toxic chemical will give rise to an acute toxic response, but prolonged exposure to low concentrations of a toxin may only cause a slowly developing chronic response. The circumstances of exposure and the toxicity of the toxin will determine which of these is the more serious"* (190).

The statement that Harris and Blain make here is common sense as well as being supported by hundreds of observational studies. We consider that this aspect of prolonged exposure of aircrew (as distinct from passengers) is a significant feature in the aetiology of the pattern of illness being manifested. It is made complicated by the fact that exposure is to a complex mixture of compounds and not a single chemical. It is clear that occupational exposure limits, apart from being inappropriate for application to the general public and also not applicable at altitude or for complex heated mixtures, do not address exposure to toxic agents for the many thousands of hours that air crew experience.

Further on in their paper, Harris and Blain quote five cardinal signs on causation in neurotoxicology propounded by Schaumburg (190, 298):

(1) Presence of the suspected agent is confirmed by history and either environmental or clinical chemical analysis.

(2) Severity and temporal onset of the condition are commensurate with duration and level of exposure.

(3) The condition is self-limiting and clinical improvement follows removal from exposure.

(4) Clinical features display a consistent pattern that correspond to previous cases.

(5) Development of a satisfactory corresponding experimental in vivo or in vitro model is absolute proof of causation.

With respect to:

Point (1) the presence of fugitive emissions in engine bleed air has been demonstrated on many occasions.

Point (2) is supported by clinical data (28).

Point (3) there is some evidence that the medical condition of air crew affected by Aerotoxic Syndrome can improve after removal from the cabin environment, though usually slowly and rarely completely.

Point (4) there is a consistency to the presenting symptomatology (28).

Point (5) there is ample experimental evidence of the toxicological damage caused by repeated low-dose exposure to OPs (186, 187).

It is important to recognise that, where specific symptoms are identified in this document as being in the literature or associated with a specific substance, the latter may not be the only substance to cause such effects. It is necessary to look at the broader picture because the effects may have been caused not only by the substance in question, but also by other substances or in combination with other substances.

SECTION 12: CONCLUSIONS

Pyrolysed or thermally degraded engine oil fumes contaminating the aircraft cabin air conditioning systems has been recognised and well documented since the 1950s. It is now clear that inhalation of these potentially toxic fumes causes ill health. These are well documented, although not widely accepted, particularly by the airline industry and its regulators. Symptoms of ill health are temporally associated with fume exposure in most cases, but it is now clear that cumulative exposure to regular small exposures are also damaging and may be prompted by a single exposure to an acute dose. Although organophosphates have been the main subject of interest, fumes also contain a complex mixture of VOCs and UFPs, so considering the toxicity of individual substances in such complex heated mixtures has limited value.

The insufficient recognition of illness caused by exposure to pyrolysed engine oil, de-icing and hydraulic fluids in bleed air is likely to be due to a lack of knowledge and clinical acumen, and to finding more clinically recognised diagnoses in unexplained clinical presentations. Based on a more probable than not approach using the Bradford Hill perspectives on causation, illness caused by fume event exposures is a real clinical entity. There is a need for a systematic and consistent approach and education of all professions involved.

This document is a consensus approach that has been written by internationally recognised experts. It has been compiled to help recognise, investigate and manage persons suffering from the toxic effects of inhaling pyrolysed engine oil and other fluids contaminating the air conditioning systems in most aircraft.

APPENDICES

Appendix 1A: International Civil Aviation Organization (ICAO): Examples of potential types of aircraft fumes.

Data source (55).

Table 1. Examples of potential types of fumes

Potential contaminants in the ventilation supply air	De-icing and/or anti-icing fluid<br>Electrical faults<br>Engine compressor wash<br>Engine oil<br>Exhaust (aircraft or ground vehicles)<br>Fuel<br>Hydraulic fluid<br>Recirculation fan failure
Items in the cabin and/or flight deck that can be sources of fumes	Carry-on baggage<br>Cleaning products<br>Disinfectants<br>Disinsectants<br>Food items<br>Galley equipment<br>Lavatories

Appendix 1B: American Society of Heating, Refrigerating and Air-Conditioning Engineers (ASHRAE) Guideline 28-2012: Air quality within commercial aircraft.

Data source (60).

TABLE 8.1.1 Subset of Contaminants that Could Be Present in the Aircraft Cabin Environment

Contaminant or Group of Contaminants	Possible Sources*	Examples
Carbon monoxide	A, B, D, E	
Carbon dioxide	G, H	
Ozone	J	
Ultra fine PM	A, B, C, E, G	
$PM_{2.5}$	C,H, B, E	
PM_{10}	C, H	
Aldehydes	A, B, E, F,H	Formaldehyde, acetaldehyde, acrolein, valeraldehyde, butyraldehyde, benzaldehyde, crotonaldehyde, hexaldehyde, propionaldehyde, nonanaldehyde
Organophosphates	B, E, I	All tricresylphosphate isomers, tributyl phosphate
Carboxylic acids	B, F	Pentanoic acid, heptanoic acid, octanoic acid
Aromatics	A, B, F, I	Benzene, toluene, ethylbenzene, xylene, styrene, naphthalene, etc.
Alkanes	A, F, I, B, E	Heptane, hexane, octane, nonane, decane, 3-methylpentane, 2-methyl hexane, 3-methylhexane, 1-methylcyclohexane
Amines	B, F, E	N-phenyl-1-napthylamine
Ketones	B, H	Methylethylketone, acetone
Esters	B, E	ethyl acetate, butyl acetate
Pyrethroids	I	Permethrin/phenothrin
Alcohols	A, D, H	Ethylene glycol, propylene glycol, ethanol, methanol

* Possible sources: A = fuel, B = oil, C = equipment wear, D = deicing fluid, E = hydraulic fluid, F = anticorrosion coating, G = galley, H = occupants, I = pesticides, J = outside air (Outside air is a possible source for most contaminants, particularly when on the ground or at low altitudes.)

Appendix 1C: Frequency of occurrence of contaminants in bleed air samples.

Data source: (89).

U.S. Patent Feb. 27, 2018 Sheet 6 of 21 US 9,902,499 B2

COMPOUND	NON-ZERO SAMPLE SIZE	TOTAL SAMPLE SIZE	FREQUENCE OF OCCURANCE (%)-NON-ZERO SAMPLE SIZE/ TOTAL SAMPLE SIZE
ACETONE (2-PROPANONE)	191	195	0.98
ACETALDEHYDE (ETHANAL)	264	282	0.94
TRICHLOROFLUOROMETHANE	194	214	0.91
FORMALDEHYDE	257	281	0.91
TOLUENE	184	214	0.86
CARBON MONOXIDE	189	255	0.74
M-XYLENE (1,3-DIMETHYLBENZENE)	155	214	0.72
O-XYLENE (1,2-DIMETHYLBENZE)	155	214	0.72
TOTAL XYLENE	155	214	0.72
2-BUTANONE (METHYL ETHYL KETONE)	136	215	0.63
CHLOROMETHANE	124	214	0.58
TCP ISOMERS- MIXED	53	94	0.56
OCTANE	118	214	0.55
N-UNDECANE	118	214	0.55
N-DECANE	117	215	0.54
BENZENE	114	216	0.53
ACETONITRILE	102	213	0.48
TRICHLOROTRIFLUOROETHANE	96	213	0.45
2-PROPANOL (ISOPROPYL ALCOHOL)	95	215	0.44
N-DODECANE	90	215	0.42
BENZALDEHYDE	118	282	0.42
ETHYL BENZENE	87	214	0.41
PROPIONALDEHYDE (2-PROPYNAL)	111	279	0.4
N-NONANE	84	215	0.39
TETRACHLOROETHENE (PERCHLORETHYLENE)	81	215	0.38
BUTANAL (BUTYRALDEHYDE)	106	281	0.38
1,2,4-TRIMETHYLBENZENE (PSEUDOCUMENE)	80	216	0.37
1,3,5-TRIMETHYLBENZENE (MESITYLENE)	80	216	0.37
ETHANOL (ETHYL ALCOHOL)	76	212	0.36

(2) continued to FIG. 3B

FIG. 3A

(2) continued to FIG. 3B

continued from FIG. 3A

2

continued from FIG. 3A

2

CARBON DISULFIDE	68	216	0.31
PENTANAL (VALERALDEHYDE)	87	282	0.31
2-METHYLBUTANE	57	215	0.27
MTBE	47	215	0.22
PHENOL	26	125	0.21
CROTONALDEHYDE (TRANS-2-BUTENAL)	60	282	0.21
PHENANTHRENE	19	95	0.2
N-HEPTANE	41	213	0.19
PENTANE	40	216	0.19
METHYLCYCLOPENTANE	35	215	0.16
HEXANAL	32	215	0.15
1,1,1-TRICHLOROETHANE	31	215	0.14
STYRENE (VINYL BENZENE)	28	215	0.13
VINYL ACETATE	28	216	0.13
BUTANE	26	216	0.12
3-METHYLHEXANE	23	214	0.11
N-NONANAL	19	215	0.09
2-METHYLPENTANE	17	214	0.08
ETHYL ACETATE	15	215	0.07
2-HEXANONE (METHYL N-BUTYLKETONE)	14	215	0.07
N-OCTANAL	15	216	0.07
PROPANE	13	215	0.06
CHLOROETHANE	14	216	0.06
N-HEPTANAL	14	216	0.06
NAPHTHALENE	14	216	0.06
CYCLOHEXANE	11	213	0.05
ACROLEIN (2-PROPENAL)	6	194	0.03
P-CYMENE	7	216	0.03
TOCP	1	100	0.01
ISOBUTYRALDEHYDE	2	216	0.01
ISOVALERALDEHYDE	1	282	0.01

FIG. 3B

Appendix 2: Aerotoxic Syndrome – List of symptoms: Acute & Long-term.

Data source: (28).

TABLE 3. OVERVIEW SURVEY

SYMPTOM	Study A		Study B		CLP- Hazard clasification	Literature[e]	Hazard Databases[e, f]		HYPOXIA	HYPERVENTILATION
	n=142		15 incidents		(harmonized/notified)					
	No of pilots reporting Symptoms		No of incidents/ symptom		- oil, hydraulic, deicing fluid		- oil, hydraulic, deicing fluid			
	Acute	Chronic	Acute	Chronic			Acute	Chronic		
NEUROLOGICAL						X	X	X		
CENTRAL (CNS)						X	X	X		
Incapacity/paralysis; Impaired/loss of consciousness	9 (6%)	1 (1%)	15 (100%)			X	X	X	loss consciousness	semi consciousness
Headache /Pressure in head/ trouble speaking	47 (33%)	21 (15%)	11 (73%)	5 (33%)		X	X	X	headache	headache
Balance problems/erratic movement/ataxia	11 (8%)	7 (5%)	2 (13%)	1 (7%)		X	X	X		
Vision problems/tunnel or double vision/dilated pupils/ nystagmus	11 (8%)	10 (7%)	8 (53%)	1 (7%)		X	X		unresponsive pupils	visual disturbances
PERIPHERAL (PNS) Motor;Sensory;Autonomic						X	X	X		
Shaking/tremors; Inco-ordination/motor response	12 (9%)	17 (12%)	11 (77%)	6 (40%)		X	X	X		shakes/ twitches
Paraesthesiae/numbness in limbs/other; Peripheral neuropathy	12 (9%)	25 (18%)	5 (33%)	7 (46%)	Neurotoxicity, Single & repeat exposure	X	X	X		tingling/ numbness
Sweaty/temperature control/ pallor/flushing/taste	7 (5%)	6 (4%)	3 (21%)	6 (60%)		X	X	X	sweating	sweats/hot/ cold
NEUROBEHAVIOURAL						X	X	X		
NEUROLOGICAL						X	X			
Discomfort/intoxication/disorientation/ confusion	16 (11%)	3 (2%)	10 (66%)	1 (7%)		X	X		confusion/ disorientation	confusion/ disorientation
Behavioural/personaility Changes;unreality/anxiety/ depression	1 (1%)	20 (14%)		7 (46%)		X	X	X	unreality	unreality/ anxiety
Dizziness/light-headedness/ lethargy/drowsiness	21 (15%)	9 (6%)	11 (73%)	3 (20%)	Drowsiness/ dizziness:CNS	X	X	X	light headedness	light headedness
COGNITIVE						X	X			dizziness
Cognitive problems: problem solving/concentration/memory/writing	46 (32%)	58 (41%)	14 (93%)	9 (60%)		X	X	X	cognitive problems	cognitive impairment

TABLE 3. OVERVIEW SURVEY

SYMPTOM	Study A		Study B		CLP- Hazard clasification	Literature [e]	Hazard Databases [e,f]		HYPOXIA	HYPERVENTILATION
	n=142		15 incidents		(harmonized/notified)					
	No of pilots reporting Symptoms		No of incidents/ symptom		- oil, hydraulic, deicing fluid		- oil, hydraulic, deicing fluid			
	Acute	Chronic	Acute	Chronic			Acute	Chronic		
Giggling/euphoric			2 (13%)			X	X		euphoria	
GASTROINTESTINAL							X	X		
Nausea/vomiting/ Diarrhoea	25 (18%)	14 (10%)	14 (93%)	5 (33%)	Harmful if swallowed	X	X	X	nausea/ vomiting	
Cramps/bloating/pain/digestive problems		2 (1%)	4 (27%)	2 (13%)	Harmful/ fatal if inhaled	X	X	X		bloating/ belching
RESPIRATORY					Respiratory tract irritant					
Breathing problems/cough/ chest discomfort/wheezing/ lung irritation	15 (11%)	34 (23%)	11 (73%)	4 (27%)	Respiratory sensitization Allergy/ asthma / difficulty breathing	X	X	X	breathing problems	breathing problems
CARDIOVASCULAR										chest pain
Chest pain/ tightness/variable heart rate/palpitations/BP	6 (4%)	21 (14%)	5 (33%)			X	X		variable heart rate/ palpitations	irregular heart rate/ palpitations
GENERAL: rheumatological; miscellaneous; soft tissue					Target organ toxicity-single/ repeat					
Joint/muscle pain/aches/ twitches/weakness	8 (6%)	23 (16%)	4 (27%)	2 (13%)	Liver; urinary tract; heart;	X	X	X		weakness
Feeling unwell/performance decrement	33 (23%)	54 (38%)	15 (100%)	3 (20%)	Respiratory; systemic; CNS	X				
Fatigue/chronic fatigue/exhaustion	27 (19%)	65 (46%)	3 (20%)	6 (40%)	blood; kidneys	X	X	X	fatigue	exhaustion
Chemical sensitivity	3 (2%)	13 (9%)		5 (33%)		X				
Vocal/nasal/ throat polyps/ swelling			1 (7%)	1 (7%)		X				
IRRITATION										

TABLE 3. OVERVIEW SURVEY

SYMPTOM	Study A		Study B		CLP- Hazard clasification	Literature[e]	Hazard Databases[e, f]		HYPOXIA	HYPERVENTILATION
	n=142		15 incidents		(harmonized/notified)					
	No of pilots reporting Symptoms		No of incidents/ symptom		- oil, hydraulic, deicing fluid		- oil, hydraulic, deicing fluid			
	Acute	Chronic	Acute	Chronic			Acute	Chronic		
Eye, nose, throat & voice irritation/burning/redness/ hoarseness	41 (29%)	14 (11%)	15 (100%)	3 (21%)	Eye irritation	X	X	X		dry mouth
SKIN					Skin irritant/					
					skin sensitization					
Skin reaction/blisters/rash (uncovered areas); Burning scalp/alopecia	7 (5%)	11 (8%)	4 (27%)	5 (34%)	Harmful-skin exposure	X	X	X	bluish/red skin	
IMMUNE SYSTEM					Genetic defects	other	other	other		
Recurrent respiratory tract infections/altered immune system	11 (8%)	12 (8%)		1 (7%)	Damage: fertility/ unborn	X				
CANCERS		9 (6%)		1 (7%)	Carc 1B/2 - bladder; liver	X		X		

e X indicates symptom present.
f Hazardous Substances Data Bank, International Chemical Safety Card, US National Institute for Occupational Safety and Health.

Appendix 3: Aerotoxic Syndrome symptoms.

Data source: (27).

Table 2 Aerotoxic syndrome: short- and long-term symptoms

Short term exposure	Long term exposure
Neurotoxic symptoms: blurred or tunnel vision, nystagmus, disorientation, shaking and tremors, loss of balance and vertigo, seizures, loss of consciousness, parathesias;	*Neurotoxic symptoms*: numbness (fingers, lips, limbs), parathesias;
Neuropsychological or Psychotoxic symptoms: memory impairment, headache, light-headedness, dizziness, confusion and feeling intoxicated;	*Neuropsychological or Psychotoxic symptoms*: memory impairment forgetfulness, lack of coordination, severe headaches, dizziness balance, sleep disorders;
Gastro-intestinal symptoms: nausea, vomiting;	*Gastro-intestinal symptoms*: salivation, nausea, vomiting, diarrhoea;
Respiratory symptoms: cough, breathing difficulties (shortness of breath), tightness in chest, respiratory failure requiring oxygen;	*Respiratory symptoms*: breathing difficulties (shortness of breath), tightness in chest, respiratory failure, susceptibility to upper respiratory tract infections;
Cardiovascular symptoms: increased heart rate and palpitations;	*Cardiovascular symptoms*: chest pain, increased heart rate and palpitations;
	Skin symptoms: skin itching and rashes, skin blisters (on uncovered body parts), hair loss;
Irritation of eyes, nose and upper airways.	*Irritation* of eyes, nose and upper airways;
	Sensitivity: signs of immunosuppression, chemical sensitivity leading to acquired or multiple chemical sensitivity
	General: weakness and fatigue (leading to chronic fatigue), exhaustion, hot flashes, joint pain, muscle weakness and pain.

Appendix 4: AIRCRAFT CABIN FUME EVENTS – PROCEDURES TO FOLLOW

Event, clinical history and physical examination

Fume events in aircraft cabins are well recognised and may cause ill health. Clinical illness caused by fume event exposures is increasingly recognised as the Aerotoxic Syndrome.

Most individuals report that the onset of symptoms is time correlated with a flight or immediately after, in some cases necessitating prompt medical examination.

A very broad spectrum of clinical symptoms has been reported to be caused by air supply generated fumes exposure. It is important to recognise and record all symptoms and complaints as they will be helpful to subsequent medical staff who may be consulted.

The onset or recurrence of symptoms may follow one of several different time patterns as follows:

- **In flight:** Ill health developing during the course of flight.
- **Immediate post flight:** Ill health developing immediately after a flight.
- **Late/subsequent:** Ill health developing or continuing days, weeks or even months, years after flight.

This booklet has been designed to assist those persons who have experienced ill health following a fume event and to prompt the recording of what happened, when, individual experiences, symptoms of ill health and other observations. This booklet is intended primarily for use at the time of the fume event or shortly thereafter. For this reason, the prompts related to in-flight and immediately post-flight experiences are highlighted. Longer-term suggested investigations are also listed. Please record all observations and symptoms of ill health in as much detail as possible.

A detailed record of the fume event itself with details of technical and engineering follow up, together with a record of the symptoms and the medical management of afflicted persons are indispensable for longer-term medical management.

**Name**	**Contact**

Date of birth	Other

1A. In flight (air or ground)

- Expected to be undertaken by non-medically trained personnel.
- If medical help is available, also collect and record data listed under 1B – physical examination, as shown below.

Record of environment

Type of aircraft.	
When did event occur (in-flight, stage of flight, on ground, ascent, descent)?	
Where did event occur (where in the aircraft or most likely location)?	
How long did the event continue?	
What happened (e.g. smell, fumes, smoke)?	
If smell or fumes, describe type of smell.	
How many persons affected and status (e.g. pilot, cabin crew, passenger? How many (x out of y) affected, when and for how long?	
Record air quality monitor recordings (if available) / maintenance history / previous events if known.	

Medical record /first aid response

(Record to be made by those assisting the afflicted crew members and other persons.)

Clear detailed and careful description and severity of the fume event experienced by the individual.	
Record symptoms and progression of symptoms.	
Record observations of others (who – important in assessment of affected persons).	
Measure and record oximetry, if available, before oxygen administration	
Record any treatment given used (e.g. was oxygen used? including flow rate, method of administration e.g. nasal cannula/mask, when and duration)<br><br>Other first aid?	
Record any treatments for past exposures, if known.	
Record any unusual behaviour.	
Record past medical history in brief (where relevant), medication etc.	
Covid 19 exposure history?	

Clinical examination

It is expected that trained health care professionals will not be present to conduct a medical examination. However, any observations or physical findings or behaviours should be recorded as they will be helpful to future medical carers in guiding ongoing medical management.

If a trained health care professional (doctor, nurse, ambulance personnel) is present, then request this person to undertake a physical examination. It is accepted that in the circumstances surrounding the event that this may be limited in extent.

1B Immediate post flight/event

Medical history of event

Detailed occupational history of fume event – including time, severity and duration of the fume event and frequency, duration and intensity of previous fume event exposures – see environmental record, 1A	
Record total flying hours – actual/estimated – Full-time or % part-time	
Medical record of event – see 1A (any additional information)	

Clinical examination

General appearance<br>(e.g. breathlessness, pallor, agitation)	
Record<br>● Blood pressure<br>● Heart rate	

• Respiratory rate<br>• Oxygen saturation (before and after oxygen administration, flow rate and method of administration (e.g. mask). Monitor if <95%<br>• Auscultation heart and lungs<br>• General neurological status: (conscious state, balance, muscle weakness, numbness, pupils, muscle reflexes, check for tingling of limbs, muscle cramps, tremor)<br>• Mental and cognitive state - clear thinking, problem solving:… (May use Mini-Mental State Examination- 'MMSE' - (Orientation for time and place; attention and calculation; memory and processing speed).	
Other findings	

General investigations

It is expected that persons who have experienced ill health during or following a fume event will consult their general practitioner or another medical practitioner, such as the company doctor, accident and emergency department doctor or a medical specialist. It is expected that most doctors will be grateful for guidance regarding appropriate clinical investigations. These tests to complement the clinical examination will give more detailed insight into the symptoms being experienced.

Special investigations should be undertaken as early as possible but should be between two to four hours and three days following exposure to complement the above clinical examination. Some pathological abnormalities will resolve fairly quickly and for this reason blood tests should be undertaken as early as possible after exposure. As an example, blood carbon monoxide levels are reduced by half every two to four hours. Other tests have a two- to three-day window. Many of these tests are routinely available but some may need referral to specialised medical units.

Routinely available:

- Full blood examination: (Hb, WCC and differential count)
- Acute phase reactants (e.g. C-reactive protein, ESR, fibrinogen)
- Routine biochemistry (U&E/Cr, LFTs, LDH)
- Muscle enzymes (e.g. Troponin, CKMM and CKMB, aldolase)
- Blood for cholinesterase (both red cell (AChE) and plasma (BChE) estimations are needed along with later baseline) [a] – see Table 1
- Others, as clinically indicated
- Carboxyhemoglobin **(**HbCO) Record time since exposure and/or time of last cigarette – maximum two to four hours post flight – short half-life
- Methaemoglobin – maximum two to four hours post flight (short half-life)
- Simplified cognitive assessment – Basic quick (5 min) testing of processing speed using the Symbol Digit Modalities Test (SDMT) (oral and written) and/or digit span forwards and backwards. Follow up with early referral for more detailed neuropsychological testing if required – See section 3C below.
- Other tests as clinically indicated

Non-routinely available: (require specialist laboratories, storage, shipping and analysis – more costly) – Table 1 and Table 2

- Bloods for neuro target esterase (NTE) [a,b]
- Urine for organophosphates [a,b]
- Blood for volatile organic hydrocarbons (VOCs) [b]

Footnotes:

a. Level of OP exposure may not be high enough to show enzyme inhibition or TCP urinary metabolites. Lack of inhibition or metabolites does not indicate that OP exposure did not take place.

b. Testing is not routinely available and requires specialist laboratories.

Note: Note that a formal chain of custody should be considered for all samples.

Table 1. Acetylcholinesterase (AChE) / butyrylcholinesterase (BChE) / neuro target esterase (NTE) sampling.

Method	Enzyme	Half-life (days)	Sample 1 (time after incident)	Sample 2 (Baseline) (time after incident)	Sample details
**Enzyme assays**	AChE - Red blood cell (RBC)	33	Preferably 4 - 48 hours	2-3 months [a]	Standard protocol**
	BChE - Plasma	12	Preferably 4 – 24 hours	1-2 months [a]	Standard protocol**
	NTE * (lymphocytic)	5-7		2-3 months. [a]	Standard protocol* – Only fresh blood can be analysed.

* Not routinely available – (require specialist laboratories, storage, shipping and analysis – more costly).

[a.] Second sample to be undertaken as a baseline. AChE recovers to normal level after around two to three months, while BChE recovers after around one to two months. If symptoms alleviate before this time, undertake baseline sample before returning to work or when away from further exposures. It is preferable to undertake a baseline before starting flying employment.

** Baseline AChE and BChE values for OP exposures have been generally determined for agricultural exposures, but not for aircraft fume event exposures. Note that there is a wide variation between individual baseline levels and therefore it is the 30-70% inhibition below the individual baseline that is the important reference. Each laboratory will use differing reference levels, which do not take into account the individual variation, which is the most important factor as outlined above.

Note: Refer to main medical protocol report (supplement) for Mass spec AChE and BChE benefits and analysis, which is being undertaken as part of a research project at the University of Washington.

Table 2. Non routinely available*/ – Blood analysis for VOCs & urine analysis for OPs**

		Sample 1	Further samples	Sample details
**Blood**	VOCs	As soon as possible after event	If possible 6 and 12 hours later and 1 month later [a]	5 ml normal EDTA tube. (2 ml transferred asap to coated headspace tubes) [b]
**Urine**	OPs	As soon as possible after event	If possible, 6 and 12 hours and 1 month later [a]	20 ml

Footnotes:

* Requires contact with specialist laboratories in advance of testing, storage and shipping – more costly.

** Refer to the main medical protocol report (supplement) for further methodology and limitations.

[a]. Last sample to be undertaken one month later as a baseline. VOCs mainly recover to normal levels within hours and OPs probably within two or three days. If symptoms alleviate before this time, undertake baseline sample with at least one week away from flying environment.

b. 2 ml blood samples have to be transferred as soon as possible to coated headspace tubes for gas chromatography–mass spectrometry (GC–MS) analysis. Contact a specialized laboratory certified for the required analysis**.**

Note: Level of OP exposure may not be high enough to show TCP urinary metabolites- see Section 2 in main medical protocol report (supplement).

Blood analysis for VOCs:

Human biomonitoring (HBM) can be undertaken for VOCs, including: aldehydes, aliphatics, aromatics, ketones, alcohols and organics such as n-heptane, n-hexane, benzene, toluene, formaldehyde, acetaldehyde, n-pentane, n-octane and carboxylic acids (valeric acid/pentanoic acid, heptanoic acid, octanoic acid) etc.

Urine analysis for OPs:

HBM can be undertaken for the following OPs: triaryl, trialkyl, triaryl-alkyl organophosphates (OPs). The analysis group for the OPs may include: Tricresyl phosphate (TCP)[a]; Trixylyl phosphate (TXP)[a]; Tributyl phosphate (TBP)[b]; Triphenyl phosphate (TPP)[c], with other potential OPs being: dibutyl phenyl phosphate (DBPP)[b]; tri-isobutyl phosphate (TiBP)[b]; 2,6-di-tert-butyl-p-cresol (BHT)[b] and, isopropylated phenyl, phosphate (3:1) (TIPP/PIP (3:1))[c] as well as mixed esters.

Note:

a. Utilised in selected oils.
b. Utilised in selected hydraulic fluids.
c. Utilised in selected oils and hydraulic fluids.

OP metabolites:

Dicresyl phosphates are known to be urinary metabolites of TCP in animal experiments. Three metabolites of tricresyl phosphate isomers—*oo* -, *mm* -, *pp* include dicresyl phosphate (D*o*CP, D*m*CP, D*p*CP). Dialkyl phosphate metabolites of tributyl phosphate (DBP), and triphenyl phosphate (DPP) may also be assessed. See Section 2 of main protocol for further details.

***Note:** Level of OP exposure may not be high enough to show TCP urinary metabolites- see Section 2 in main medical protocol report (supplement).*

Autoantibodies against neuronal and glial proteins in blood biomarker testing:

- Not currently available.
- See emerging issues (C), appendix 8 of main medical protocol report (supplement).

1C: Late/subsequent:

The medical approach is similar to that for earlier presentations.

- Medical history of the event – see Section 1B.
- Clinical examination – see Section 1B.
- Referral for specialist consultation should be considered as appropriate. Refer sections 2- 7 below.

2. Lung/heart

2A: Immediate post flight /event

- Respiratory and heart rate.
- Auscultation of lung and heart.
- Blood pressure (if measurement and trained personnel available).
- Oxygen saturation SpO_2, (record inspired oxygen concentration, e.g., air, 2L/min by mask etc.).
- Monitor oxygen saturation if <95%.
- Spirometry.
- ECG, if indicated e.g., presence of cardiac irregularity.
- Blood tests as clinically indicated.

Specialist tests within two weeks as required

Respiratory function testing within two weeks

- Detailed lung function tests (spirometry, DLCO and FeNO and/or DLNO (if available)).
- Check oxygen saturation SpO2.

Consider

- Arterial blood gas analysis breathing room air at rest- undertake earlier if clinical need- see below in notes.
- Expired nitric oxide (FeNO) if available.
- Exercise testing with oxygen saturation or blood gas analysis.
- Exhaled gas analysis (ergospirometry, if available).
- Blood tests (troponin, if indicated e.g., presence of cardiac irregularity).
- ECG – if required.

Notes :

- Arterial blood gas analysis is a semi-invasive procedure that perhaps could be avoided in subjects that do not complain about respiratory symptoms or who show an oxygen saturation value >96% at rest and/or during a 6 Minute Walk Test.
- Spirometry is a simple test measuring basic lung volumes that can be easily performed everywhere as it does not require sophisticated equipment. It should be performed promptly because symptoms of respiratory tract irritation may be transitory.
- Measurement of DLCO and/or DLNO are procedures that detect injuries of lung diffusion but are not available in all medical settings. However, these tests should be arranged in persons with respiratory symptoms, such as cough, shortness of breath, oxygen saturation <96% and in all those with abnormal spirometric values. The same approach should be applied for exercise testing or ergospirometry.
- In case the afore-mentioned investigations are not available or in the presence of serious respiratory abnormalities, the patient should be referred immediately to a respiratory specialist or hospital.

2B: Late / subsequent – if symptoms persist over weeks or months

If significant respiratory/cardiac symptoms are present or continue, consider referral to a respiratory specialist/pulmonologist and or cardiologist for an opinion and consideration of the following:

- Repeat routine lung function tests (spirometry, diffusing capacity).
- Static lung volumes.
- Percutaneous oxygen saturation or arterial blood gas analysis, as indicated.

- Appropriate radiology, e.g. chest x-ray, high resolution lung scan (HRCT chest).
- Respiratory orientated exercise test or screen with 6-minute walk test.
- Respiratory muscle strength testing.
- Bronchial provocation (methacholine, mannitol or other agent) testing.
- Blood tests as clinically indicated.
- Specific cardiac function tests as appropriate.
- Exercise testing with oxygen saturation or blood gas analysis.

3. Neurological

3A: Immediate post flight/event

- Full general medical assessment.
- Detailed neurological assessment and examination.
- Objective assessment of vestibular function.
- MRI brain scan.
- Consider referral to neurologist, severe neurological symptoms and signs.

3B: Late subsequent – if symptoms persist over weeks or months

- Full general medical assessment.
- Detailed neurological assessment and examination.
- Objective assessment of vestibular function.
- MRI – Refer to methodology in **Reneman et al. (2016)**
- PET/SPECT – Refer to methodology in **Heuser et al. (2005)**
- EMG/ENG: polyneuropathy.
- Skin biopsy/IENF (intraepidermal nerve fibers) – Small fiber neuropathy – There is an international guideline on how to perform this diagnostic. See **Lauria et al. (2010).**

Reneman L, et al. *Cognitive Impairment and Associated Loss in Brain White Microstructure in Aircrew Members Exposed to Engine Oil Fumes. Brain Imaging Behav. 2016; DOI: 10.1007/s11682-015-9395-3.*

Heuser G, et al. *Clinical Evaluation of Flight Attendants After Exposure to Fumes in Cabin Air. J Occup Health and Safety - Aust NZ. 2005; 21(5): 455-9.*

Lauria G, et al. *European Federation of Neurological Societies / Peripheral Nerve Society Guideline on the Use of Skin Biopsy in the Diagnosis of Small Fiber Neuropathy. Report of a Joint Task Force of the European Federation of*

Neurological Societies and the Peripheral Nerve Society. European Journal of Neurology. 2010; DOI: 10.1111/j.1468-1331.2010.03023.x.

3C: Neurocognitive

Neurocognitive adverse effects are reported to include: processing speed (written and oral); attention and concentration; reaction time to stimuli; sequential reaction time; complex problem solving; short and long term visual and verbal memory; cognitive flexibility / capacity to change direction.

Immediate/post flight/event

Neurocognitive testing:

- Coding test from WAIS.
- Symbol Digit Modalities Test (written and oral versions) – see Section 1B.
- CALCAP – Simple and choice reaction time tests.

Note: All should be able to be administered by medical personnel.

Late subsequent – if symptoms persist over weeks or months

Formal neurocognitive testing:

- Test for processing speed such as the Coding Test (WAIS), Symbol Digit Modalities Test (written and oral), Symbol Search (WAIS) and Trail Making test A.
- Tests of New Learning, such as the Austin Maze and the Rey Auditory Verbal Learning Test (RAVLT).
- Memory tests, such as those in the Wechsler Memory Scale, including visual and verbal memory.
- Problem Solving tests, such as the Category Test. The Wisconsin Card Sorting Test and the Stroop Test.
- Fine motor tests, such as the Reitan Finger Tapping Test of manual speed, the Grooved Pegboard Test of manual dexterity and the Dynamometer Grip Strength Test.
- In case of sleep disturbances consider full polysomnography.
- Boston Naming Test of language skills.

Tests available may vary in different countries, however most of the tests listed are universal and come primarily from the United States.

As an example, tests utilised in Germany may include: FAKT-II; RSAT; KVT-C; IGD; CompACT-Vi; Regensburger Wortflüssigkeits-Test.

4. Other areas of investigation:

To be considered based on clinical need including:

1) **Irritants:**

Management – Immediate post-flight / Late/subsequent:

- Avoid ongoing exposure to irritants.
- Manage symptoms as appropriate to the organ system involved.

2) **Sensitisation:**

Management – Immediate post-flight / Late/subsequent:

- Avoid ongoing exposure to irritants.
- Manage symptoms as appropriate to the organ system involved.
- Consider referral to organ system specialist.

3) **Skin:**

Management – Immediate post-flight / Late/subsequent:

- Avoid ongoing exposure to irritants.
- Consider standard dermatological treatment.
- Manage symptoms as appropriate to the organ system involved.
- Consider referral to dermatologist if recurrent.

4) **Gastrointestinal:**

Management – Immediate post-flight / Late/subsequent:

- Investigations as clinically indicated.

- Consider referral to gastroenterologist.

5) **Other**: Chronic fatigue; chemical sensitivity; reproductive effects; malignancy; susceptibility to infections; sleep disturbances, visual effects and joint aches and pains.

Refer main medical protocol report (supplement) for further guidance.

6) **Diagnostic coding:**

Many insurance companies and others require a specific formally recognised diagnosis listed in published diagnostic coding systems, such as the International Classification of Diseases (ICD). These can be accessed at https://www.icd10data.com. Some useful ICD10 diagnostic codings are listed below:

- J68.8: Other respiratory conditions due to chemicals, gases, fumes and vapours.
- J68.9: Unspecified respiratory condition due to chemicals, gases, fumes and vapours.
- T52.8: Toxic effects of other organic solvents.
- T52.9 Toxic effects of unspecified organic solvent.
- T65.9: Toxic effect of unspecified substance.
- G64: Other disorders of peripheral nervous system.
- G62.2: Polyneuropathy due to other toxic agents.
- G62.9: Polyneuropathy, unspecified.
- F06.7: Mild cognitive disorder.

- G92: Toxic encephalopathy (If the toxic agent is to be indicated an additional code number should be used such as those listed above)

- J98.8 Other specified respiratory disorders.

- J98.9: Respiratory disorder, unspecified – (respiratory disease (chronic) NOS)

7) Other areas to consider include: (*see* main medical protocol report (supplement) for further information)

  - Exposure to ultrafine particles.
  - Low level repeat exposure to mixtures.
  - Autoantibodies against neuronal and glial proteins in blood biomarker testing.
  - Chronic low-level exposure to OPs.
  - Acute and chronic exposures.
  - Dose.
  - Endocrine disruptors.
  - Causal connection.

Appendix 5: Symptoms during / after a fume event

**Symptom (circle specific one where necessary)**	**❑NO**<br>**❑YES**	**Body part/ Details**	**❑MINOR (-)**<br>**❑AVERAGE (✓)**<br>**❑MAJOR (+)**	**Start/ Duration**
Headache / pressure in head				
Partial / full / impaired consciousness				
Tingling (where)				
Numbness (where)				
Tremors / shaking				
Balance problems / erratic movement				
Visual problems				
Sweating / loss of temperature control / flushing / pallor				
Altered taste / metallic taste				
Incoordination				
Dizziness / light-headedness				
Discomfort / disorientation / confusion				
Drowsiness / lethargy				
Concentration / memory / cognitive problems				

Trouble writing / speaking				
Diarrhoea				
Cramping				
Vomiting				
Nausea				
Breathing problems				
Cough / respiratory irritation				
Chest discomfort / pains / tightness				
Increased heart rate				
Palpitations				
Eye irritation				
Throat irritation / hoarseness				
Nose irritation				
Sinus problems				
Joint / muscle pain / twitching				
Weakness / performance decrement				
Fatigue				
Skin reaction / blisters				
Other				

Appendix 6: Blood and urine testing for VOCs and OPs

Table 1. OPs, VOCs – Blood/urine – Non routinely available

		**Sample 1**	**Further samples**	**Sample details**
**Blood**	VOCs	As soon as possible after event	If possible 6 and 12 hours later and 1 month later [a]	5 ml normal EDTA. (2 ml transferred asap to coated headspace tubes) [b]
**Urine**	OPs	As soon as possible after event	If possible, 6 and 12 hours and 1 month later [a]	20ml

Footnotes

a. Last sample to be undertaken 1 month later as a baseline. VOCs mainly recover to normal levels within hours and OPs probably within two or three days. If symptoms alleviate before this time, undertake baseline sample with at least one week away from the flying environment.
b. 2ml blood samples have to be transferred ASAP to coated headspace tubes for Gas Chromatography-Mass spectrometry (GCMS) analysis. Contact a specialized laboratory certified for the required analysis.

Note: Level of OP exposure may not be high enough to show TCP urinary metabolites- see Section 2.

Appendix 7: Supplementary table: Human biomonitoring

Advantage of human biomonitoring (HBM)

It is important not only to identify routes and pathways of exposure, but also to determine that exposure and absorption have occurred. Although there are many ways to quantify the airborne load of dangerous substances in the workplace and the general environment, the measurements of toxins in the air only indicate a potential risk from exposure by inhalation and simply serve to document them. In contrast, human biomonitoring affords the rational possibility of measuring the polluting dose in an organism, providing a relevant estimate of the contamination regardless of the route of exposure.

Subsumed under the term human biomonitoring is an established, standardised analytical procedure for the diagnostic investigation of biological materials with the analytical measurement of biomarkers in specified units of body products (i.e. blood, urine). Biomarkers can be any substances or processes that are measurable and indicate exposure or susceptibility or that predict the incidence or outcome of disease. The combination of exposure biomonitoring and effect biomonitoring can support the clinical diagnosis, strengthening the association between symptoms and the specific causative exposure.

Limitations

One of the important limiting factors with respect to biomonitoring is the lack of knowledge about several chemicals and/or the cumulative effects of various chemicals. For the majority of chemicals, their molecular patho-mechanisms in humans, modes of transport to an affected organ, association with the development of diseases, and modes of elimination from the body are all largely unknown.

There are currently no realistic half-life times for most of the relevant substances. If there are any half-lives, they are derived from animal experiments or a single chemical industry incident. In both cases the half-lives are derived from exposures to extremely high, mostly single substance (in other cases one or two high doses and extrapolate them to lower realistic concentrations, assuming that there is always a linear dose-response). It does not refer to a realistic situation we have with fume events, which is a co-exposure of several substances, some of them in high, some in lower concentrations (this may also differ in a given situation). Note, many chemicals are metabolised through different pathways with different concentrations and also are metabolised

differently with co-exposure to other substances (this means that the balance between the metabolites and the parent substance changes).

Human biomonitoring is only of practical value if it employs analytical methods that have been validated with respect to specificity, limit of detection, reliability, and routine use. The biomonitoring analyses must include state-of-the-art internal and external quality assessment, at the same time keeping the pre-analytical and analytical factors as low as possible.

To address the important question of whether the detected low level of a chemical marker is a health hazard, several tests should be combined with clinical occupational medicine anamnesis data and, if possible, with the evaluation of personal susceptibility (acquired and genetic).

Appendix 8: Procedures for Determination of Autoantibodies to Neural Proteins.

Data source: Abou-Donia et al. (45, 299).

The following text and methodology provide additional information for the autoantibodies against neuronal and glial proteins in blood biomarker testing. It was hypothesised that exposure of cabin personnel to chemically contaminated cabin air causes neuronal cell death and the release of their neuronal and glial proteins into circulation across the leaky blood brain barrier. The references cited provide the most detailed methodology and explanation (45, 199, 299-302).

1. Plasma procedures

All sites followed the same protocol; for venipuncture, blood handling, plasma separation, aliquoting and storage at -20°C (preferably -80°C).

2. Western blot assay

Western blot analysis was used to determine autoantibodies against specific proteins in the plasma sample. The assays allowed the determination of the autoantibodies and associated isoforms of the antigen. Each plasma sample was analysed in triplicate (45). All proteins were loaded at 10 ng/lane except for IgG which was loaded as 100 ng/lane. The proteins were denatured and electrophoresed on SDS-PAGE (gradient 4% to 20%) purchased from Invitrogen (Carlsbad, CA). One gel was used for each serum sample. The proteins were transferred to polyvinylidene fluoride (PVDF) membranes (Amersham). Nonspecific binding sites were blocked with Tris-buffered Saline-Tween (TBST) (40 mM Tris [pH 7.6], 300 mM NaCl and 0.1% Tween 20) containing 5% fat-free milk powder for 1 hour at 22 °C. Membranes were incubated with serum samples at 1: 100 dilutions in TBST with 3% non-fat milk powder overnight at 4 °C. After five washes in TBST, the membranes were incubated with a 1:2000 dilution of goat anti-human IgG conjugated to horseradish peroxidase (Amersham). Membranes were developed by enhanced chemiluminescence using the manufacturer's protocol (Amersham) and a Typhoon 8600 variable model recorder. The signal intensity was quantified using Bio-Rad image analysis software (Hercules, California).

3. Specificity of plasma autoantibodies

Previously, the specificity of the plasma and serum autoantibody was checked by performing a peptide/antigen competition assay, in which the serum and plasma were spiked with the target protein or peptide (45, 199). The serum from random healthy controls was mixed with or without tau, MAP2, or MBP. The protein mix was centrifuged at 15,000 rpm to deplete any immune complexes. The supernatants were then carefully removed and used in a western blot. Specificities of autoantibodies against all tested proteins were confirmed in a follow-up study (299-302).

4. Statistics

The pooled data are presented as mean ± SD for continuous variables and the number and percent of participants in each category for categorical variables. Subjects' demographic values were compared to the control groups using Students t-test continuous and chi-square for categorical variables. Mean values of autoantibodies of the subjects were compared using analysis of covariance (ANCOVA). A two-sided p value <0.05 was accepted as statistically significant for all analyses and analyses were not adjusted for multiple comparisons. Analyses were carried out using SAS version 9.4

5. Calculations

Optical density measurement for subjects and controls was divided by the concentration of serum IgG; this value for each subject was normalised to controls and expressed as change from healthy controls. Therefore, the results are expressed as mean triplicate assay values of arbitrary optical density units normalized to IgG optical density as compared to healthy controls.

6. CNS Autoantibody Neurodegeneration Index (NDI)

This index determines the overall neurodegenerative condition of an individual based on the level of autoantibodies in the plasma. It is calculated by adding all of the values of autoantibodies for each neural protein, and then dividing the sum by the number of the autoantibodies used. Finally, the value is multiplied by ten to produce an easy NDI as previously reported (301, 302).

7. Brief methodology

Sera to be isolated using a western blot to estimate immunoglobulin-G (IgG) specific autoantibodies measured against cytoskeletal proteins associated with GFAP, S100B, MBP, MAG, neurofilament, tubulin, MAP-2, TAU, CamK2 and alpha synuclein.

- Samples may be collected shortly after a fume event or at a subsequent date (any time post fume event) as chronic exposure is assumed.
- Collect a total of 7 ml of blood in a test tube with a red top. Let the sample sit for 20 minutes at room temperature, and then centrifuge the sample to separate the serum. Transfer the serum to another test tube with a red top. The test tube containing serum must be kept frozen (preferably at -80 deg C, but at least -20 deg C), so should be packed in an insulated box containing dry ice.
- Contact specialist laboratory for storage and shipping instructions.

Appendix 9A: Hazard classifications associated with substances in oils based on EU CLP inventory database.

Data source:(72-74).

Substance	TCP	Meta / para TCP isomers	ToCP	TXP	PAN	Alkylated diphenyl amines	Base stock – Carboxylic acids	TIPP*
Substance used in product	Oils	Oils	Oils	Oils	Oils	Oils	Oils	Oils
Level in product	1-5%	<3%		0.1-1%	1-<2.5 %	1-5%	95%	0-2.5%
Damage to fertility or unborn child	X			X**				
Damage to organs - prolonged or repeated exposure	X			X	X			
Damage to organs - single exposure	X		X**					
Allergic skin reaction / sensitisation	X		X	X	X			X
Allergy or asthma symptoms or breathing					X			

difficulties if inhaled								
Respiratory irritation					X		X	
Harmful if swallowed / in contact with skin	X	X**						
Harmful if swallowed								
Fatal if inhaled			X					
Serious eye irritation	X			X	X	X		
Skin irritation					X			
Skin burns / eye damage							X	
May cause genetic defects			X					

*Also known as PIP (3:1) and also used in some newer hydraulic fluids.

**Harmonized classification.

<u>Appendix 9B: Hazard classifications associated with substances in the hydraulic (HF) and Deicing (DI) fluids based on EU CLP based inventory database.</u>

Data source (72-74)

Substance	TBP	TPP	Dibutyl phenyl phosp-hate	2,6-di-tert-butyl-*p*-cresol	TIPP/ PIP (3:1)	Other	Ethyl-ene Glycol	Propy-lene glycol
Substance used in product	HF	HF	HF	HF	HF	HF	DI	DI
Level in product	20-80%	1-2.5%	20-30%	1-5%	10-<20%	<10%	92%	55%
Damage to fertility or unborn child				X	X		X	
Damage to organs - prolonged or repeated exposure	X				X		X	
Damage to organs - single exposure							X	
Allergic skin reaction / sensitisation				X	X	X		X
Allergy or asthma symptoms or breathing difficulties if inhaled						X		
Harmful if swallowed	X*						X*	
Harmful in contact with skin			X	X				
Harmful if inhaled			X	X				
Toxic if inhaled				X				
Toxic in contact with skin				X				

Serious eye irritation	X	X	X	X		X	X	X
Serious eye damage						X		
Skin irritation	X*		X	X		X	X	X
Respiratory irritation			X					X
May cause drowsiness / dizziness							X	X
Suspected of causing cancer	X*			X				
Genetic defects				X			X	

* Harmonized classification.

REFERENCES

1. Kitzes G. Cabin Air Contamination Problems in Jet Aircraft. Aviation Medicine. 1956; 27(1): 53-8. PMCID: 13286221. https://perma.cc/QN4C-CB6R.
2. Gutkowski G, Page R, Peterson M. B-52 Decontamination Program. Seattle: Boeing Airplane Company; 1953. Report No. D-14766-2. https://ca-times.brightspotcdn.com/5e/30eaf151104b9787942f39d79f87e9/ex.%201%20-%20Decontamination%20Program.pdf. Accessed 1 December 2022.
3. Reddall HA. Elimination of Engine Bleed Air Contamination. SAE Technical Paper 550185. Warrendale, PA, USA: Society of Automotive Engineers; 1955. http://dx.doi.org/10.4271/550185. Accessed 1 December 2022.
4. Loomis T, Krop S. Cabin Air Contamination in RB-57A Aircraft. Maryland, USA: Chemical Corps Medical Laboratory, US Army; 1955. Medical Laboratories Special Report (MLSR) No. 61. https://perma.cc/D6G5-LJG6. Accessed 1 December 2022.
5. AMA. Aviation Toxicology: An Introduction to the Subject and a Handbook of Data. US Aero Medical Association, Committee Aviation Toxicoloy, editors. New York: Blakiston Company; 1953. DOI: 10.1001/jama.1953.02940290066035.
6. Davidson T, Cooley, T. and Way, J. Air Force Experience with Synthetic Gas Turbine Lubricants. SAE Technical Paper 550080. Warrendale, PA, USA: Society of Automotive Engineers; 1955. https://doi.org/10.4271/550080. Accessed 1 December 2022.
7. Gaume J. Analytical Considerations Concerned With Cephalagia On The Dc-10. Long Beach: Aviation Medicine and Safety Research Science Research; McDonnell Douglas Corporation; 1973. https://perma.cc/6HLD-3N4J. Accessed 1 December 2022.
8. Tashkin DP, Coulson AH, Simmons MS, Spivey GH. Respiratory Symptoms Of Flight Attendants During High-Altitude Flight: Possible Relation To Cabin Ozone Exposure. International archives of occupational and environmental health. 1983; 52(2): 117-37. DOI: 10.1007/BF00405416.
9. Rayman R, McNaughton G. Smoke and Fumes in the Cockpit. Aviation, Space and Environmental Medicine. 1983; 54(8): 738-40. PMCID: 6626083. https://pubmed.ncbi.nlm.nih.gov/6626083/.
10. Cone J. Interim Report No. 1 & 2 - Association Of Professional Flight Attendants Health Survey. San Francisco General Hospital Medical Center. Occupational Health Clinic. September 1983, June 1984. San Francisco: San Francisco General Hospital Medical Centre 1984. https://perma.cc/CDG6-HXE5.
11. Winder C, Balouet JC, Aerotoxic Sydrome: Adverse Health Effects Following Exposure to Jet Oil Mist During Commercial Flights. ‘editor’ Eddington I. Towards a Safe and Civil Society: Proceedings of the International Congress on Occupational Health Conference; 2000 4-6 September 2000; Brisbane, Australia: ICOH. https://perma.cc/44EC-EMR4.
12. PCA. Air Safety And Cabin Air Quality in the BAe 146 Aircraft - Report by the Senate Rural and Regional Affairs and Transport References Committee - Final Report. Canberra, Australia: Parliament of the Commonwealth of Australia; October, 2000. http://www.aph.gov.au/binaries/senate/committee/rrat_ctte/completed_inquiries/1999-02/bae/report/report.pdf Accessed 1 December 2022.
13. Montgomery M, Wier GT, Zieve F, Anders MW. Human Intoxication Following Inhalation Exposure to Synthetic Jet Lubricating Oil. Clin Toxicol. 1977; 11(4): 423-6. DOI: 10.3109/15563657708988205.
14. van Netten C. Air Quality and Health Effects Associated with the Operation of BAe 146-200 Aircraft. Applied Occupational and Environmental Hygiene. 1998; 13(10): 733-9. DOI: 10.1080/1047322X.1998.10390150.

15. Winder C, Balouet JC. Aircrew Exposure to Chemicals in Aircraft: Symptoms of Irritation and Toxicity. J of Occup Health and Safety – Austr New Zealand. 2001; 17: 471-83. https://perma.cc/539Z-K2EL.
16. Winder C, Fonteyn P, Balouet JC. Aerotoxic Syndrome: A Descriptive Epidemiological Survey of Aircrew Exposed to In-Cabin Airborne Contaminants. J Occup Health Safety - Aust NZ. 2002; 18(4): 321-38. https://perma.cc/7C6P-V7PL.
17. Cox L, Michaelis S. A Survey of Health Symptoms in BAe 146 Aircrew. J of Occup Health and Safety - Aust NZ. 2002; 18(4): 305-12.
18. Michaelis S. A Survey of Health Symptoms in BALPA Boeing 757 Pilots. Journal of Occupational Health and Safety -Aust and NZ. 2003; 19(3): 253-61.
19. Somers M. Aircrew Exposed to Fumes on the Bae 146: An Assessment of Symptoms. J Occup Health and Safety - Austr New Zealand. 2005; 21(5): 440-9.
20. Abou-Donia MB. Organophosphate Ester Induced Chronic Neurotoxicity,. Journal of Occupational Health Safety-Aust NZ. 2005; 21(5): 408-32.
21. Michaelis S. The 2009 UK BAe 146 /146 RJ Health Survey. Section 4.3.4. In PhD Thesis: Health and Flight Safety Implications From Exposure to Contaminated Air in Aircraft. [PhD]. University of New South Wales: Australia; 2010. http://handle.unsw.edu.au/1959.4/50342. Accessed 1 December 2022.
22. Harper A. A Survey of Health Effects in Aircrew Exposed to Airborne Contaminants. J Occup Health & Safety - Austr and New Zealand. 2005; 21(5): 433-9. https://perma.cc/R4RA-QQNT.
23. Murawski J. Case Study: Analysis of Reported Contaminated Air Events at One Major US Airline in 2009–10. Paper ID: AIAA-2011-5089. Proc 41st Intl Conf on Environ Sys, Am Inst Aeronaut Astronaut, 17–21 July, 2011; Portland. AIAA; 2011. p. 1-11. DOI: 10.2514/6.2011-5089.
24. McNeely E, Gale S, Tager I, Kincl L, Bradley J, Coull B, et al. The Self-Reported Health of US Flight Attendants Compared to the General Population. Environmental Health. 2014; 13(13): 1-11. DOI: 10.1186/1476-069X-13-13.
25. McNeely E, Mordukhovich I, Tideman S, Gale S, Coull B. Estimating the Health Consequences of Flight Attendant Work: Comparing Flight Attendant Health to the General Population in a Cross-Sectional Study. BMC Public Health. 2018; 18(346): 1-11. DOI: 10.1186/s12889-018-5221-3.
26. Passon D. The International Crew Health Survey. J of Biological Physics and Chemistry. 2011; 11: 201-7. DOI: 10.4024/26PA11A.jbpc.11.04.
27. Winder C, Michaelis S. Crew Effects from Toxic Exposures on Aircraft. In: Hocking M, editor. Air Quality in Airplane Cabins and Similar Enclosed Spaces. 4 H. Berlin/Heidelberg, Germany: Springer-Verlag; 2005. p. 223-42. DOI: 10.1007/b107246. ISBN: 978-3-540-25019-7.
28. Michaelis S, Burdon J, Howard CV. Aerotoxic Syndrome : A New Occupational Disease ? Public Health Panorama (WHO). 2017; 3: 198-211. https://apps.who.int/iris/handle/10665/325308.
29. Harrison R, Murawski J, Mcneely E, Guerriero J, Milton D. Exposure to Aircraft Bleed Air Contaminants Among Airline Workers - A Guide for Health Care Providors. San Francisco, CA, USA: Occupational Health Research Consortium in Aviation (OHRCA); 2009. http://www.ohrca.org/medical-protocols-for-crews-exposed-to-engine-oil-fumes-on-aircraft/. Accessed 1 December 2022.
30. Michaelis S. Health and Flight Safety Implications From Exposure to Contaminated Air in Aircraft [PhD thesis]. Sydney, Australia: University of New South Wales; 2010. http://handle.unsw.edu.au/1959.4/50342. Accessed 1 December 2022.
31. Hecker S, Kincl L, McNeely E, van Netten C, Harrison R, Murawski J, et al. Cabin Air Quality Incidents Project Report. Occupational Health Research Consortium in Aviation (OHRCA) and Airliner Cabin Environment Research (ACER); 2014. http://www.ohrca.org/wp-content/uploads/2014/08/finalreport.pdf. Accessed 1 December 2022.

32. Coxon L. Neuropsychological Assessment of a Group of BAe 146 Aircraft Crew Members Exposed to Jet Engine Oil Emissions. J of Occup Health and Safety - Aust and New Zealand. 2002; 18(4): 313-9.
33. Coxon L. Delayed Cognitive Impairment and Pilot Incapacitation Following Contaminated Air Inhalation. Journal of Biological Physics and Chemistry. 2014; 14: 107-10. DOI: 10.4024/13CO14M.jbpc.14.04.
34. Mackenzie Ross S, Harper A, Burdon J. Ill Health Following Exposure to Contaminated Aircraft Air : Psychosomatic Disorder or Neurological Injury? J Occup Health Safety — Aust NZ. 2006; 22(6): 521-8.
35. Mackenzie Ross S. Cognitive Function Following Exposure to Contaminated Air on Commercial Aircraft: A Case Series of 27 Pilots Seen for Clinical Purposes. J Nutritional and Environ Med. 2008; 17(2): 111-26. DOI: 10.1080/13590840802240067.
36. Mackenzie Ross SJ, Harrison V, Madeley L, Davis K, Abraham-Smith K, Hughes T, et al. Cognitive Function Following Reported Exposure to Contaminated Air on Commercial Aircraft: Methodological Considerations for Future Researchers. J Biological Physics and Chemistry. 2011; 11: 180-91. DOI: 10.4024/30MA11A.jbpc.11.04.
37. Reneman L, Schagen SB, Mulder M, Mutsaerts HJ, Hageman G, de Ruiter MB. Cognitive Impairment and Associated Loss in Brain White Microstructure in Aircrew Members Exposed to Engine Oil Fumes. Brain Imaging Behav. 2016; 10(2): 437-44. DOI: 10.1007/s11682-015-9395-3.
38. Heuser G, Auillera O, Heuser S, Gordon R. Clinical Evaluation of Flight Attendants After Exposure to Fumes in Cabin Air. J Occup Health and Safety - Aust NZ. 2005; 21(5): 455-9. https://perma.cc/QJ8Q-XV6V.
39. Pinkerton L, Hein J, Grajewski B, Kamel F. Mortality from Neurodegenerative Diseases in a Cohort of US Flight Attendants. American Journal of Industrial Medicine; 2016. p. 532-7. DOI: 10.1002/ajim.22608.
40. Burdon J, Glanville A. Lung Injury Following Hydrocarbon Inhalation in BAe 146 Aircrew. Journal of Occupational Health and Safety Aust NZ. 2005; 21: 450-4. DOI: 10.1007/b107244.
41. Roig J, Domingo C, Burdon J, Michaelis S. Irritant-Induced Asthma Caused by Aerotoxic Syndrome. Lung. 2021; 199: 165-70. DOI: 10.1007/s00408-021-00431-z.
42. Heutelbeck A. Progress Report: Diagnostics of Health Disorders and Biomonitoring in Aircraft Crewmembers After "Fume Events" - Preliminary Results After Analysing Patient Files. International Aircraft Cabin Air Conference 2017; 19-20 September 2017; Imperial College, London, England. J Health Pollution; 2019. p. S38-S42. DOI: 10.5696/2156-9614-9.24.191201.
43. Burdon J. Lung Injury Following Hydrocarbon Inhalation in Aircrew. Journal of Biological Physics and Chemistry. 2012; 12: 98-102.
44. Liyasova M, Li B, Schopfer LM, Nachon F, Masson P, Furlong CE, et al. Exposure to Tri-o-cresyl Phosphate Detected in Jet Airplane Passengers. Toxicology and Applied Pharmacology. 2011; 256(3): 337-47. DOI: 10.1016/j.taap.2011.06.016.
45. Abou-Donia MB, Abou-Donia MM, ElMasry Eea. Autoantibodies to Nervous System-Specific Proteins are Elevated in Sera of Flight Crew Members: Biomarkers for Nervous System Injury. J Toxicol Environ Health A. 2013; 76(6): 363-80. DOI: 10.1080/15287394.2013.765369.
46. Heutelbeck ARR, Bornemann C, Lange M, Seeckts A, Müller MM. Acetylcholinesterase and Neuropathy Target Esterase Activities in 11 Cases of Symptomatic Flight Crew Members After Fume Events. Journal of Toxicology and Environmental Health, Part A. 2016; 79(22-23): 1050-6. DOI: 10.1080/15287394.2016.1219561.
47. Hageman G, Pal TM, Nihom J, MackenzieRoss SJ, van den Berg M. Three Patients with Probable Aerotoxic Syndrome. Clinical Toxicology (Phila). 2019; 58(2): 139-42. DOI: 10.1080/15563650.2019.1616092.
48. Schindler BK, Weiss T, Schütze A, Koslitz S, Broding HC, Bünger J, et al. Occupational Exposure of Air Crews to Tricresyl Phosphate Isomers and Organophosphate Flame Retardants After Fume Events. Archives of Toxicology. 2013; 87(4): 645-8. DOI: 10.1007/s00204-012-0978-0.

49. Heutelbeck A, Baur X, Belpoggi F, Budnik LT, Burdon J, Gee D, et al., On the Need for a Standardized Human Biomonitoring Protocol for In-Flight Incidents (Called "Fume Events"). 'editor' 2nd International DiMoPEx Conference on "Pollution in Living and Working Environments and Health," DiMoPEx Working Groups Meeting; 2018. 30-31 October 2017; Cesare Maltoni Cancer Research Center, Ramazzini Institute, Bologna, Italy: Journal of Health and Pollution. DOI: https://doi.org/10.5696/2156-9614.8.17.1.
50. Hageman G, Pal TM, Nihom J, Ross SJM. Aerotoxic Syndrome: Discussion of Possible Diagnostic Criteria. Clin Toxicol. 2019; 58(5): 414-6. DOI: 10.1080/15563650.2019.1649419.
51. IATA. IATA Guidance for Airline Health and Safety Staff on the Medical Response to Cabin Air Quality Events: Smoke, Fumes/Odours. Montreal, Canada: International Air Transport Association; 2015. https://www.iata.org/contentassets/ccbdc54681c24574bebf2db2b18197a5/guidance-medical-response-cabin-air-events.pdf. Accessed 1 December 2022.
52. CAA. Information for Health Professionals on Aircraft Fume Events. Civil Aviation Authority - UK Department for Transport/Division 2017. https://www.caa.co.uk/passengers/before-you-fly/am-i-fit-to-fly/guidance-for-health-professionals/aircraft-fume-events/. Accessed 1 December 2022.
53. EPAAQ. Expert Panel on Aircraft Air Quality (EPAAQ). Contamination of Aircraft Cabin Air by Bleed Air – A Review of the Evidence. Canberra, Australia: Civil Aviation Safety Authority; 2012. https://skybrary.aero/sites/default/files/bookshelf/3595.pdf. Accessed 1 December 2022.
54. NRC. The Airliner Cabin Environment and the Health of Passengers and Crew. US National Reseach Council, editor. Washington, DC: National Academy Press; 2002. DOI: https://doi.org/10.17226/10238. ISBN: 0-309-08289-7.
55. ICAO. Guidelines on Education, Training and Reporting Practices Related to Fume Events. Montréal, Canada: International Civil Aviation Organization; 2015.
56. ANSI/ASHRAE. Standard 161: Air Quality within Commercial Aircraft. Atlanta. GA, USA. American Society of Heating, Refrigerating, and Air Conditioning Engineers. 2007.
57. Howard CV, Johnson DW, Morton J, Michaelis S, Supplee D, Burdon J. Is a Cumulative Exposure to a Background Aerosol of Nanoparticles Part of the Causal Mechanism of Aerotoxic Syndrome ? Nanomedicine and Nanoscience Research. 2018; JNAN-139: 1-8. DOI: 10.29011/JNAN-139. 100039.
58. Lixian LI, Di Matteo M, Hendrick P. Experimental Study to Verify Oil Loss Through the Vent Line of the Aero-Engine Lubrication System. 9TH European Conference For Aeronautics And Space Sciences (EUCASS); 27 June- 1 July; Lille, France. 2022. DOI: 10.13009/EUCASS2022-4391.
59. Howard CV, Michaelis S, Watterson A. The Aetiology of 'Aerotoxic Syndrome' - A Toxico-Pathological Viewpoint. Open Acc J of Toxicol. 2017; 1(5): 555575. DOI: 10.19080/OAJT.2017.01.555575.
60. ASHRAE. Guideline 28: Air Quality within Commercial Aircraft. Atlanta, GA, USA: American Society of Heating, Refrigerating, and Air Conditioning Engineers; 2012.
61. CAA. CAA Paper 2004/04: Cabin Air Quality. Gatwick Airport, W. Sussex, England: Civil Aviation Authority, UK Department for Transport 2004. https://publicapps.caa.co.uk/docs/33/CAPAP2004_04.PDF. Accessed 1 December 2022.
62. ExxonMobil. Jet Oil Chemistry and Composition- Considerations for Odor Formation and Risk Assessment. ExxonMobil; 2018.
63. AIHA. Odour Thresholds for Chemicals with Established Occupational Health Standards. Fairfax, VA, USA: American Industrial Hygiene Association; 1989. ISBN: 13: 9780932627346.
64. AAIU. Air Accident Investigation Unit Ireland: AAIU Report No: 2016-013: Boeing 737-8AS, EI-EFB: 18 September 2014. Dublin: Air Accident Investigation Unit (Ireland) 2016. http://www.aaiu.ie/sites/default/files/report-attachments/REPORT%202016-013.pdf. Accessed 1 December 2022.

65. CAA. Health Information for Passengers/Cabin Air Quality. Gatwick: Civil Aviation Authority, Civil Aviation Authority - UK Department for Transport/Division 2017. https://www.caa.co.uk/Passengers/Before-you-fly/Am-I-fit-to-fly/Health-information-for-passengers/Cabin-air-quality/. Accessed 1 December 2022.
66. Schuchardt S, Bitsch A, Koch W, Rosenberger W. EASA Research Project: CAQ Preliminary Cabin Air Quality Measurement Campaign. Final Report EASA_REP_RESEA_2014_4. Cologne, Germany: European Aviation Safety Agency; 2017. https://www.easa.europa.eu/document-library/research-reports/easarepresea20144. Accessed 1 December 2022.
67. Holley J. Reducing Smoke and Burning Odor Events. Boeing Aero Quarterly. QTR-01.09. 2009:6-9. https://www.boeing.com/commercial/aeromagazine/articles/qtr_01_09/article_02_1.html. Accessed 1 December 2022.
68. Overfelt R, Jones B, Loo Sea. Rite-ACER-COE-2012-05. Sensors and Prognostics to Mitigate Bleed Air Contamination Events -2012 Progress Report. Auburn: Airliner Cabin Environment Research; 2012. Report No. RITE-ACER-CoE-2012-05. https://www.faa.gov/data_research/research/med_humanfacs/cer/media/SensorsPrognostics.pdf. Accessed 1 December 2022.
69. Michaelis S. Is it Time to Act? Journal of Biological Physics and Chemistry. 2014; 14(4): 133-5. DOI: 10.4024/19MI14M.jbpc.14.04.
70. Winder C. Hazardous Chemicals on Jet Aircraft: Jet Oils and Aerotoxic Syndrome. Curr Topics Toxicol. 2006; 3: 65-88. http://www.researchtrends.net/tia/abstract.asp?in=0&vn=3&tid=50&aid=2177&pub=2006&type=3.
71. Winder C, Balouet J-C. The Toxicity of Commercial Jet Oils. Environmental Research. 2002; 89(2): 146-64. DOI: 10.1006/enrs.2002.4346.
72. European Commission. Regulation (EC) No 1272/2008 of the European Parliament and of the Council of 16 December 2008 on Classification, Labelling and Packaging of Substances and Mixtures (CLP). (2009). https://eur-lex.europa.eu/legal-content/EN/LSU/?uri=celex%3A32008R1272.
73. ECHA. CLP Classification and Labelling Database. Helsinki: European Chemicals Agency; 2021. Available from: https://echa.europa.eu/information-on-chemicals/cl-inventory-database. Accessed 1 December 2022.
74. Michaelis S. REACH-CLP Review of Oils Hydraulic & Deicing Fluids: S Michaelis University of Stirling; 2019. https://perma.cc/MD9N-7FM9 Accessed 1 December 2022.
75. UN. Globally Harmonized System of Classification and Labelling of Chemicals (GHS) New York , Geneva: United Nations; 2017. https://unece.org/fileadmin/DAM/trans/danger/publi/ghs/ghs_rev07/English/ST_SG_AC10_30_Rev7e.pdf
www.unece.org/trans/danger/publi/ghs/implementation_e.html.
76. Mackerer C, Ladov E. Mobil Oil Submission (14a): Submission to the Inquiry Into Air Safety - BAe 146 Cabin Air Quality: Vol 3. Canberra, Australia: Parliament of the Commonwealth of Australia; 2000. https://perma.cc/BA88-8XAJ. Accessed 1 December 2022.
77. Mackerer CR, Barth ML, Krueger AJ, Roy TA. Comparison of Neurotoxic Effects and Potential Risks From Oral Administration or Ingestion of Tricresyl Phosphate and Jet Engine Oil Containing Tricresyl Phosphate. Journal of Toxicology and Environmental Health, Part A. 1999; 57(5): 293-328. DOI: 10.1080/009841099157638.
78. PCA. Mobil Oil Australia. Written submission No.13 by Julian Plummer - Inquiry into Air Safety and Cabin Air Quality in the BAe 146 Aircraft. Parliament of the Commonwealth of Australia; 1999 7 October, 1999. https://perma.cc/2D8U-R77V.

79. EASA. A-NPA-2009-10. Cabin Air Quality Onboard Large Aeroplanes. Cologne: European Aviation Safety Agency; 2009. https://www.easa.europa.eu/document-library/notices-of-proposed-amendments/npa-2009-10. Accessed 1 December 2022.
80. Houtzager M, Noort D, Joosen M, Bos J, Jongeneel R, van Kesteren P, et al. Characterisation of the Toxicity of Aviation Turbine Engine Oils After Pyrolysis (AVOIL). Cologne, Germany: European Aviation Safety Agency; 2017. https://www.easa.europa.eu/document-library/research-reports/easarepresea20152. Accessed 1 December 2022.
81. SAE. Air 4766/2- Airborne Chemicals In Aircraft Cabins. Warrendale: Society of Automotive Engineers; 2005. https://www.sae.org/standards/content/air4766/2/. Accessed 1 December 2022.
82. Jones B, Amiri S, Roth J, Hosni M. The Nature of Particulates in Aircraft Bleed Air Resulting from Oil Contamination. LV-17-C046. 2017 ASHRAE Winter Conference; 28 January - 1 February 2017; Las Vegas, NV, USA. American Society of Heating, Refrigerating, and Air Conditioning Engineers; 2017.
83. Crump D, Harrison P, Walton C. Aircraft Cabin Air Sampling Study; Part 1 and 2 of the Final Report. Cranfield, England: Institute of Environment and Health, Cranfield University; 2011. http://dspace.lib.cranfield.ac.uk/handle/1826/5305
http://dspace.lib.cranfield.ac.uk/handle/1826/5306. Accessed 1 December 2022.
84. Rosenberger W, Beckmann B, Wrbitzky R. Airborne Aldehydes in Cabin-Air of Commercial Aircraft: Measurement by HPLC With UV Absorbance Detection of 2,4-Dinitrophenylhydrazones. Journal of Chromatography B. 2016; 1019: 117-27. DOI: 10.1016/j.jchromb.2015.08.046.
85. Spengler J, Vallarino J, Mcneely E, Estephan H. In-Flight/Onboard Monitoring: ACER’s Component for ASHRAE, 1262, Part 2. Boston, MA, USA: Research in the Intermodal Transport Environment/Aircraft Cabin Environment Research; 2012. https://www.faa.gov/data_research/research/med_humanfacs/cer/media/In-FlightOnboardMonitoring.pdf. Accessed 1 December 2022.
86. Schuchardt S, Koch W, Rosenberger W. Cabin Air Quality – Quantitative Comparison of Volatile Air Contaminants at Different Flight Phases During 177 Commercial Flights. Building and Environment. 2019; 148: 498-507. DOI: 10.1016/j.buildenv.2018.11.028.
87. Chen R, Fang L, Liu J, Herbig B, Norrefeldt V, Mayer F, et al. Cabin Air Quality on Non-Smoking Commercial Flights: A Review of Published Data on Airborne Pollutants. Indoor Air. 2021; 31(4): 926-57. DOI: 10.1111/ina.12831.
88. Diamond M. Cabin Air Quality Monitoring – Organophosphates Sampling during Fume Events in Australia. In: Scholz D, editor. Aircraft Cabin Air International Conference 2021; 15-18 March Virtual. 2021. DOI: https://doi.org/10.5281/zenodo.5567739.
89. Fox R, Chase D, Kurlak P, Koerner M, Hagh B, Johnson R, et al., inventors; Honeywell International Inc., assignee. Human Factors Approach to Control Contaminant Concentrations in Aircraft Supply Air from Engine and APU Bleed Air Air and Ground Air Sources, and in Recirculated Air Being Delivered to Aircraft Cabins for the Optimization of User Experience and Energy Consumption. USA patent US9902499B2. 2018. https://worldwide.espacenet.com/patent/search?q=pn%3DUS9902499B2
90. Fox R. Assessing Aircraft Supply Air to Recommend Compounds for Timely Warning of Contamination [PhD]. San Diego, CA, USA: Northcentral University; 2012. https://www.proquest.com/docview/1021859974/2E9451C80D5A4304PQ/1. Accessed 1 December 2022.
91. Space D, Matthews K, Takacs J, Umino P, Salgar A, Roth J, et al. Experimental Determination of the Characteristics of Lubricating Oil Contamination in Bleed Air. Lv-17-C047. 2017 ASHRAE Winter Conference 28 Jan- Feb 1; Las Vegas. ASHRAE; 2017. DOI: xxxx.
92. ASHRAE. ASHRAE Research Project Report 1830-RP: Experimental Characterization of Aircraft Bleed Air Particulate Contamination. Atlanta, GA, USA; 2022.

93. FAA. Aircraft Air Quality and Bleed Air Contamination Detection. William J. Hughes Technical Center; 2022. DOT/FAA/TC-21/45. http://www.tc.faa.gov/its/worldpac/techrpt/tc21-45.pdf. Accessed 1 December 2022.
94. Fox R. Thermal Decomposition Studies of Oils and Fuel Approved for Use in the Honeywell ALF 502/507 Engine - Study Date Dec 2001 - Jan 2002. (Presented to the UK Committee on Toxicity in December 2006). Phoenix, Arizona; 2006 20 October 2006. https://perma.cc/56E3-82FD.
95. Marshman S. Analysis of the Thermal Degradation Products Synthetic Ester Gas Turbine Lubricant. Farnborough, England: UK Ministry of Defence, Defence Evaluation and Research Agency; 2001 29 June 2001. https://perma.cc/435H-R2RW.
96. Havermans J, Houtzager M, Jacobs P. Incident Response Monitoring Technologies for Aircraft Cabin Air Quality Technology Review, Analysis and Performance Testing. Final Report. TNO-060-UTP-2013-00069 /ASHRAE 1306-RP. 2013. Report No. TNO-060-UTP-2013-00069. http://resolver.tudelft.nl/uuid:99b807f8-6993-4ea1-aeb4-d2edf3f2edf9.
97. Mair S, Scherer C, Mayer F. Emissionsverhalten eines Flugzeugmotorenols bei Thermischer Belastung - (Emission Characteristics of an Aircraft Engine Oil Under Thermal Stress). Gefahrstoffe- Reinhaltung der Luft. 2015; 75(7/8): 295-302. https://publica.fraunhofer.de/handle/publica/242321
98. Ritchie GD, Still KR, Rossi J, Bekkedal MYV, Bobb AJ, Arfsten DP. Biological and Health Effects of Exposure to Kerosene-Based Jet Fuels and Performance Additives. Journal of Toxicology and Environmental Health - Part B: Critical Reviews. 2003; 6(4): 357-451. DOI: 10.1080/10937400306473.
99. Chaturvedi A. Aerospace Toxicology: An Overview. Oklahoma City, OK, USA: Civil Aeromedical Institute, US Federal Aviation Administration; 2009. Report No. DOT/FAA/AM-09/8. https://www.faa.gov/data_research/research/med_humanfacs/oamtechreports/2000s/media/200908.pdf. Accessed 1 December 2022.
100. Linke-Diesinger A. Systems of Commercial Turbofan Engines: An Introduction to Systems Functions: Springer, Berlin, Heidelberg; 2008. DOI: 10.1007/978-3-540-73619-6. ISBN: 978-3-540-73618-9.
101. ExxonMobil. Jet Engine Used Oil Evaluation. ExxonMobil; 2016. Available from: https://www.exxonmobil.com/en/aviation/~/media/files/global/us/aviation/Knowledge-Library/learningandresources_tech-topics_used-oil-evaluation. Accessed 1 December 2022.
102. FAA. FAA-H-8083-32. The Aviation Maintenance Handbook–Powerplant - Vol 2. Oklahoma City: Federal Aviation Administration; 2018. ISBN: 13: 9781490427638. https://www.faa.gov/regulations_policies/handbooks_manuals/aviation/media/FAA-H-8083-32-AMT-Powerplant-Vol-2.pdf
103. ACGIH. TLVs And BEIs - Threshold Limit Values for Chemical Substances and Physical Agents. Cincinnati, Ohio, USA: American Conference of Governmental Industrial Hygienists; 2015. https://www.acgih.org/publications/digital-pubs/#2015tlv. Accessed 2 December 2022.
104. Barnes J, WHO. Toxicité Pour L'homme De Certains Pesticides. Genève: Organisation mondiale de la Santé.; 1954. https://apps.who.int/iris/handle/10665/38801. Accessed 2 December 2022.
105. Lenfant C, Sullivan K. Adaption to High Altitude. New England Journal of Medicine. 1971; 284(23): 1298-309. DOI: 10.1056/NEJM197106102842305.
106. Lockhart A, Saiag B. Altitude and the Human Pulmonary Circulation. Clinical Science. 1981; 60(6): 599-605. DOI: 10.1042/cs0600599.
107. Samuels M. The Effects of Flight and Altitude. Archives of Disease in Childhood. 2004; 89: 448-55. DOI: 10.1136/adc.2003.031708.
108. Weisel CP, Fiedler N, Weschler CJ, Ohman-strickland P, Mohan KR, Mcneil K, et al. Human Symptom Responses to Bioeffluents, Short-Chain Carbonyls/Acids and Long-Chain

Carbonyls in a Simulated Aircraft Cabin Environment. Indoor Air. 2017; 27(6): 1154-67. DOI: 10.1111/ina.12392.
109. de Ree H, van den Berg M, Brand T, Mulder GJ, Simons R, Veldhuijzen van Zanten B, et al. Health Risk Assessment of Exposure to Tricresyl Phosphates (TCPs) in Aircraft: A Commentary. Neurotoxicology. 2014; 45: 209-15. DOI: 10.1016/j.neuro.2014.08.011.
110. COT. Position Paper on Cabin Air. London, England: UK Committee Of Toxicity; 2013. https://cot.food.gov.uk/sites/default/files/cot/cotpospapcabin.pdf. Accessed 2 December 2022.
111. de Boer J, Antelo A, van der Veen I, Brandsma S, Lammertse N. Tricresyl Phosphate and the Aerotoxic Syndrome of Flight Crew Members - Current Gaps in Knowledge. Chemosphere. 2015; 119: S58-S61. DOI: 10.1016/j.chemosphere.2014.05.015.
112. Hausherr V, van Thriel C, Krug A, Leist M, Schöbel N. Impairment of Glutamate Signaling in Mouse Central Nervous System Neurons in Vitro by Tri-Ortho-Cresyl Phosphate at Noncytotoxic Concentrations. Toxicol Sci. 2014; 142(1): 274-84. DOI: 10.1093/toxsci/kfu174.
113. Baker PE, Cole TB, Cartwright M, Suzuki SM, Thummel KE, Lin YS, et al. Identifying Safer Anti-Wear Triaryl Phosphate Additives for Jet Engine Lubricants. Chemico-Biological Interactions. 2013; 203(1): 257-64. DOI: 10.1016/j.cbi.2012.10.005.
114. Duarte DJ, Rutten JMM, van den Berg M, Westerink RHS. In Vitro Neurotoxic Hazard Characterization of Different Tricresyl Phosphate (TCP) Isomers and Mixtures. NeuroToxicology. 2017; 59: 222-30. DOI: 10.1016/j.neuro.2016.02.001.
115. He R, Houtzager M, Jongeneel W, Westerink R, Cassee F. In Vitro Hazard Characterization of Simulated Aircraft Cabin Bleed-Air Contamination in Lung Models Using an Air-Liquid Interface (ALI) Exposure System. Environment International. 2021; 156: 106718. DOI: 10.1016/J.ENVINT.2021.106718.
116. European Commission. FACTS - Aircraft Air Quality Study. Brussels, Belgium: European Commission; 2017. Available from: https://www.facts.aero/index.php. Accessed 2 December 2022.
117. European Commission. FACTS: Fresh Aircraft. Deliverable 07 - Final Report: Research Study: Investigation of the Quality Level of the Air Inside the Cabin of Large Transport Aeroplanes and its Health Implication. 2021. https://www.facts.aero/images/Status/FACTS-D7-Final_Report.pdf.
118. EASA. EASA Workshop on Future Cabin Air Quality Research - 30-31 January 2020. Cologne: European Aviation safety Agency; 2020. 2 December 2022. https://www.easa.europa.eu/newsroom-and-events/events/easa-workshop-future-cabin-air-quality-research. Accessed 2 December 2022.
119. EASA. Cabin Air Quality Assessment of Long-Term Effects of Contaminants. Cologne, Germany: European Aviation Safety Agency; 2020. Available from: https://www.easa.europa.eu/research-projects/cabin-air-quality-assessment-long-term-effects-contaminants;https://www.item.fraunhofer.de/en/r-d-expertise/toxicology/cabin-air-quality.html. Accessed 2 December 2022.
120. US Congress. Public Law No: 115-254 (2018): H.R.302 - FAA Reauthorization Act of 2018: Sec. 326. Aircraft Air Quality., (2018). https://www.congress.gov/115/plaws/publ254/PLAW-115publ254.pdf. Accessed 2 December 2022.
121. BG Verkehr. Medizinisches Standardverfahren Nach Fume-Events (Standard medical procedure after fume events). Hamburg: BG Verkehr; 2014. Available from: https://www.bg-verkehr.de/redaktion/medien-und-downloads/informationen/branchen/luftfahrt/standard-verfahren-eng.pdf. Accessed 2 December, 2022.
122. CEN. Cabin Air Quality on Civil Aircraft - Chemical Compounds - Technical Report. Brussels: CEN CENELEC; 2022. PD CEN/TR 17904:2022. https://www.en-standard.eu/pd-cen-tr-17904-2022-cabin-air-quality-on-civil-aircraft-chemical-compounds/.
123. Feuillie V, Onboard Fume Events: Short Term Health Consequences in Aircrew -Air France Medical Department, Occupational Health Services. ‘editor’ EASA Workshop, Cologne, 30-31 January 2020; 2020; Cologne: European Aviation Safety Agency. https://www.easa.europa.eu/en/downloads/113142/en.

124. Michaelis S. Contaminated Aircraft Cabin Air. J Biological Physics and Chemistry. 2011; 11: 132-45. DOI: doi: 10.4024/24MI11A.jbpc.11.04.
125. Bachman G, Santos C, Weiland J, Hon S, Lopez G. Aerotoxic Syndrome: Fuming About Fumes While Flying the Friendly Skies. Clin Toxicol. 2017; 55(7): 773-4. DOI: 10.1080/15563650.2017.1348043.
126. O'Connor L, Boland M. A Series of Three Aircraft Emergency Public Health Alerts at an International Airport Over a Six Week Period due to Suspected Contaminated Cabin Air. Journal of Environmental Science and Public Health. 2020; 4(3): 296-303. DOI: 10.26502/jesph.96120101.
127. Abeyratne R. Forensic Aspects of the Aerotoxic Syndrome. Med Law. 2002; 21(1): 179-99. PMCID: 12017442.
128. Cherry N, Mackness M, Durrington P, Povey A, Dippnall M, Smith T, et al. Paraoxonase (PON1) Polymorphisms in Farmers Attributing Ill Health to Sheep Dip. Lancet. 2002; 359(9308): 763-4. DOI: 10.1016/S0140-6736(02)07847-9.
129. Burdon J. Respiratory Symptoms and Lung Injury After Inhaling Fumes on Aircraft: Toxic Fumes or Hyperventilation? Journal of Biological Physics and Chemistry. 2014; 14(4): 103-6. DOI: 10.4024/21BU14A.jbpc.14.04.
130. Worek F, Schilha M, Neumaier K, Aurbek N, Wille T, Thiermann H, et al. On-site analysis of acetylcholinesterase and butyrylcholinesterase activity with the ChE check mobile test kit—Determination of reference values and their relevance for diagnosis of exposure to organophosphorus compounds. Toxicology Letters. 2016; 249: 22-8. DOI: https://doi.org/10.1016/j.toxlet.2016.03.007. https://www.sciencedirect.com/science/article/pii/S0378427416300431.
131. Ruhnau P, Müller M, Hallier E, Lewalter J. Ein Validiertes Photometrisches Verfahren Für Die Bestimmung Der Neuropathy Target Esterase Aktivität In Humanen Lymphozyten. A Validated Photometric Assay for the Neuropathy Target Esterase Activity in Human Lymphocytes. Umweltmedizin in Forschung und Praxis. 2001; 6: 101-3.
132. ICSC. International Programme On Chemical Safety - N-Phenyl-1-Naphthylamine. Geneva: WHO; 2016. Available from: https://inchem.org/documents/icsc/icsc/eics1113.htm. Accessed 5 December 2022.
133. ExxonMobil. Material Safety Data Sheet: Mobil Jet Oil II. Spring Tx: Exxon Mobil Corporation; 2017. https://perma.cc/MF33-2CDD.
134. Nigg HN, Knaak JB. Blood Cholinesterases as Human Biomarkers of Organophosphorus Pesticide Exposure. Reviews of Environmental Contamination and Toxicology. 2000; 163: 29-111. DOI: 10.1007/978-1-4757-6429-1_2.
135. Aldridge WN. Tricresyl Phosphates and Cholinesterase. Biochem J. 1954; 56(2): 185-9. DOI: 10.1042/bj0560185.
136. Furlong C, Rettie A, Zelter A, McDonald M, MacCoss M, Marsillach J, et al., Have You Been Exposed to Aircraft Engine Oil? Biomarkers Of Exposure. 'editor' International Cabin Air Conference; 2021 15-18 March 2021; Virtual. DOI: 10.5281/zenodo.4730424.
137. Furlong CE. Exposure to Triaryl Phosphates: Metabolism and Biomarkers of Exposure. Journal of Biological Physics and Chemistry. 2011; 11: 1-11. DOI: 10.4024/28FU11A.jbpc.11.04.
138. Marsilliach J, Richter RJ, Kim JH, Stevens RC, MacCoss MJ, Tomazela D, et al. Biomarkers of Organophosphorus Exposures on Humans. NeuroToxicology. 2011; 32(5): 656-60. DOI: 10.1016/j.neuro.2011.06.005.
139. Mock DM, Lankford GL, Widness JA, Burmeister LF, Kahn D, Strauss RG. Measurement of Red Cell Survival Using Biotin-Labeled Red Cells: Validation Against 51cr-Labeled Red Cells. Transfusion. 1999; 39(2): 156-62. DOI: 10.1046/j.1537-2995.1999.39299154729.x.
140. Lepage L, Schiele F, Gueguen R, Siest G. Total Cholinesterase in Plasma: Biological Variations and Reference Limits. Clinical Chemistry. 1985; 31(4): 546-50. DOI: 10.1093/clinchem/31.4.546.
141. Carletti E, Colletier J-P, Schopfer LM, Santoni G, Masson P, Lockridge O, et al. Inhibition Pathways of the Potent Organophosphate CBDP With Cholinesterases Revealed by X-Ray

Crystallographic Snapshots and Mass Spectrometry. Chemical Research in Toxicology. 2013; 26(2): 280-9. DOI: 10.1021/tx3004505.
142. Lewalter J. Acetylcholinesterase Inhibitors. BAT Value Documentation. 1985; Vol 2. DOI: 10.1002/3527600418.bb0astrinhe0002. 10.1002/3527600418.bb0astrinhe0002.
143. Johnson D, Carter MD, Crow BS, Isenberg SL, Graham LA, Erol HA, et al. Quantitation of Ortho-Cresyl Phosphate Adducts to Butyrylcholinesterase in Human Serum by Immunomagnetic-UHPLC-MS/MS. Journal of Mass Spectrometry. 2015; 50(4): 683-92. DOI: 10.1002/jms.3576.
144. ACGIH. TLV Triorthocresyl Phosphate 78-30-8 - Chemical Substances Documentation Cincinnati: American Conference of Governmental Industrial Hygienists; 2016. https://www.acgih.org/. Accessed 2 December 2022.
145. Casida JE. Specificity of Substituted Phenyl Phosphorus Compounds for Esterase Inhibition in Mice. Biochemical pharmacology. 1961; 5(4): 332-42. DOI: 10.1016/0006-2952(61)90024-7.
146. van Netten C, Leung V. Hydraulic Fluids and Jet Engine Oil: Pyrolysis and Aircraft Air Quality. Archives of Environmental Health. 2001; 56(2): 181-6. DOI: 10.1080/00039890109604071.
147. Budnik LT, Kloth S, Baur X, Preisser AM, Schwarzenbach H. Circulating Mitochondrial DNA as Biomarker Linking Environmental Chemical Exposure to Early Preclinical Lesions Elevation of MtDNA in Human Serum After Exposure to Carcinogenic Halo-alkane-based Pesticides. PLOS ONE. 2013; 8(5): e64413. DOI: 10.1371/journal.pone.0064413.
148. Kloth S, Baur X, Göen T, Budnik LT. Accidental Exposure to Gas Emissions from Transit Goods Treated for Pest Control. Environmental Health. 2014; 13(1): 1-9. DOI: 10.1186/1476-069X-13-110.
149. Morgan MK, Sheldon LS, Jones PA, Croghan CW, Chuang JC, Wilson NK. The Reliability of Using Urinary Biomarkers to Estimate Children's Exposures to Chlorpyrifos and Diazinon. Journal of Exposure Science and Environmental Epidemiology. 2011; 21(3): 280-90. DOI: 10.1038/jes.2010.11.
150. Abou-Donia MB, Nomeir AA, Bower JH, Makkawy HA. Absorption, Distribution, Excretion and Metabolism of a Single Oral Dose of [14C]Tri-o-cresyl phosphate (ToCP) in the Male Rat. Toxicology. 1990; 65(1-2): 61-74. DOI: 10.1016/0300-483X(90)90079-V.
151. Somkuti SG, Abou-Donia MB. Disposition, Elimination, And Metabolism of Tri-o-cresyl phosphate Following Daily Oral Administration in Fischer 344 Male Rats. Archives of Toxicology. 1990; 64(7): 572-9. DOI: 10.1007/BF01971837.
152. Henschler D. Die Trikresylphosphatvergiftung. Experimentelle Klärung von Problemen der Ätiologie und Pathogenese (Tricresyl Phosphate Poisoning. Experimental Clarification of Problems of Etiology and Pathogenesis). Klinische Wochenscrifte. 1958; 36(14): 663-74. DOI: 10.1007/BF01488746 ; https://perma.cc/A465-LDSR.
153. Kurebayashi H, Tanaka A, Yamaha T. Metabolism and Disposition of the Flame Retardant Plasticizer, Tri-p-Cresyl Phosphate, in the Rat. Toxicology and Applied Pharmacology. 1985; 77(3): 395-404. DOI: 10.1016/0041-008X(85)90179-6.
154. Schindler B. Erarbeitung Und Anwendung Einer Analytischen Methode Zur Bestimmung Der Metabolite Von Flammschutzmitteln Auf Der Basis Von Phosphorsäuretriestern In Menschlichen KörperflüSsigkeiten [PhD]: Universität Erlangen-Nürnberg; 2009. https://d-nb.info/995579547/34 Accessed 2 December 2022.
155. Schindler BK, Förster K, Angerer J. Determination of Human Urinary Organophosphate Flame Retardant Metabolites by Solid-Phase Extraction and Gas Chromatography-Tandem Mass Spectrometry. Journal of Chromatography B: Analytical Technologies in the Biomedical and Life Sciences. 2009; 877(4): 375-81. DOI: 10.1016/j.jchromb.2008.12.030.
156. Erdely A, Hulderman T, Salmen R, Liston A, Zeidler-Erdely PC, Schwegler-Berry D, et al. Cross-Talk Between Lung and Systemic Circulation During Carbon Nanotube Respiratory Exposure. Potential Biomarkers. Nano Letters. 2009; 9(1): 36-43. DOI: 10.1021/nl801828z.

157. Kobayashi T, Tsuyoshi I. Diesel Exhaust Particulates Induce Nasal Mucosal Hyperresponsiveness to Inhaled Histamine Aerosol. Toxicological Sciences. 1995; 27(2): 195-202. DOI: 10.1093/toxsci/27.2.195.
158. Lam C-W, James JT, McCluskey R, Hunter RL. Pulmonary Toxicity of Single-Wall Carbon Nanotubes in Mice 7 and 90 Days After Intratracheal Instillation. Toxicological Sciences. 2004; 77(1): 126-34. DOI: 10.1093/toxsci/kfg243.
159. Muller J, Huaux F, Moreau N, Misson P, Heilier JF, Delos M, et al. Respiratory Toxicity of Multi-Wall Carbon Nanotubes. Toxicology and Applied Pharmacology. 2005; 207(3): 221-31. DOI: 10.1016/j.taap.2005.01.008.
160. Shvedova AA, Kisin ER, Mercer R, Murray AR, Johnson VJ, Potapovich AI, et al. Unusual Inflammatory and Fibrogenic Pulmonary Responses to Single-Walled Carbon Nanotubes in Mice. Am J Physiol Lung Cell Mol Physiol. 2005; 289: L698-L708. DOI: 10.1152/ajplung.00084.2005.
161. Meldrum K, Guo C, Marczylo EL, Gant TW, Smith R, Leonard MO. Mechanistic Insight into the Impact of Nanomaterials on Asthma and Allergic Airway Disease. Particle and Fibre Toxicology. 2017; 14(1): 45. DOI: 10.1186/s12989-017-0228-y.
162. AAIB. Report No. U1884. Investigation Report Concerning the Serious Incident to Aircraft Avro 146-RJ100, HB-IXN. Payerne: Swiss Transportation Safety Investigation Board; 2006. https://www.sust.admin.ch/inhalte/AV-berichte/1884_e.pdf.
163. Eastman. Eastman (Tm) Turbo Oil 2380: Safety Data Sheet. Kingsport, TN: Eastman Chemical Company; 2016. https://perma.cc/63PT-74BZ.
164. Carvalho R, Arukwe A, Ait-Aissa Sea. Mixtures of Chemical Pollutants at European Legislation Safety Concentrations: How Safe Are They? Toxicological Sciences. 2014; 141(1): 218-33. DOI: 10.1093/toxsci/kfu118.
165. IGHRC. The Interdepartmental Group on Health Risks From Chemicals. Chemical Mixtures : A Framework for Assessing Risks to Human Health. Cranfield: IEH, Cranfield University; 2009. http://www.iehconsulting.co.uk/IEH_Consulting/IEHCPubs/IGHRC/cr14.pdf. Accessed 2 December 2022.
166. European Commission. European Commission: Something From Nothing? Ensuring the Safety of Chemical Mixtures. Joint Research Centre: European Commission; 2018. https://data.europa.eu/doi/10.2760/618648. Accessed 2 december 2022.
167. Newman KL, Newman LS. Occupational Causes of Sarcoidosis. Current Opinion in Allergy and Clinical Immunology. 2012; 12(2): 145-50. DOI: 10.1097/ACI.0b013e3283515173.
168. Guzman D, Broderick B, Sotolongo A, Osinubi O, Helmer D. A Case of Sarcoidosis in a Veteran With Military-Related Environmental and Occupational Exposure. Chest. 2017; 152(4): A830. DOI: 10.1016/j.chest.2017.08.862.
169. New South Wales Court of Appeal: East West Airlines Limited V Turner [2010] NSWCA 53 - 1 April 2010(2010). http://www.austlii.edu.au/au/cases/nsw/NSWCA/2010/53.html. Accessed 2 December 2022.
170. High Court of Australia: East West Airlines Ltd V Turner [2010] HCATrans 238 (3 September 2010) & Original Decision: Turner V Eastwest Airlines Limited [2009] NSWDDT , 5 May 2009(2010). http://www.austlii.edu.au/au/other/HCATrans/2010/238.html http://www.austlii.edu.au/au/cases/nsw/NSWDDT/2009/10.html. Accessed 2 December 2022.
171. Zavorsky GS, Hsia CCW, Hughes JMB, Borland CDR, Guénard H, van der Lee I, et al. Standardisation and Application of the Single-Breath Determination of Nitric Oxide Uptake in the Lung. European Respiratory Journal. 2017; 49: 1600962. DOI: 10.1183/13993003.00962-2016.
172. Jensen R, Leyk M, Crapo R, Muchmore D, Berclaz PY. Quality Control of DL,CO Instruments in Global Clinical Trials. European respiratory Journal. 2009; 33(4): 828-34. DOI: 10.1183/09031936.00091208.
173. Roth A, Zellinger I, Arad M, Atsmon J. Organophosphates and the Heart. Chest. 1993; 103(2): 576-82. DOI: 10.1378/chest.103.2.576.
174. Sundaragiri S, Tandur S. Electrocardiographic Profile of Agrochemical Poisoning. International Journal of Contemporary Medical Research. 2016; 3(12): 3539-42.

175. Hung DZ, Yang HJ, Li YF, Lin CL, Chang SY, Sung FC, et al. The Long-Term Effects of Organophosphates Poisoning as a Risk Factor of CVDs: A Nationwide Population-Based Cohort Study. PLoS ONE. 2015; 10(9): e0137632. DOI: 10.1371/journal.pone.0137632.
176. Panikkath R, Nugent K, Perez-Verdia A. Atrial Fibrillation After Inhalational Lung Injury: A Troubling Complication of a Rare Problem. Case Reports in Medicine. 2012; 2012: 302057. DOI: 10.1155/2012/302057.
177. Aggarwal HK, Jain D, Goyal S, Dahiya S, Mittal A. Organophosphate Poisoning Presenting as Bradycardia. El Mednifico Journal. 2014; 2(2): 182. DOI: 10.18035/emj.v2i2.78.
178. Georgiadis N, Tsarouhas K, Tsitsimpikou C, Vardavas A, Rezaee R, Germanakis I, et al. Pesticides and Cardiotoxicity. Where Do We Stand? Toxicology and Applied Pharmacology. 2018; 353: 1-14. DOI: 10.1016/j.taap.2018.06.004.
179. Bar-Meir E, Schein O, Eisenkraft A, Rubinstein R, Grubstein A, Militianu A, et al. Guidelines for Treating Cardiac Manifestations of Organophosphates Poisoning with Special Emphasis on Long QT And Torsades De Pointes. Critical reviews in Toxicology. 2007; 37: 279-85. DOI: 10.1080/10408440601177855.
180. Anand S, Singh S, Nahar Saikia U, Bhalla A, Paul Sharma Y, Singh D. Cardiac Abnormalities In Acute Organophosphate Poisoning. Clin Toxicol. 2009; 47(3): 230-5. DOI: 10.1080/15563650902724813.
181. Abou-Donia MB, Goot FRWVD, Mulder MFA. Autoantibody Markers of Neural Degeneration are Associated with Post-Mortem Histopathological Alterations of a Neurologically Injured Pilot. Journal of Biological Physics and Chemistry. 2014; 14: 34-53. DOI: 10.4024/05AB14A.jbpc.14.03.
182. Old Square Chambers. Anna Roffey Appears in Inquest Concerning the Death of Matthew Bass. 2018. Available from: http://www.oldsquare.co.uk/news-and-media/articles/anna-roffey-appears-in-inquest-concerning-the-death-of-matthew-bass Accessed 2 December 2022.
183. Masuda T, Sawada Y, Tsutsumi K, Tsuchiya N, Yokouchi S, Ohwada T, et al. Toxic Myocarditis Caused by Acute Organophosphate Poisoning. Nihon Kyukyu Igakukai Zasshi. 1993; 4(4): 359-63. DOI: 10.3893/jjaam.4.359.
184. ExxonMobil. Material Safety Data Sheet: Mobil Jet Oil II. Antwerpen, Belgium; 2019. https://perma.cc/6YQ8-XL27.
185. Howard CV. Inappropriate Use of Risk Assessment in Addressing Health Hazards Posed by Civil Aircraft Cabin Air. Open Access Journal of Toxicology. 2020; 4(2): 555634. DOI: 10.19080/OAJT.2020.04.555634.
186. Terry A, Jr. Functional Consequences of Repeated Organophosphate Exposure: Potential Non-Cholinergic Mechanisms. Pharmacol Ther. 2012; 134(3): 355-65. DOI: doi:10.1016/j.pharmthera.2012.03.001.
187. Naughton SX, Terry AV. Neurotoxicity In Acute And Repeated Organophosphate Exposure. Toxicology. 2018; 408: 101-12. DOI: 10.1016/j.tox.2018.08.011.
188. Banks CN, Lein PJ. A Review of Experimental Evidence Linking Neurotoxic Organophosphorus Compounds and Inflammation. NeuroToxicology. 2012; 33(3): 575–84. DOI: 10.1016/j.neuro.2012.02.002.
189. Basheer IA, Hajmeer M. Artificial Neural Networks: Fundamentals, Computing, Design, And Application. Journal of Microbiological Methods. 2000; 43(1): 3-31. DOI: 10.1016/S0167-7012(00)00201-3.
190. Harris J, Blain P. Neurotoxicology: What the Neurologist Needs to Know. Journal of Neurology Neurosurgery Psychiatry. 2004; 75(Suppl 3): iii29 - iii34. DOI: 10.1136/jnnp.2004.046318.
191. Davies R, Ahmed G, Freer T. Chronic Exposure to Organophosphates: Background And Clinical Picture. Advances in Psychiatric Treatment. 2000; 6(3): 187-92. DOI: 10.1192/apt.6.3.187.
192. Abou-Donia MB. Organophosphorus Ester-Induced Chronic Neurotoxicity. Archives of Environmental Health. 2003; 58(8): 484-97. DOI: 10.3200/AEOH.58.8.484-497.

193. Abou-Donia M. Mechanisms of Organophosphorus Ester-Induced Delayed Neurotoxicity: Type-I and Type-II. Annual Review of Pharmacology and Toxicology. 1990; 30: 405-40. DOI: 10.1146/annurev.pa.30.040190.002201

194. Abou-Donia MB. Organophosphorus Ester-Induced Delayed Neurotoxicity. Annual Review of Pharmacology and Toxicology. 1981; 21: 511-48. DOI: 10.1146/annurev.pa.21.040181.002455.

195. Axelrad JC, Howard CV, McLean WG. Interactions Between Pesticides and Components of Pesticide Formulations in an In Vitro Neurotoxicity Test. Toxicology. 2002; 173(3): 259-68. DOI: 10.1016/S0300-483X(02)00036-7.

196. Axelrad JC, Howard CV, McLean WG. The Effects of Acute Pesticide Exposure on Neuroblastoma Cells Chronically Exposed to Diazinon. Toxicology. 2003; 185(1-2): 67-78. DOI: 10.1016/S0300-483X(02)00592-9.

197. Howard CV, Pathogenesis of Non-Specific Neurological Signs and Symptoms in Aircrew on Civil Aircraft. ‘editor’ Aviation Health Conference, London; 2018; London. https://perma.cc/7JWV-578V

198. Cherry N MM, Mackness B, et al. ‘Dippers' Flu’ And Its Relationship to PON1 Polymorphisms. Occupational and Environmental Medicine. 2011; 68(3): 211-7. DOI: doi: 10.1136/oem.2009.052126.

199. Abou-donia MB, Conboy LA, Kokkotou E, Jacobson E, Elmasry EM, Elkafrawy P, et al. Screening for Novel Central Nervous System Biomarkers in Veterans with Gulf War Illness. Neurotoxicology and Teratology. 2017; 61: 36-46. DOI: 10.1016/j.ntt.2017.03.002.

200. Costa LG. Organophosphorus Compounds at 80: Some Old and New Issues. Toxicological Sciences. 2018; 162(1): 24-35. DOI: 10.1093/toxsci/kfx266.

201. Merwin SJ, Obis T, Nunez Y, Re DB. Organophosphate Neurotoxicity to the Voluntary Motor System on the Trail of Environment-Caused Amyotrophic Lateral Sclerosis: The Known, the Misknown, And the Unknown. Archives of Toxicology. 2017; 91(8): 2939-52. DOI: 10.1007/s00204-016-1926-1.

202. Jokanović M. Neurotoxic Effects of Organophosphorus Pesticides and Possible Association with Neurodegenerative Diseases in Man: A Review. Toxicology. 2018; 410: 125-31. DOI: 10.1016/j.tox.2018.09.009.

203. Mostafalou S, Abdollahi M. The Link of Organophosphorus Pesticides with Neurodegenerative and Neurodevelopmental Diseases Based on Evidence and Mechanisms. Toxicology. 2018; 409: 44-52. DOI: 10.1016/j.tox.2018.07.014.

204. Nicholas J, Lackland D, Dosemeci M ea. Mortality Among US Commercial Pilots and Navigators. Journal of occupational and environmental medicine. 1998; 40(11): 980-5. DOI: 10.1097/00043764-199811000-00008.

205. Nicholas JS, Butler GC, Lackland DT, Tessier GS, Mohr J, Hoel DG. Health Among Commercial Airline Pilots. Aviation Space and Environmental Medicine. 2001; 72(9): 821-6. PMCID: 11565817.

206. Pilkington A, Buchanan D, Jamal GA, Gillham R, Hansen S, Kidd M, et al. An Epidemiological Study of the Relations Between Exposure to Organophosphate Pesticides and Indices of Chronic Peripheral Neuropathy and Neuropsychological Abnormalities in Sheep Farmers and Dippers. Occupational and Environmental Medicine. 2001; 58(11): 702-10. DOI: 10.1136/oem.58.11.702.

207. Sommer C, Geber C, Young P, Forst R, Birklein F, Schoser B. Polyneuropathies: Etiology, Diagnosis, and Treatment Options. Deutsches Ärzteblatt International | Dtsch Arztebl Int. 2018; 115: 83-90. DOI: 10.3238/arztebl.2018.083.

208. Devigili G, Tugnoli V, Penza P, Camozzi F, Lombardi R, Melli G, et al. The Diagnostic Criteria for Small Fibre Neuropathy: From Symptoms to Neuropathology. Brain. 2008; 131(7): 1912–25. DOI: 10.1093/brain/awn093.

209. Lauria G, Hsieh ST, Johansson O, Kennedy WR, Leger J, Mellgren S, et al. European Federation of Neurological Societies / Peripheral Nerve Society Guideline on the Use of Skin Biopsy in the Diagnosis of Small Fiber Neuropathy. Report of a Joint Task Force of the European Federation

of Neurological Societies and the Peripheral Nerve Society. European Journal of Neurology. 2010; 17: 903-12. DOI: 10.1111/j.1468-1331.2010.03023.x.
210. Devigili G, Rinaldo S, Lombardi R, Cazzato D, Marchi M, Salvi E, et al. Diagnostic Criteria for Small Fibre Neuropathy in Clinical Practice and Research. Brain. 2019; 142(12): 3728–36. DOI: 10.1093/brain/awz333.
211. Sibomana I, Good NA, Hellman PT, Rosado L, Mattie DR. Acute Dermal Toxicity Study of New, Used and Laboratory Aged Aircraft Engine Oils. Toxicology Reports. 2019; 6: 1246-52. DOI: 10.1016/j.toxrep.2019.11.010.
212. Boeing. Boeing MSDS No. 138541. Material Safety Data Sheet- Mil-PRF-23699 (lubricating Oil- Aircraft Turbine Engines- Synthetic Base). Rev 08/09/2007. Seattle: Boeing; 2007. https://perma.cc/KEV6-X6CD.
213. Ladov E. Letter From E Ladov to J Aveni Mobil Oil Corporation- Mobil Jet Oil II. January 24, 1983. New York: Mobil Oil Corporation; 1983. https://perma.cc/Z87P-3TXE.
214. NTP. NTP Chemical Repository (Radian Corporation, August 29, 1991) Tricresyl Phosphate. 1991. https://perma.cc/Z3NY-NTGA.
215. Chemtura. Material Safety Data Sheet- Durad 125 (Tricresyl Phosphate): MSDS Number: 00691. 2006. https://perma.cc/EPJ3-CN38.
216. Mobil. Mobil Jet Oil II Material Safety Data Bulletin -430207-00. Fairfax, Virginia: Mobil Oil Corp; 1999. https://perma.cc/U5GQ-UG99.
217. Eastman. Eastman Turbo Oil 2197 -Oil Can Label. 2018. https://perma.cc/4E8A-M725.
218. ExxonMobil. HYJET IV-A PLUS Hydraulic fluid Safety Data Sheet. Spring, TX: Exxon Mobil Corporation; 2021. https://perma.cc/CDY8-W6EE.
219. Eastman. Skydrol PE-5 - Hydraulic Fluid Safety Data Sheet. Eastman Chemical Company; 2019. https://perma.cc/V5TW-R4VH.
220. Dow. UCAR(TM) PG Aircraft Deicing Fluid Dilute "55/45". Midland, MI: DOW; 2009. https://perma.cc/YFG6-KRKH.
221. Shell. Aeroshell Turbine Oil 560: Safety Data Sheet. 2017. https://perma.cc/C2XG-DN3H.
222. IPCS. Environmental Health Criteria 110 -Tricresyl Phosphate -International programme on Chemical Safety. WHO; 1990. https://inchem.org/documents/ehc/ehc/ehc110.htm. Accessed 2 December 2022.
223. Eastman. Eastman Turbo Oil 2197 Safety Data Sheet. Kingsport, TN: Eastman Chemical Company; 2020. https://perma.cc/R3N2-5WMQ.
224. Esso. Memorandum On The Toxicity Of Synthetic Turbo Oils. Report No. MRD-17N-57; MR.12M.57. April 2, 1957. Esso - Medical Research Division: Esso Research and Engineering Company; 1957. https://perma.cc/VBM5-8VMT.
225. Hooper M. Multiple Chemical Sensitivity: Chapter 50. In: Puri B, Treasaden I, editors. Psychiatry: An Evidence Based Text: Hodder Arnold; 2009. p. 793-820. DOI: https://doi.org/10.1201/b13480 https://www.taylorfrancis.com/chapters/mono/10.1201/b13480-58/multiple-chemical-sensitivity-malcolm-hooper-bassant-puri-ian-treasaden?context=ubx&refId=ae471026-2705-4e48-9d73-2fc305541b21
226. Meggs WJ. The Role Of Neurogenic Inflammation In Chemical Sensitivity. Ecopsychology. 2017; 9(2): 83-9. DOI: 10.1089/eco.2016.0045.
227. Winder C. Mechanisms Of Multiple Chemical Sensitivity. Toxicology Letters. 2002; 128(1-3): 85-97. DOI: 10.1016/s0378-4274(01)00536-7
228. Masri S, Miller CS, Palmer RF, Ashford N. Toxicant-Induced Loss of Tolerance for Chemicals, Foods, and Drugs: Assessing Patterns of Exposure Behind a Global Phenomenon. Environmental Sciences Europe. 2021; 33(1). DOI: 10.1186/s12302-021-00504-z.
229. Miller C. The Quick Environmental Exposure and Sensitivity Inventory (QEESI). UT Health ,San Antonio 2021. Available from: https://tiltresearch.org/qeesi-2/. Accessed 2 December 2022.
230. Palmer RF, Jaen CR, Perales RB, Rincon R, Forster JN, Miller CS. Three Questions for Identifying Chemically Intolerant Individuals in Clinical and Epidemiological Populations: The

Brief Environmental Exposure and Sensitivity Inventory (BREESI). PLoS One. 2020; 15(9): e0238296. DOI: 10.1371/journal.pone.0238296.
231. ExxonMobil. Mobil Jet Oil II Safety Data Sheet. Spring, TX: Exxon Mobil Corporation; 2020. https://perma.cc/2J6P-DH4D.
232. Buja A, Mastrangelo G, Perissinotto E, Grigoletto F, Frigo AC, Rausa G, et al. Cancer Incidence Among Female Flight Attendants : A Meta-Analysis Of Published Data. 2006; 15(1): 98-105. DOI: 10.1089/jwh.2006.15.98.
233. Goodson WH, Lowe L, Carpenter DO, Gilbertson M, Ali AM, de Cerain Salsamendi AL, et al. Assessing the Carcinogenic Potential of Low-Dose Exposures to Chemical Mixtures in the Environment: The Challenge Ahead. Carcinogenesis. 2015; 36(1): S254-S96. DOI: 10.1093/carcin/bgv039.
234. Otto D. Andrew K Myers: Workers Compensation Board, State of Oregon. WCB Case No. 19-02648 - Opinion and Order. July 31, 2020. Oregon: Workers Compensation Board, State of Oregon; 2020. https://perma.cc/BZ6L-STQL
https://perma.cc/B3PL-R7A7.
235. PCA. Mobil Hansard: Oral Evidence - Inquiry Into Air Safety - BAe 146 Cabin Air Quality. Canberra, Australia; 1 February 2000, 2000. https://perma.cc/8NB7-JT4T.
236. Holgate S, Grigg J, al e. Every Breath We Take: The Lifelong Impact Of Air Pollution. Royal College of Physicians; 2016. https://www.rcplondon.ac.uk/projects/outputs/every-breath-we-take-lifelong-impact-air-pollution. Accessed 2 December 2022.
237. Künzli N, Kaiser R, Medina S, Studnicka M, Chanel O, Filliger P, et al. Public-Health Impact Of Outdoor And Traffic-Related Air Pollution: A European Assessment. Lancet: Lancet Publishing Group; 2000. p. 795-801. DOI: 10.1016/S0140-6736(00)02653-2.
238. Pope CA, Dockery DW. Health Effects of Fine Particulate Air Pollution: Lines That Connect. Journal of the Air & Waste Management Association (1995). 2006; 56(6): 709-42. DOI: 10.1080/10473289.2006.10464485.
239. Zhang J, Chen Z, Shan D, Wu Y, Zhao Y, Li C, et al. Adverse effects of exposure to fine particles and ultrafine particles in the environment on different organs of organisms. Journal of Environmental Sciences. 2022. DOI: 10.1016/j.jes.2022.08.013.
240. Zhang X, Chen X, Zhang X. The Impact of Exposure to Air Pollution on Cognitive Performance. Proceedings of the National Academy of Sciences. 2018; 115(37): 9193-7. DOI: 10.1073/PNAS.1809474115.
241. Lancet Editorial. Air Pollution And Brain Health: An Emerging Issue. The Lancet Neurology. 2018; 17(2): 103. DOI: 10.1016/S1474-4422(17)30462-3.
242. UNICEF. 17 Million Babies Under the Age of 1 Breathe Toxic Air, Majority Live in South Asia UNICEF; 2017 [6 December]. Available from: https://www.unicef.org/press-releases/babies-breathe-toxic-air-south-asia. Accessed 2 December 2022.
243. Carey IM, Anderson HR, Atkinson RW, Beevers SD, Cook DG, Strachan DP, et al. Are Noise and Air Pollution Related to the Incidence of Dementia? A Cohort Study in London, England. BMJ Open. 2018; 8(9): e022404. DOI: 10.1136/bmjopen-2018-022404.
244. Smith RB, Fecht D, Gulliver J, Beevers SD, Dajnak D, Blangiardo M, et al. Impact of London's Road Traffic Air and Noise Pollution on Birth Weight: Retrospective Population Based Cohort Study. BMJ (Online). 2017; 359: j5299. DOI: 10.1136/bmj.j5299.
245. Bonner JC. Nanoparticles as a Potential Cause of Pleural and Interstitial Lung Disease. Proceedings of the American Thoracic Society. 2010; 7(2): 138-41. DOI: 10.1513/pats.200907-061rm.
246. Mudway IS, Dundas I, Wood HE, Marlin N, Jamaludin JB, Bremner SA, et al. Impact of London's Low Emission Zone on Air Quality and Children's Respiratory Health: A Sequential Annual Cross-Sectional Study. The Lancet Public Health. 2019; 4: e28-e40. DOI: 10.1016/S2468-2667(18)30202-0.

247. Leiser CL, Hanson HA, Sawyer K, Steenblik J, Al-Dulaimi R, Madsen T, et al. Acute Effects of Air Pollutants on Spontaneous Pregnancy Loss: A Case-Crossover Study. Fertility and Sterility. 2019; 111(2): 341-7. DOI: 10.1016/j.fertnstert.2018.10.028.
248. Cesaroni G, Forastiere F, Stafoggia M, Andersen ZJ, Badaloni C, Beelen R, et al. Long Term Exposure to Ambient Air Pollution and Incidence of Acute Coronary Events: Prospective Cohort Study and Meta-Analysis in 11 European Cohorts From the Escape Project. BMJ (Online). 2014; 348: f7412. DOI: 10.1136/bmj.f7412.
249. Schraufnagel DE, Balmes JR, Cowl CT, De Matteis S, Jung S-H, Mortimer K, et al. Air Pollution And Noncommunicable Diseases: A Review by the Forum of International Respiratory Societies' Environmental Committee, Part 1: The Damaging Effects of Air Pollution Chest. 2019; 155(2): 409-16. DOI: 10.1016/j.chest.2018.10.042.
250. Schraufnagel DE, Balmes JR, Cowl CT, De Matteis S, Jung SH, Mortimer K, et al. Air Pollution and Noncommunicable Diseases: A Review by the Forum of International Respiratory Societies' Environmental Committee, Part 2: Air Pollution and Organ Systems. Chest. 2019; 155(2): 417-26. DOI: 10.1016/j.chest.2018.10.041.
251. Lavigne E, Lima I, Hatzopoulou M, Van Ryswyk K, van Donkelaar A, Martin RV, et al. Ambient ultrafine particle concentrations and incidence of childhood cancers. Environ Int. 2020; 145: 106135. DOI: 10.1016/j.envint.2020.106135.
252. Liao C-M, Chio C-P, Chen W-Y, Ju Y-R, Li W-H, Cheng Y-H, et al. Lung cancer risk in relation to traffic-related nano/ultrafine particle-bound PAHs exposure: A preliminary probabilistic assessment. Journal of Hazardous Materials. 2011; 190(1): 150-8. DOI: 10.1016/j.jhazmat.2011.03.017.
253. Ostrom QT, Bauchet L, Davis FG, Deltour I, Fisher JL, Langer CE, et al. The Epidemiology of Glioma in Adults: a “State of the Science" Review. Neuro Oncol. 2014; 16(7): 896-913. DOI: 10.1093/neuonc/nou087.
254. Wu AH, Fruin S, Larson TV, Tseng C-C, Wu J, Yang J, et al. Association between Airport-Related Ultrafine Particles and Risk of Malignant Brain Cancer: A Multiethnic Cohort Study. Cancer Research. 2021; 81(16): 4360-9. DOI: 10.1158/0008-5472.Can-21-1138.
255. Nawrot TS, Torfs R, Fierens F, De Henauw S, Hoet PH, Van Kersschaever G, et al. Stronger Associations Between Daily Mortality and Fine Particulate Air Pollution in Summer Than in Winter: Evidence From a Heavily Polluted Region in Western Europe. Journal of Epidemiology and Community Health. 2007; 61(2): 146-9. DOI: 10.1136/jech.2005.044263.
256. Wichmann H, Peters A. Epidemiological Evidence of the Effects of Ultrafine Particle Exposure. Trans R Soc Lond. 2000; A358: 2751-69. DOI: 10.1098/rsta.2000.0682.
257. Araujo JA, Barajas B, Kleinman M, Wang X, Bennett BJ, Gong KW, et al. Ambient Particulate Pollutants in the Ultrafine Range Promote Early Atherosclerosis and Systemic Oxidative Stress. Circulation Research. 2008; 102(5): 589-96. DOI: doi:10.1161/CIRCRESAHA.107.164970.
258. Donaldson K, Li XY, MacNee W. Ultrafine (Nanometre) Particle Mediated Lung Injury. Journal of Aerosol Science. 1998; 29(5-6): 553-60. DOI: 10.1016/S0021-8502(97)00464-3.
259. Penttinen P, Timonen KL, Tiittanen P, Mirme A, Ruuskanen J, Pekkanen J. Number Concentration and Size of Particles in Urban Air: Effects on Spirometric Lung Function in Adult Asthmatic Subjects. Environmental Health Perspectives. 2001; 109(4): 319-23. DOI: 10.1289/ehp.01109319.
260. Wåhlin P, Palmgren F, Van Dingenen R, Raes F. Pronounced Decrease of Ambient Particle Number Emissions From Diesel Traffic in Denmark After Reduction of the Sulphur Content in Diesel Fuel. Atmospheric Environment. 2001; 35(21): 3549-52. DOI: 10.1016/S1352-2310(01)00066-8.
261. Donaldson K, Tran L, Jimenez LA, Duffin R, Newby DE, Mills N, et al. Combustion-Derived Nanoparticles: A Review of Their Toxicology Following Inhalation Exposure. Particle and Fibre Toxicology. 2005; 2(10). DOI: 10.1186/1743-8977-2-10.

262. Li N, Sioutas C, Cho A, Schmitz D, Misra C, Sempf J, et al. Ultrafine Particulate Pollutants Induce Oxidative Stress and Mitochondrial Damage. Environmental Health Perspectives. 2003; 111(4): 455-60. DOI: 10.1289/ehp.6000.
263. Harrison RM, Shi JP, Xi S, Khan A, Mark D, Kinnersley R, et al. Measurement of Number, Mass, and Size Distribution of Particles in the Atmosphere. Philosophical Transactions of the Royal Society A: Mathematical, Physical and Engineering Sciences. 2000; 358(1775): 2567-80. DOI: 10.1098/rsta.2000.0669.
264. Hughes LS, Cass GR, Gone J, Ames M, Olmez I. Physical and Chemical Characterization of Atmospheric Ultrafine Particles in the Los Angeles Area. Environmental Science and Technology. 1998; 32(9): 1153-61. DOI: 10.1021/es970280r.
265. Aboh IJK, Henriksson D, Laursen J, Lundin M, Pind N, Lindgren ES, et al. EDXRF Characterisation Of Elemental Contents in PM2.5 in a Medium-Sized Swedish City Dominated by a Modern Waste Incineration Plant. X-Ray Spectrometry. 2007; 36(2): 104-10. DOI: 10.1002/xrs.947.
266. Li Z, Guan J, Yang X, Lin CH. Source Apportionment of Airborne Particles in Commercial Aircraft Cabin Environment: Contributions From Outside and Inside of Cabin. Atmospheric Environment. 2014; 89: 119-28. DOI: 10.1016/j.atmosenv.2014.01.042.
267. Michaelis S, Loraine T, Howard CV. Ultrafine Particle Levels Measured On-Board Short-Haul Commercial Passenger Jet Aircraft. Environmental Health. 2021; 20(89): 1-14. DOI: 10.1186/s12940-021-00770-7.
268. Fushimi A, Saitoh K, Fujitani Y, Takegawa N. Identification of Jet Lubrication Oil as a Major Component of Aircraft Exhaust Nanoparticles. Atmospheric Chemistry and Physics. 2019; 19(9): 6389-99. DOI: 10.5194/acp-19-6389-2019.
269. Lammers A, Janssen NAH, Boere AJF, Berger M, Longo C, Vijverberg SJH, et al. Effects Of Short-Term Exposures To Ultrafine Particles Near An Airport In Healthy Subjects. Environment International. 2020; 141. DOI: 10.1016/j.envint.2020.105779.
270. Bendtsen KM, Bengtsen E, Saber AT, Vogel U. A Review of Health Effects Associated with Exposure to Jet Engine Emissions in and Around Airports. Environmental Health : A Global Access Science Source. 2021; 20(1): 10. DOI: 10.1186/s12940-020-00690-y.
271. Bendtsen KM, Brostrøm A, Koivisto AJ, Koponen I, Berthing T, Bertram N, et al. Airport Emission Particles: Exposure Characterization and Toxicity Following Intratracheal Instillation in Mice. Particle and Fibre Toxicology. 2019; 16(1): 1-23. DOI: 10.1186/s12989-019-0305-5.
272. El Rahman HAA, Salama M, Gad El-Hak SA, El-Harouny MA, ElKafrawy P, Abou-Donia MB. A Panel of Autoantibodies Against Neural Proteins as Peripheral Biomarker for Pesticide-Induced Neurotoxicity. Neurotoxicity Research. 2018; 33(2): 316-36. DOI: 10.1007/s12640-017-9793-y.
273. Wüthrich B. Allergic and Intolerance Reactions to Wine. Allergol Select. 2018; 2(1): 80-8. DOI: 10.5414/alx01420e.
274. Lamba JK, Lin YS, Schuetz EG, Thummel KE. Genetic contribution to variable human CYP3A-mediated metabolism. Adv Drug Deliv Rev. 2002; 54(10): 1271-94. DOI: 10.1016/s0169-409x(02)00066-2.
275. Lu WJ, Ferlito V, Xu C, Flockhart DA, Caccamese S. Enantiomers of Naringenin as Pleiotropic, Stereoselective Inhibitors of Cytochrome P450 Isoforms. Chirality. 2011; 23(10): 891-6. DOI: 10.1002/chir.21005.
276. Humbert R, Adler DA, Disteche CM, Hassett C, Omiecinski CJ, Furlong CE. The Molecular Basis of the Human Serum Paraoxonase Activity Polymorphism. Nat Genet. 1993; 3(1): 73-6. DOI: 10.1038/ng0193-73.
277. Li WF, Costa LG, Richter RJ, Hagen T, Shih DM, Tward A, et al. Catalytic efficiency determines the in-vivo efficacy of PON1 for detoxifying organophosphorus compounds. Pharmacogenetics. 2000; 10(9): 767-79. DOI: 10.1097/00008571-200012000-00002.

278. Hosokawa M, Endo T, Fujisawa M, Hara S, Iwata N, Sato Y, et al. Interindividual variation in carboxylesterase levels in human liver microsomes. Drug Metabolism and Disposition. 1995; 23(10): 1022. PMCID: 8654188.
279. Delacour H, Dedome E, Courcelle S, Hary B, Ceppa F. Butyrylcholinesterase deficiency. Ann Biol Clin (Paris). 2016; 74(3): 279-85. DOI: 10.1684/abc.2016.1141.
280. Stevens RC, Suzuki SM, Cole TB, Park SS, Richter RJ, Furlong CE. Engineered recombinant human paraoxonase 1 (rHuPON1) purified from Escherichia coli protects against organophosphate poisoning. Proc Natl Acad Sci U S A. 2008; 105(35): 12780-4. DOI: 10.1073/pnas.0805865105.
281. Van Netten C. Analysis and Implications of Aircraft Disinsectants. Science of the Total Environment. 2002; 293: 257-62. DOI: 10.1016/S0048-9697(02)00036-0.
282. Naughton SX, Hernandez CM, Beck WD, Poddar I, Yanasak N, Lin PC, et al. Repeated Exposures to Diisopropylfluorophosphate Result in Structural Disruptions of Myelinated Axons and Persistent Impairments of Axonal Transport in the Brains of Rats. Toxicology. 2018; 406-407: 92-103. DOI: 10.1016/j.tox.2018.06.004.
283. COT. COT Statement 2007/06. Statement on the Review of the Cabin Air Environment, Ill-Health in Aircraft Crews and the Possible Relationship to Smoke/Fume Events in Aircraft. London: Committee Of Toxicity 2007. https://webarchive.nationalarchives.gov.uk/ukgwa/20200803134318/https://cot.food.gov.uk/cotstatements/cotstatementsyrs/cotstatements2007/cotstatementbalpa0706. Accessed 2 December 2022.
284. European Commission. Call For Tenders No. Move C2/2016-363 -Research Study: Investigation of the Quality Level of the Air Inside the Cabin of Large Transport Aeroplanes and its Health Implication. Tender Specifications. Brussels: European Commission - DG Mobility and Transport 2016. https://perma.cc/2ZLM-R6P2.
285. EPA. Memorandum: Guidance: Waiver Criteria for Multiple-Exposure Inhalation Toxicity Studies. HED SOP 2002.01 August 15, 2002. United States Environmental Protection Agency; 2002. https://perma.cc/D8FS-HS5G.
286. Honeywell. Honeywell Engineering Investigation Report - Customer Bleed Air Testing Engine Model Alf502R-5- Engine Serial Number LF 05311. Report No. 21-11156, 3 March 2000; Including Honeywell Air Quality Test For LF 502 Engine, S/N 5311, Test Cell 956 - December,1999. Phoenix: Honeywell; 2000.
287. SHK. Report Rl 2001: 41e. Incident Onboard Aircraft Se-Dre. Sweden, 12 November 1999. Stockholm, Sweden: Statens Haverikommission (SHK) Board of Accident Investigation; 2001 23 November 2001. https://www.havkom.se/assets/reports/rl2001_41e.pdf. Accessed 5 December 2022.
288. Rosenberger W. Effect of Charcoal Equipped HEPA Filters on Cabin Air Quality in Aircraft: A Case Study Including Smell Event Related In-Flight Measurements. Building and Environment. 2018; 143: 358-65. DOI: 10.1016/j.buildenv.2018.07.031.
289. Houtzager M, Havermans J, Bos J. Onderzoek Naar Aanwezigheid En Concentratie Van Tricresylfosfaten In De Cockpits Van KLM Boeing 737 Toestellen Tijdens Normale Operationele Condities. Investigation of Presence and Concentration of Tricesylphosphates in Cockpits of KLM Boeing 737 Aircraft During Normal Operational Conditions. TNO; 2013. TNO 2013 R11976. https://www.eerstekamer.nl/overig/20140820/tno_rapport_over_onderzoek_naar/document. Accessed 5 December 2022.
290. Kojima H, Takeuchi S, Itoh T, Iida M, Kobayashi S, Yoshida T. In Vitro Endocrine Disruption Potential Of Organophosphate Flame Retardants Via Human Nuclear Receptors. Toxicology. 2013; 314(1): 76-83. DOI: 10.1016/j.tox.2013.09.004.
291. Liu X, Ji K, Choi K. Endocrine Disruption Potentials of Organophosphate Flame Retardants and Related Mechanisms in H295R And MVIN Cell Lines and in Zebrafish. Aquatic Toxicology. 2012; 114-115: 173-81. DOI: 10.1016/j.aquatox.2012.02.019.

292. Diamanti-Kandarakis E, Bourguignon JP, Giudice LC, Hauser R, Prins GS, Soto AM, et al. Endocrine-Disrupting Chemicals: An Endocrine Society Scientific Statement. Endocrine Reviews. 2009; 30(4): 293-342. DOI: 10.1210/er.2009-0002.
293. Ji X, Li N, Ma M, Rao K, Yang R, Wang Z. Tricresyl phosphate isomers exert estrogenic effects via G protein-coupled estrogen receptor-mediated pathways. Environ Pollut. 2020; 264: 114747. DOI: 10.1016/j.envpol.2020.114747.
294. Bradford Hill A. The Environment and Disease: Association or Causation? Proceedings of the Royal Society of Medicine. 1965; 58(5): 295-300. PMCID: 1898525. https://www.ncbi.nlm.nih.gov/pmc/articles/PMC1898525/.
295. Vandenbroucke JP, Broadbent A, Pearce N. Causality and Causal Inference in Epidemiology: The Need for a Pluralistic Approach. International Journal of Epidemiology. 2016; 45(6): 1776-86. DOI: 10.1093/ije/dyv341.
296. Broadbent A, Vandenbroucke JP, Pearce N. Response: Formalism or pluralism? A reply to commentaries on 'Causality and causal inference in epidemiology'. Int J Epidemiol. 2016; 45(6): 1841-51. DOI: 10.1093/ije/dyw298.
297. Gee D. Associations and Causation : A Bradford Hill Approach to Aerotoxic Syndrome. International Aircraft Cabin Air Conference 2017; London. J Health and Pollution; 2019. p. S32-S7. DOI: 10.5696/2156-9614-9.24.191201.
298. Schaumburg HH. Human Neurotoxic Disease. In: Spencer P, Schaumburg H, editors. Experimental and Clinical Neurotoxicology. 2nd ed. Oxford: Oxford University Press; 2000. p. 55-82. ISBN: 9780195084771.
299. Abou-Donia MB, Krengel MH, Lapadula ES, Zundel CG, LeClair J, Massaro J, et al. Sex-Based Differences In Plasma Autoantibodies To Central Nervous System Proteins In Gulf War Veterans Versus Healthy And Symptomatic Controls. Brain Sci. 2021; 11(2): 148. DOI: 10.3390/brainsci11020148.
300. Abou-Donia MB, Lapadula ES, Krengel MH, Quinn E, LeClair J, Massaro J, et al. Using Plasma Autoantibodies of Central Nervous System Proteins to Distinguish Veterans with Gulf War Illness from Healthy and Symptomatic Controls. Brain Sci. 2020; 10(9): 610. DOI: 10.3390/brainsci10090610.
301. Abou-Donia MB, Lieberman A, Curtis L. Neural autoantibodies in patients with neurological symptoms and histories of chemical/mold exposures. Toxicology and Industrial Health. 2018; 34(1): 44-53. DOI: 10.1177/0748233717733852.
302. Abou-Donia MB, Suliman HB, Siniscalco D, Antonucci N, ElKafrawy P, Brahmajothi MV. De Novo Blood Biomarkers in Autism: Autoantibodies Against Neuronal and Glial Proteins. Behavioral Sciences. 2019; 9(5): 47. DOI: 10.3390/bs9050047.
